# Synthesis of novel series of 3,5-disubstituted imidazo[1,2-*d*] [1,2,4]thiadiazoles involving S_N_Ar and Suzuki–Miyaura cross-coupling reactions†

**DOI:** 10.1039/d1ra07208k

**Published:** 2022-02-23

**Authors:** Clémentine Pescheteau, Matthieu Place, Alexandru Sava, Lea Nunes, Lenuta Profire, Sylvain Routier, Frédéric Buron

**Affiliations:** Institut de Chimie Organique et Analytique, ICOA, UMR CNRS 7311, Université d’Orléans Orléans France frederic.buron@univ-orleans.fr; Department of Analytical Chemistry, Faculty of Pharmacy, “Grigore T. Popa” University of Medicine and Pharmacy of Iasi 16 University Street 700115 Iasi Romania; Department of Pharmaceutical Chemistry, Faculty of Pharmacy, “Grigore T. Popa” University of Medicine and Pharmacy of Iasi 16 University Street 700115 Iasi Romania

## Abstract

The first access to 3,5-disubstituted imidazo[1,2-*d*][1,2,4]thiadiazole derivatives is reported. The series were generated from 2-mercaptoimidazole, which afforded the key intermediate bearing two functional positions. The S_N_Ar reactivity toward tosyl release at the C-3 position was investigated and a regioselective electrophilic iodination in C-5 position was performed to allow a novel C–C bond using Suzuki–Miyaura reaction. Palladium-catalyzed cross-coupling conditions were optimized. A representative library of various boronic acids was employed to establish the scope and limitations of the method. To complete this methodological study, the influence of the nature of the C-3 imidazo[1,2-*d*][1,2,4]thiadiazole substitutions on the arylation in C-5 was investigated.

## Abstract 2

A convenient design of 3,5-disubstituted imidazo[1,2-*d*][1,2,4]thiadiazoles is reported from 2-mercaptoimidazole, which afforded a versatile platform that was then used to access a variety of original heterocycles.

## Graphical abstract

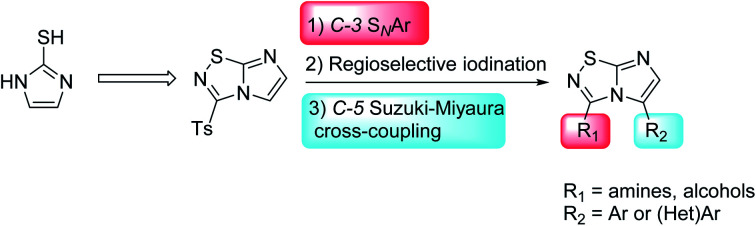

## Introduction

For the past few decades, sulfur-containing [5,5] fused ring systems with a bridgehead nitrogen have received considerable attention in the drug discovery field due to their interesting biological activities.^1–6^ For example, structures containing them have been reported in various therapeutic anticancer^7–9^ and antitubercular^10^ agents, and for cardiovascular treatments.^11^ Moreover, other representative molecules have demonstrated their potential in the treatment of neurodegenerative disorders.^12^ For these reasons, this heterocyclic family plays an increasingly important role in exploring uncovered regions of chemical space for the discovery of new biologically active drugs.

However, this exploration remains underdeveloped when we consider the sub-family bearing a sulfur–nitrogen bond.^13–19^ The main reasons are the lack of knowledge about their formation, reactivity or how to successfully position the desired substituents step by step. There has therefore been tremendous interest in overcoming this major hindrance in order to increase the molecular diversity around these series and develop highly original cores for the design of future original bioactive molecules.

For several years, our group has disclosed efficient methodologies to selectively functionalize bicyclic [5,5] heterocycles such as [1,2,4]triazolo[3,4-*b*][1,3,4]thiadiazoles,^20^ thiazolo[3,2-*b*][1,2,4]triazoles^21^ and more specially, imidazo[2,1-*b*][1,3,4]thiadiazoles.^22–26^ The latter have proven applicable to the discovery of a wide variety of biological molecules. Surprisingly, one of the isomers, the imidazo[1,2-*d*][1,2,4]thiadiazole core, has seldom been described, and is reported in only a few references, where it has shown its potential as a therapeutic agent, especially as a Factor XIIIa inhibitor.^27–31^ It is therefore of interest to provide a synthetic platform including a [5,5] nitrogen bridge heterocycle which would open the route to original biological compounds.

**Fig. 1:**
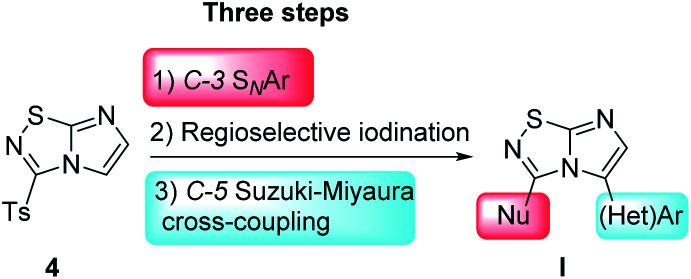
Access to 3,5-disubstituted imidazo[1,2-*d*][1,2,4] thiadiazole I from the versatile platform 4.

In order to build C-3 and C-5 disubstituted imidazo[1,2-*d*][1,2,4]thiadiazole derivatives, we developed a straightforward strategy which included from a versatile platform 4, a C-3 nucleophilic aromatic substitution followed by a C-5 iodination/Suzuki–Miyaura sequence. We report herein an unprecedented synthesis of 3,5-disubstituted-imidazo[1,2-*d*][1,2,4]thiadiazole I, the optimization of the experimental conditions and finally the scope of both reactions on these two selected positions (Fig. 1).

## Results and discussion

**Scheme 1:**
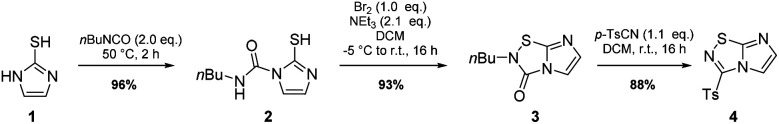
Access to versatile tosyl imidazo[1,2-*d*][1,2,4]thiadiazole platform 4.

First, we focused our attention on the tosyl platform 4, which can be prepared by using three short steps from commercially available 2-mercaptoimidazole.^27^ The condensation of 1 and *n*-butyl isocyanate led to amide 2 in a near quantitative yield. The oxidative ring closure in presence of bromine and triethylamine afforded the [5,5] fused bicyclic heterocycle 3 in an excellent 93% yield. To finish, a ring-opening/ring-closure sequence in presence of tosyl cyanide led to 4 in 88% yield (Scheme 1).

To tackle the usefulness of 4 as a building block and taking advantage of the tosyl as leaving group, we began by the C-3 functionalization using a S_N_Ar reaction. Using the Leung–Toum conditions,^27^ derivative 5 was synthesized with *n*-propylamine as nucleophile and Et_3_N as base, in toluene at r.t. after 4 hours in a 91% yield (Table 1, entry 1). After the successfully accomplished condensation of a primary aliphatic amine with 4, we next explored the scope of this method by treating the versatile platform 4 with other types of amines. The use of cycloalkylamines such as cycloproyl- or cyclohexyl-amine decreased the yield to 65% (entries 3 and 4). With *N*-methylpropylamine, the efficiency of the reaction was maintained (entry 2 *vs.* 1) while with other secondary cyclic amines such as piperidine or *N*-methylpiperazine, the efficiency significantly diminished (entries 5, 6). Fortunately, when morpholine was condensed with 4, the S_N_Ar reaction led to compound 11 in an excellent 94% yield (entry 7). Interestingly, we obtained a yield of 74% with the less nucleophilic benzylamine (entry 8) but no reaction was observed with aniline (entry 9). To achieve this investigation, we switched to alkoxides as nucleophiles (entries 10, 11) and all the attempted compounds were isolated in near quantitative yields.

**Table 1:** Synthesis of 5–15

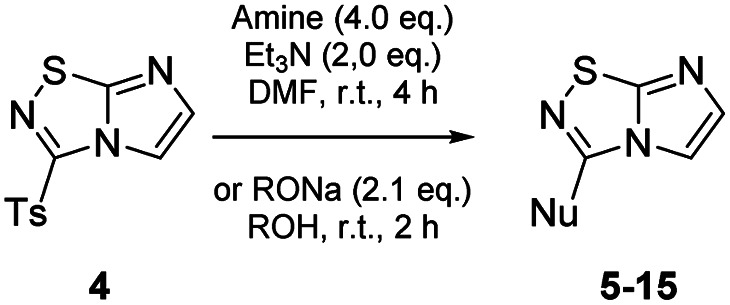			
Entry	Nucleophile	Product	Cpd, yield^a^
1	*n*-Propylamine	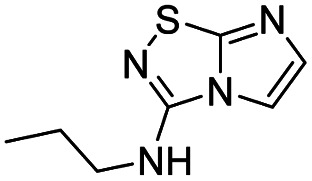	5, 91%
2	*N*-Methyl propylamine	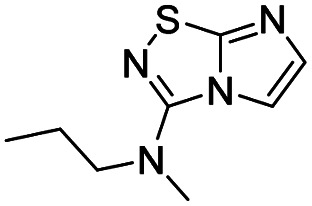	6, 92%
3	Cyclopropyl amine	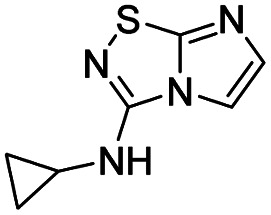	7, 65%
4	Cyclohexyl amine	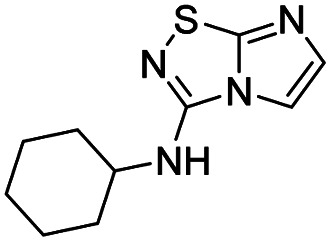	8, 65%
5	Piperidine	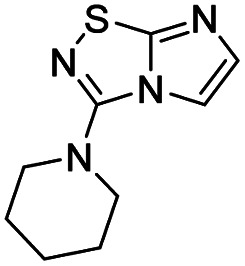	9, 59%
6	*N-*Methyl piperazine	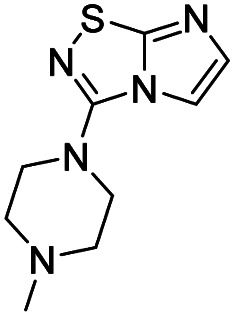	10, 70%
7	Morpholine	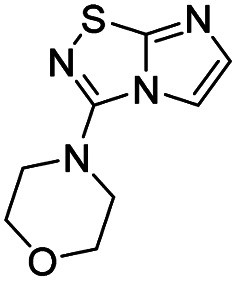	11, 94%
8	Benzylamine	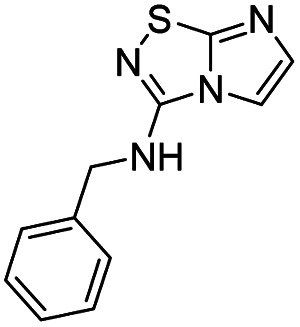	12, 74%
9	Aniline	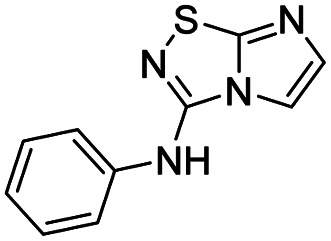	13, N.D.^b^
10	Sodium methoxide	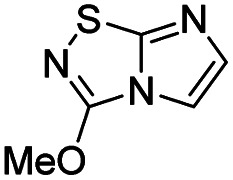	14, 99%
11	Sodium ethoxide	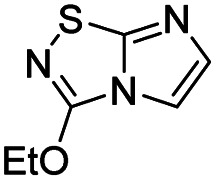	15, 95%

a Cpd: compound number; yield is indicated as isolated product.

b Not detected.

Selective halogenation in C-5 position with *N*-bromo or *N*-iodosuccinimide in DMF at r.t. was performed and showed a better reactivity for the introduction of iodine atom (Table 2 entries 22, 24*versus*26 and 27). The scope of the reaction was studied with compounds 5–11 to afford derivatives 16–27 (Table 2) without any significant problems as iodo derivatives were mainly isolated in satisfying yields, except in the case of 18 and 21 for which the purification was more problematic.

**Table 2:** Synthesis of 16–27

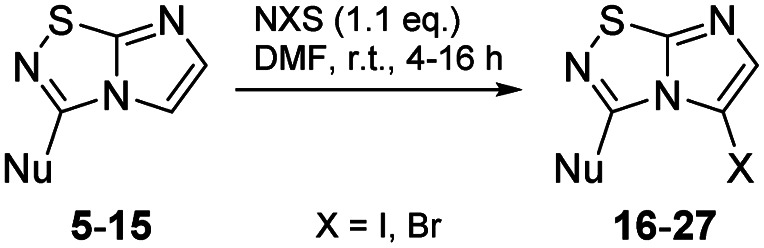					
Entry	Product	Cpd, yield^a^	Entry	Product	Cpd, yield^a^
1	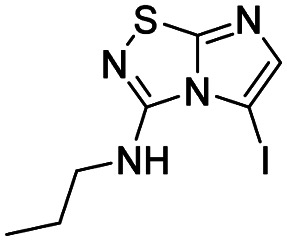	16, 57%	7	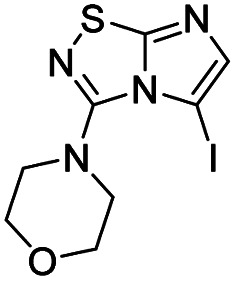	22, 92%
2	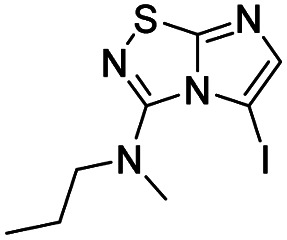	17, 62%	8	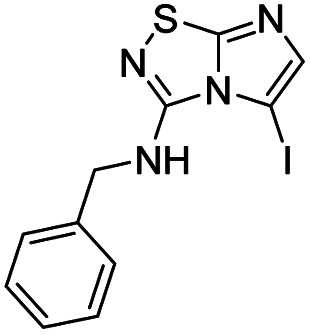	23, 59%
3	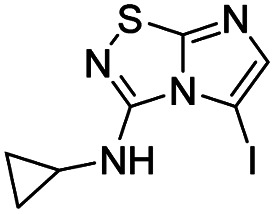	18, 7%	9	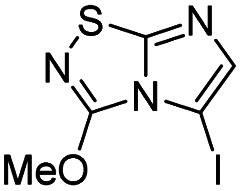	24, 83%
4	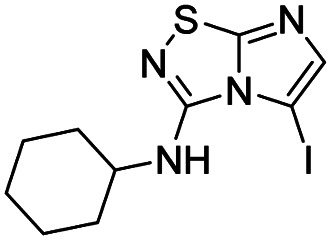	19, 84%	10	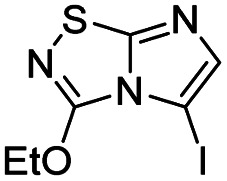	25, 89%
5	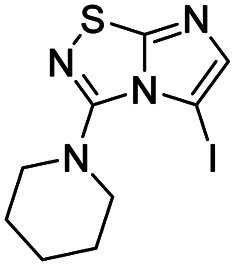	20, 62%	11	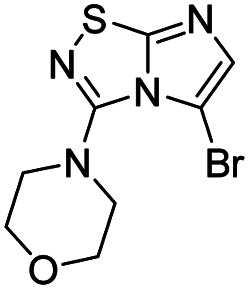	26, 34%
6	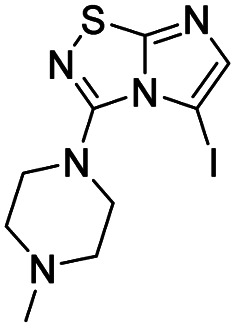	21, 16%	12	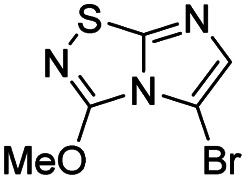	27, 76%

a Cpd: compound number; yield is indicated as isolated product.

With these compounds in hand, we then achieved the iodine displacement by Suzuki–Miyaura cross coupling as no C–C bond formation in C-5 position appears to be currently described on this skeleton. This prompted us to propose to the community a general and efficient catalytic system by optimizing the main reaction parameters (Table 3). First, we used 22 as starting material, Pd(PPh_3_)_4_ as the palladium source, Cs_2_CO_3_ as base, and dioxane as solvent under microwave irradiation during 1 h. With these conditions, the desired product 28 was isolated in a low but encouraging yield (33%, Table 3, entry 1). When the catalyst was switched for PdCl_2_(dppf).DCM, the reactivity was improved and the desired compound 28 was obtained in 47% yield. In the following experiment, we catalyzed the reaction with a bidentate palladium complex, which was formed by using a mixture of Pd(OAc)_2_ (10 mol%) and Xantphos (20 mol%). The reaction was achieved in only 1 h and product 28 was isolated in a satisfying yield of 60% (Table 3, entry 3). Changing the nature of the base indicated that the use of K_2_CO_3_ did not affect the reaction yield whereas K_3_PO_4_ partially reduced the reactivity.

**Table 3:** Optimization of conditions for the formation of 28

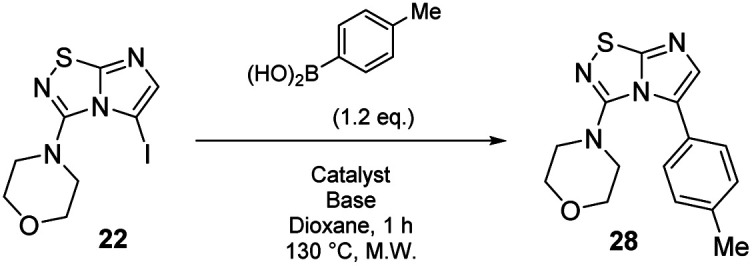			
Entry	Catalyst	Base	Yield^a^
1	Pd(PPh_3_)_4_ (0.1 eq.)	Cs_2_CO_3_, (2.0 eq.)	33%
2	PdCl_2_(dppf)·DCM (0.1 eq.)	Cs_2_CO_3_, (2.0 eq.)	47%
3	Pd(OAc)_2_ (0.1 eq.), xantphos (0.2 eq.)	Cs_2_CO_3_, (2.0 eq.)	60%
4	Pd(OAc)_2_ (0.1 eq.), xantphos (0.2 eq.)	K_2_CO_3_, (2.0 eq.)	60%
5	Pd(OAc)_2_ (0.1 eq.), xantphos (0.2 eq.)	K_3_PO_4_, (2.0 eq.)	36%

a Yield is indicated as isolated product.

Next, the scope and potential limitations of the Pd-coupling step were investigated by modulation of the boron derivatives (Table 4). The use of electron-rich or neutral phenyl boronic acids was well tolerated and furnished the derivatives 29 and 30 in good yields (entries 2 and 3). In contrast, the presence of electron-withdrawing substituents such as nitro or fluorine slightly decreased the efficiency of the reaction and compounds 34 and 35 were isolated in 40% and 38% yields, respectively. Next, we investigated the influence of steric hindrance using the methoxy position switch on the phenyl ring. While the *ortho* orientation induced a dramatic decrease in yield (34% *versus* 65% for 28), the *meta* orientation led to the desired compound in a 50% yield (entries 3–5). The only identified limit concerned the presence of labile hydrogens such as OH or NH, which totally inhibited the reaction (entries 6, 10 and 11). This constraint was easily removed by the use of a protective group such as THP for the phenol derivative (entry 6) and an aryl entity was successfully introduced in a good overall yield of 59% after a tandem sequence including the cross coupling reaction and the *in situ* deprotection. Finally, the introduction of electron-rich heterocycle was studied with thiophene-3-boronic acid, and the desired product 36 was isolated in a good 71% yield.

**Table 4:** Synthesis of 28–38

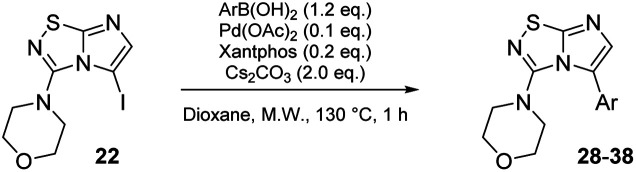					
Entry	Product	Cpd, yield^a^	Entry	Product	Cpd, yield^a^
1	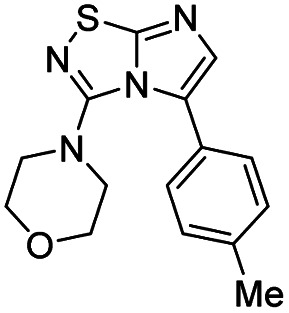	28, 60%	7	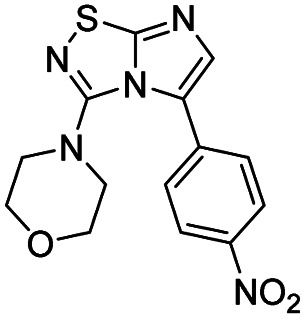	34, 40%
2	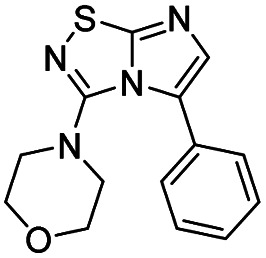	29, 72%	8	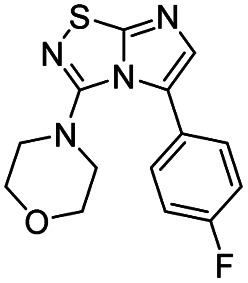	35, 38%
3	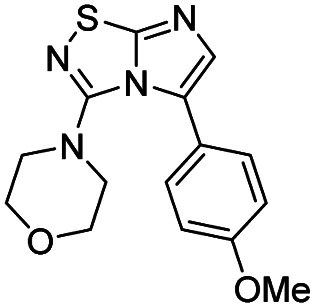	30, 65%	9	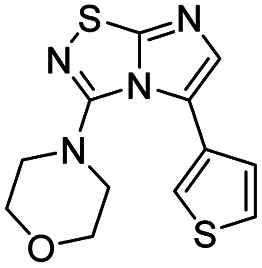	36, 71%
4	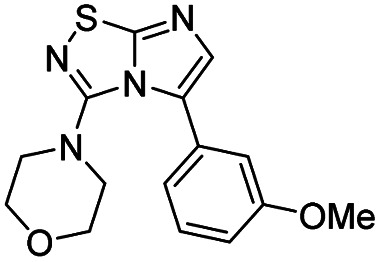	31, 50%	10	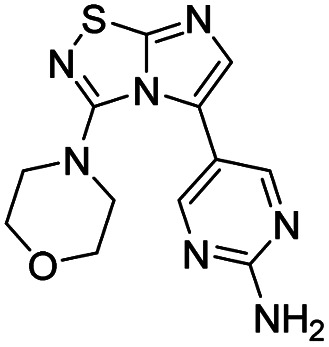	37, N.D.^b^
5	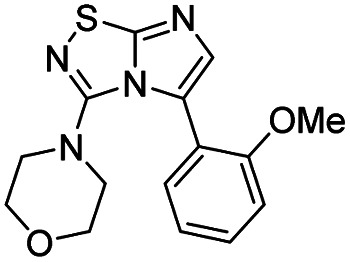	32, 34%	11	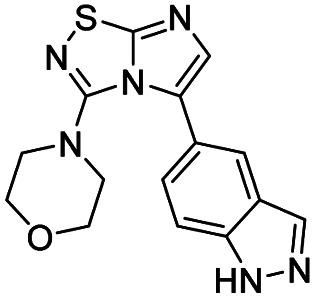	38, N.D.^b^
6	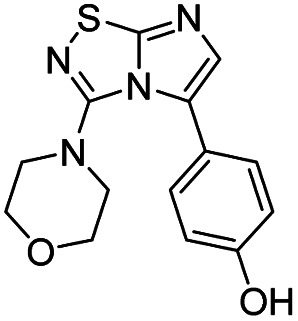	33, 59%^c^			

a Cpd: compound number; yield is indicated as isolated product.

b Not detected.

c Overall yield after deprotection of THP ether in AcOH/THF/H_2_O (4/2/1) at 40 °C, 16 h.

To complete this Suzuki–Miyaura study, we then evaluated the influence of the nature of the substituent in C-3 position (Table 5) and selected *p*-tolylboronic acid as the sole arylation partner. As previously described, the presence of a hydrogen on the C-5 nitrogen atom totally inhibited the catalytic cycle (entries 1, 3, 4 and 8) whereas its substitution restored the efficiency of the reaction (entry 2 *vs.* 1). In fact, whatever the nature of the tertiary amine (*i.e.* aliphatic or cyclic) in C-3 position, the C–N bond was efficiently generated and products were isolated in fairly good yields ranging from 56% to 71% (Table 5, entries 2, 5–7). To finish, we performed the cross coupling reaction in the presence of an alkyloxy in the C-3 position and proved that the final compound could be obtained in moderate yield (entries 9, 10) suggesting that electronic enrichment of the heterocycle played a major role in the result. Heck (with methyl acrylate) or Buchwald (with aniline) cross-coupling reactions are performed with this catalytic system and only 10% of conversion were obtained. This limitation prompted us to identify, in the future, a new catalytic system able to remove this limitation.

**Table 5:** Synthesis of 28, 39–47

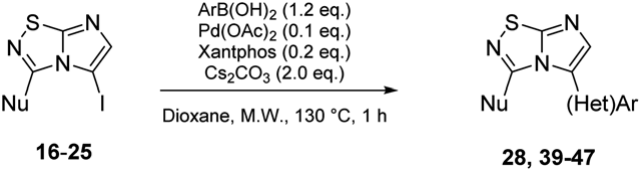		
Entry	Product	Cpd, yield^a^
1	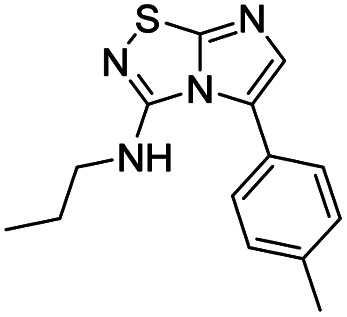	39, N.D.^b^
2	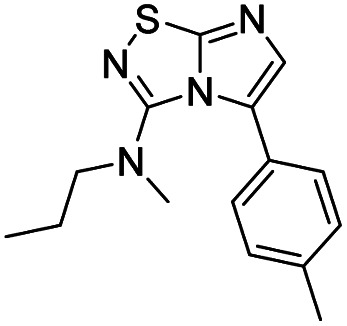	40, 71%
3	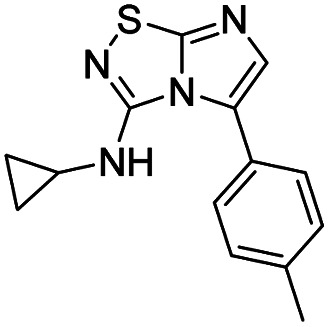	41, N.D.^b^
4	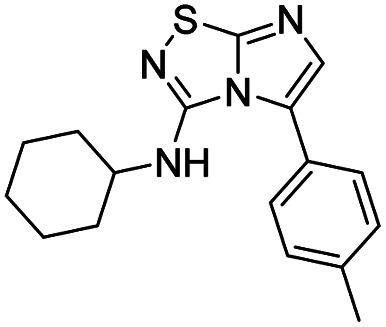	42, N.D.^b^
5	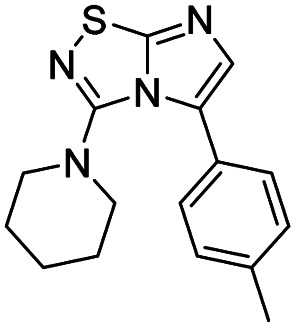	43, 70%
6	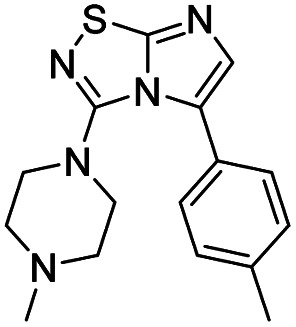	44, 56%
7	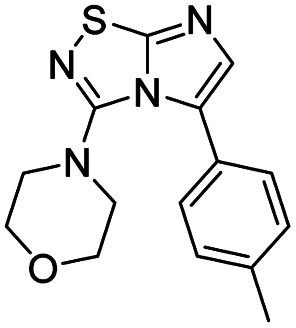	28, 60%
8	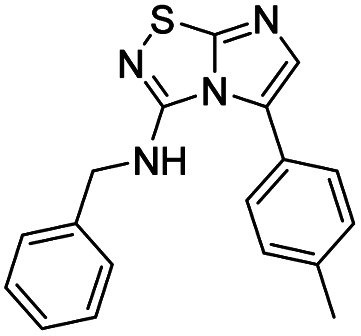	45, N.D.^b^
9	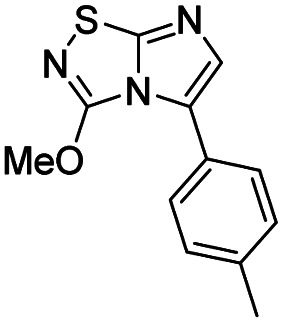	46, 36%
10	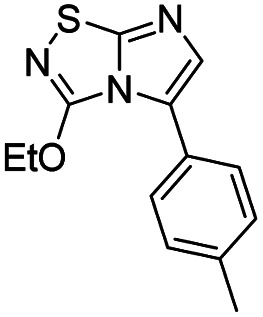	47, 60%

a Cpd: compound number; Yield is indicated as isolated product.

b Not detected.

## Conclusions

In summary, the quick access to 3,5-disubstituted imidazo[1,2-*d*][1,2,4]thiadiazole has been described herein. Aminated or alkoxyl groups were introduced at the C-3 position of the imidazo[1,2-*d*][1,2,4]thiadiazole platform using the S_N_Ar strategy. A large variety of amines or alkoxides was achieved, giving, after regioselective electrophilic iodination in C-5 position, access to perform a C–C bond formation. The efficiency of the Suzuki–Miyaura reactions in C-5 position was shown with a large panel of boronic acids. This work allows access to a novel class of 3,5 disubstituted imidazo[1,2-*d*][1,2,4]thiadiazoles which will undoubtedly have a major impact on the further synthesis of new bioactive compounds that contain the rare imidazo[1,2-*d*][1,2,4]thiadiazole scaffold as the central skeleton. Efforts to achieve these objectives are currently in progress.

## Experimental section

### Materials and methods

^1^H NMR and ^13^C NMR spectra were recorded on a Bruker DPX 250 or 400 MHz instrument using CDCl_3_ and DMSO-*d*_6_. The chemical shifts are reported in parts per million (*δ* scale), and all coupling constant (*J*) values are reported in hertz. The following abbreviations were used for the multiplicities: s (singlet), d (doublet), t (triplet), q (quartet), p (pentuplet), m (multiplet), sext (sextuplet), and dd (doublet of doublets). Melting points are uncorrected. IR absorption spectra were obtained on a PerkinElmer PARAGON 1000 PC, and the values are reported in inverse centimeters. HRMS spectra were acquired in positive mode with an ESI source on a Q-TOF mass by the “Fédération de Recherche” ICOA/CBM (FR2708) platform. Monitoring of the reactions was performed using silica gel TLC plates (silica Merck 60 F 254). Spots were visualized by UV light (254 nm and 356 nm). Column chromatography was performed using silica gel 60 (0.063–0.200 mm, Merck.). Microwave irradiation was carried out in sealed vessels placed in a Biotage Initiator or Biotage Initiator+ system (400 W maximum power). The temperatures were measured externally by IR. Pressure was measured by a non-invasive sensor integrated into the cavity lid. All reagents were purchased from commercial suppliers and were used without further purification.

### Synthetic procedures

#### General procedure A: S_N_Ar of tosylated imidazo[1,2-*d*][1,2,4]thiadiazole derivative

3-(4-Methylbenzenesulfonyl)imidazo[1,2-*d*][1,2,4]thiadiazole 4 (1.0 eq.) was dissolved in DMF (10 mL for 1.0 g) under an argon atmosphere then triethylamine (2.0 eq.) and corresponding amine (4.0 eq.) were added. The reaction was stirred at room temperature for 4 h then quenched by water addition. The mixture was extracted 3 times with ethyl acetate (3 × 15 mL) and combined organic phases were dried over MgSO_4_ and filtered. After concentration under reduced pressure, the residue was purified by flash chromatography on silica gel.

#### General procedure B: iodination or bromination of imidazo[1,2*-d*][1,2,4]thiadiazole on C-5 position

Corresponding imidazo[1,2*-d*][1,2,4]thiadiazole 5–15 (1.0 eq.) was dissolved in dry DMF then *N*-iodosuccinimide or *N*-bromosuccinimide (1.1 eq.) was added. After 4–16 h stirring at room temperature away from light, the mixture was diluted with water, then extracted twice with ethyl acetate (2 × 15 mL). Combined organic phases were washed with a 10% aqueous solution of sodium thiosulfate (20 mL), and twice with brine (2 × 20 mL). The solution was dried over MgSO_4_, filtered, concentrated under reduced pressure, and then the residue was purified by flash chromatography on silica gel.

#### General procedure C: Suzuki–Miyaura cross-coupling in C-5 position *of* imidazo[1,2*-d*][1,2,4]thiadiazole

A solution of corresponding 5-iodoimidazo[1,2-*d*][1,2,4]thiadiazole 16–25 (1.0 eq.), cesium carbonate (2.0 eq.), and corresponding boronic acid (1.2 eq.) in 1,4-dioxane (0.1 M) was degassed by argon bubbling for 15 min. Palladium diacetate (0.1 eq.) and Xantphos (0.2 eq.) were added and the mixture was heated at 130 °C for 1 h under microwave irradiation. The reaction mixture was then directly purified by flash chromatography on silica gel.

#### *N*-Propylimidazo[1,2-*d*][1,2,4]thiadiazol-3-amine (5)

The reaction was carried out as described in general procedure A using 4 (100 mg, 0.36 mmol, 1.0 eq.), *n*-propylamine (110 μL, 1.43 mmol, 4.0 eq.), triethylamine (100 μL, 0.72 mmol, 2.0 eq.) in dry DMF (5 mL). The crude mixture was purified by flash chromatography on silica gel (CH_2_Cl_2_/MeOH, 95/5) to afford 5 as an amorphous solid (60 mg, 91%). *R*_f_ (PE/EtOAc, 1/1): 0.23. ^1^H NMR (400 MHz, CDCl_3_): *δ* (ppm) 7.41 (d, *J* = 1.6 Hz, 1H, H_5_), 7.30 (d, *J* = 1.5 Hz, 1H, H_6_), 6.03 (t, *J* = 5.6 Hz, 1H, NH), 3.47–3.41 (m, 2H, *CH*_2_CH_2_CH_3_), 1.70 (h, *J* = 7.4 Hz, 2H, CH_2_*CH*_2_CH_3_), 0.98 (t, *J* = 7.4 Hz, 3H, CH_2_CH_2_*CH*_3_). ^13^C NMR (101 MHz, CDCl_3_): *δ* (ppm) 159.1 (C_q_), 144.0 (C_q_), 137.2 (C_6_), 109.5 (C_5_), 44.6 (*CH*_2_CH_2_CH_3_), 22.7 (CH_2_*CH*_2_CH_3_), 11.5 (CH_2_CH_2_*CH*_3_). IR (ATR diamond): *ν* (cm^−1^) 3219, 3134, 3050, 2934, 1614, 1453, 1331, 1125, 732. HRMS (EI-MS) *m*/*z* calcd for C_7_H_11_N_4_S: 183.0699 [M + H]^+^, found: 183.0699.

#### *N*-Methyl-*N*-propylimidazo[1,2-*d*][1,2,4]thiadiazol-3-amine (6)

The reaction was carried out as described in general procedure A using 4 (100 mg, 0.36 mmol, 1.0 eq.), *N*-methylpropylamine (150 μL, 1.43 mmol, 4.0 eq.), triethylamine (100 μL, 0.72 mmol, 2.0 eq.) in dry DMF (5 mL). The crude mixture was purified by flash chromatography on silica gel (EtOAc/PE, 6/4) to afford 6 as a yellow oil (65 mg, 92%). *R*_f_ (PE/EtOAc, 6/4): 0.52. ^1^H NMR (250 MHz, CDCl_3_): *δ* (ppm) 7.44 (s, 1H, H_5_), 7.31 (s, 1H, H_6_), 3.46–3.31 (m, 2H, *CH*_2_CH_2_CH_3_), 3.12 (d, *J* = 1.0 Hz, 3H, CH_3_), 1.68 (h, *J* = 7.4 Hz, 2H, CH_2_*CH*_2_CH_3_), 0.94 (t, *J* = 7.5 Hz, 3H, CH_2_CH_2_*CH*_3_). ^13^C NMR (63 MHz, CDCl_3_): *δ* (ppm) 160.6 (C_q_), 146.9 (C_q_), 137.6 (C_6_), 112.1 (C_5_), 53.4 (*CH*_2_CH_2_CH_3_), 37.5 (CH_3_), 21.0 (CH_2_*CH*_2_CH_3_), 11.1 (CH_2_CH_2_*CH*_3_). IR (ATR diamond): *ν* (cm^−1^) 3134, 3030, 2914, 1641, 1455, 1341, 1130, 734. HRMS (EI-MS) *m*/*z* calcd for C_8_H_13_N_4_S: 197.0855 [M + H]^+^, found: 197.0857.

#### *N*-Cyclopropylimidazo[1,2-*d*][1,2,4]thiadiazol-3-amine (7)

The reaction was carried out as described in general procedure A using 4 (500 mg, 1.79 mmol, 1.0 eq.), 4-cyclopropylamine (0.50 mL, 7.16 mmol, 4.0 eq.), triethylamine (496 μL, 3.58 mmol, 2.0 eq.) in dry DMF (25 mL). The crude mixture was purified by flash chromatography on silica gel (CH_2_Cl_2_/MeOH, 98/2) to afford 7 as a white solid (210 mg, 65%). *R*_f_ (CH_2_Cl_2_/MeOH, 95/5): 0.52. Mp: 157–159 °C. ^1^H NMR (400 MHz, CDCl_3_): *δ* (ppm) 7.35 (d, *J* = 1.5 Hz, 1H, H_5_), 7.31 (d, *J* = 1.5 Hz, 1H, H_6_), 5.24 (s, 1H, NH), 2.97–2.84 (m, 1H, CH), 0.96–0.86 (m, 2H, CH_2_), 0.77–0.67 (m, 2H, CH_2_). ^13^C (101 MHz, CDCl_3_): *δ* (ppm) 159.4 (C_q_), 144.2 (C_q_), 137.8 (C_6_), 109.2 (C_5_), 24.6 (CH), 7.6 (2 × CH_2_). IR (ATR diamond): *ν* (cm^−1^)3172, 2987, 2896, 1599, 1556, 1454, 1358, 1332, 1300, 1127, 730. HRMS (EI-MS) *m*/*z* calcd for C_7_H_9_N_4_S: 181.0542 [M + H]^+^, found: 181.0545.

#### *N*-Cyclohexylimidazo[1,2-*d*][1,2,4]thiadiazol-3-amine (8)

The reaction was carried out as described in general procedure A using 4 (500 mg, 1.79 mmol, 1.0 eq.), cyclohexylamine (819 μL, 7.16 mmol, 4.0 eq.), triethylamine (496 μL, 3.58 mmol, 2.0 eq.) in dry DMF (25 mL). The crude mixture was purified by flash chromatography on silica gel (CH_2_Cl_2_/MeOH, 98/2) to afford 8 as a yellow solid (260 mg, 65%). *R*_f_ (CH_2_Cl_2_/MeOH, 95/5): 0.37. Mp: 180–182 °C. ^1^H NMR (400 MHz, CDCl_3_): *δ* (ppm) 7.35 (d, *J* = 1.5 Hz, 1H, H_5_), 7.28 (d, *J* = 1.5 Hz, 1H, H_6_), 4.62 (d, *J* = 7.6 Hz, 1H, NH), 3.81 (m, 1H, CH), 2.24–2.10 (m, 2H, CH_2_), 1.79 (m, 2H, CH_2_), 1.73–1.63 (m, 1H, CH), 1.52–1.36 (m, 2H, CH_2_), 1.36–1.20 (m, 3H, CH + CH_2_). ^13^C (101 MHz, CDCl_3_): *δ* (ppm) 159.0 (C_q_), 142.5 (C_q_), 137.6 (C_6_), 108.6 (C_5_), 51.8 (CH), 33.3 (2 × CH_2_), 25.5 (CH_2_), 24.09 (2 × CH_2_). IR (ATR diamond): *ν* (cm^−1^) 3259, 3103, 3062, 2924, 2855, 1604, 1550, 1457, 1294, 1106, 746. HRMS (EI-MS) *m*/*z* calcd for C_10_H_15_N_4_S: 223.1012 [M + H]^+^, found: 223.1011.

#### 3-(Piperidin-1-yl)imidazo[1,2-*d*][1,2,4]thiadiazole (9)

The reaction was carried out as described in general procedure A using 4 (500 mg, 1.79 mmol, 1.0 eq.), piperidine (707 μL, 7.16 mmol, 4.0 eq.), triethylamine (496 μL, 3.58 mmol, 2.0 eq.) in dry DMF (25 mL). The crude mixture was purified by flash chromatography on silica gel (CH_2_Cl_2_/MeOH, 98/2) to afford 9 as a white solid (220 mg, 59%). *R*_f_ (CH_2_Cl_2_/MeOH, 95/5): 0.52. Mp: 88–90 °C. ^1^H NMR (400 MHz, DMSO-*d*_6_): *δ* (ppm) 8.02 (s, 1H, H_5_), 7.37 (s, 1H, H_6_), 3.46–3.43 (m, 4H, 2 × CH_2_), 1.68–1.64 (m, 6H, 3 × CH_2_). ^13^C NMR (101 MHz, DMSO-*d*_6_): *δ* (ppm) 157.9 (C_q_), 147.3 (C_q_), 137.3 (C_6_), 113.6 (C_5_), 48.3 (2 × CH_2_), 24.7 (2 × CH_2_), 23.6 (CH_2_). IR (ATR diamond): *ν* (cm^−1^) 2939, 2838, 1567, 1489, 1419, 1408, 1375, 1282, 1261, 711. HRMS (EI-MS) *m*/*z* calcd for C_9_H_13_N_4_S: 209.0855 [M + H]^+^, found: 209.0853.

#### 3-(4-Methylpiperazin-1-yl)imidazo[1,2-*d*][1,2,4]thiadiazole (10)

The reaction was carried out as described in general procedure A using 4 (500 mg, 1.79 mmol, 1.0 eq.), 1-methyl piperazine (0.79 mL, 7.16 mmol, 4.0 eq.), triethylamine (496 μL, 3.58 mmol, 2.0 eq.) in dry DMF (25 mL). The crude mixture was purified by flash chromatography on silica gel (CH_2_Cl_2_/MeOH, 95/5) to afford 10 as a white solid (280 mg, 70%). *R*_f_ (CH_2_Cl_2_/MeOH, 95/5): 0.32. Mp: 119–121 °C. ^1^H NMR (400 MHz, CDCl_3_): *δ* (ppm) 7.41 (d, *J* = 1.6 Hz, 1H, H_5_), 7.37 (d, *J* = 1.6 Hz, 1H, H_6_), 3.56–3.49 (m, 4H, 2 × CH_2_), 2.65–2.54 (m, 4H, 2 × CH_2_), 2.37 (s, 3H, CH_3_). ^13^C (101 MHz, CDCl_3_): *δ* (ppm) 160.0 (C_q_), 147.1 (C_q_), 137.9 (C_6_), 111.5 (C_5_), 54.3 (2 × CH_2_), 47.9 (2 × CH_2_), 46.3 (CH_3_). IR (ATR diamond): *ν* (cm^−1^) 2841, 2807, 1568, 1489, 1415, 1277, 1135, 1003, 706. HRMS (EI-MS) *m*/*z* calcd for C_9_H_14_N_5_S: 224.0964 [M + H]^+^, found: 224.0967.

#### 3-(Morpholin-4-yl)imidazo[1,2*-d*][1,2,4]thiadiazole (11)

The reaction was carried out as described in general procedure A using 4 (100 mg, 0.36 mmol, 1.0 eq.), morpholine (125 μL, 1.43 mmol, 4.0 eq.), triethylamine (100 μL, 0.72 mmol, 2.0 eq.) in dry DMF (5 mL). The crude mixture was purified by flash chromatography on silica gel (CH_2_Cl_2_/MeOH, 98/2) to afford 11 as a white solid (71 mg, 94%). *R*_f_ (PE/EtOAc, 1/1): 0.41. Mp: 166–168 °C. ^1^H NMR (400 MHz, CDCl_3_): *δ* (ppm) 7.46–7.35 (m, 2H, H_5_ + H_6_), 3.93–3.82 (m, 4H, 2 × CH_2_), 3.53–3.41 (m, 4H, 2 × CH_2_). ^13^C NMR (101 MHz, CDCl_3_): *δ* (ppm) 160.11 (C_q_), 146.91 (C_q_), 137.9 (C_6_), 111.3 (C_5_), 66.2 (2 × CH_2_), 48.2 (2 × CH_2_). IR (ATR diamond): *ν* (cm^−1^) 3106, 2961, 2867, 1567, 1487, 1413, 1273, 1259, 1118, 855, 717. HRMS (EI-MS) *m*/*z* calcd for C_8_H_11_N_4_OS: 211.0648 [M + H]^+^, found: 211.0643.

#### *N*-Benzylimidazo[1,2-*d*][1,2,4]thiadiazol-3-amine (12)

The reaction was carried out as described in general procedure A using 4 (500 mg, 1.79 mmol, 1.0 eq.), benzylamine (0.78 mL, 7.16 mmol, 4.0 eq.), triethylamine (496 μL, 3.58 mmol, 2.0 eq.) in dry DMF (25 mL). The crude mixture was purified by flash chromatography on silica gel (PE/EtOAc, 5/5) to afford 12 as a white solid (306 mg, 74%). *R*_f_ (PE/EtOAc, 6/4): 0.17. Mp: 160–162 °C. ^1^H NMR (400 MHz, CDCl_3_): *δ* (ppm) 7.45–7.27 (m, 7H, 5 × H_Ar_ + H_5_ + H_6_), 5.90 (s, 1H, NH), 4.65 (d, *J* = 5.6 Hz, 2H, CH_2_). ^13^C (101 MHz, CDCl_3_): *δ* (ppm) 159.3 (C_q_), 143.4 (C_q_), 137.5 (C_6_), 129.0 (2 × CH_Ar_), 128.8 (C_q_), 128.2 (2 × CH_Ar_), 128.1 (C_Ar_), 109.3 (C_5_), 46.9 (CH_2_). IR (ATR diamond): *ν* (cm^−1^) 3218, 3033, 2929, 1601, 1538, 1453, 1327, 1295, 1097, 693. HRMS (EI-MS) *m*/*z* calcd for C_11_H_11_N_4_S: 231.0699 [M + H]^+^, found: 231.0700.

#### 3-Methoxyimidazo[1,2-*d*][1,2,4]thiadiazole (14)

To a solution of 4 (350 mg, 1.25 mmol, 1.0 eq.) in 10 mL of methanol was added NaOMe 25% in MeOH (590 μL, 2.62 mmol, 2.1 eq.). The solution was stirred at room temperature for 30 min, and then quenched by addition of water (10 mL). The mixture was extracted with EtOAc (3 × 50 mL), and combined organic phases were washed with brine (100 mL), dried over MgSO_4_, filtered and concentrated under vacuum. The crude mixture was purified by flash chromatography on silica gel (CH_2_Cl_2_/MeOH, 98/2) to afford 14 as a white solid (190 mg, 99%). *R*_f_ (PE/EtOAc, 1/1): 0.56. Mp: 187–189 °C. ^1^H NMR (250 MHz, CDCl_3_): *δ* (ppm) 7.38 (d, *J* = 1.5 Hz, 1H, H_5_), 7.33 (d, *J* = 1.5 Hz, 1H, H_6_), 4.20 (s, 3H, CH_3_). ^13^C NMR (63 MHz, CDCl_3_): *δ* (ppm) 158.2 (C_q_), 146.4 (C_q_), 137.8 (C_6_), 110.2 (C_5_), 57.9 (CH_3_). IR (ATR diamond): *ν* (cm^−1^) 3127, 2954, 1674, 1565, 1441, 1289, 1117, 959, 857. HRMS (EI-MS) *m*/*z* calcd for C_5_H_6_N_3_OS: 156.0226 [M + H]^+^, found: 156.0228.

#### 3-Ethoxyimidazo[1,2-*d*][1,2,4]thiadiazole (15)

To a solution of 4 (500 mg, 1.8 mmol, 1.0 eq.) in 10 mL of ethanol was added freshly prepared NaOEt 21% in EtOH (260 mg, 3.8 mmol, 2.1 eq.). The solution was stirred at room temperature for 2 h, and then quenched by addition of water (10 mL). The mixture was extracted with EtOAc (3 × 50 mL), and combined organic phases were washed with brine (100 mL), dried over MgSO_4_ and concentrated under vacuum. The crude mixture was purified by flash chromatography on silica gel (CH_2_Cl_2_/MeOH, 98/2) to afford 15 as a white solid (290 mg, 95%). *R*_f_ (PE/EtOAc, 1/1): 0.55. Mp: 193–195 °C. ^1^H NMR (250 MHz, CDCl_3_): *δ* (ppm) 7.39 (d, *J* = 1.5 Hz, 1H, H_5_), 7.33 (d, *J* = 1.4 Hz, 1H, H_6_), 4.61 (q, *J* = 7.1 Hz, 2H, CH_2_), 1.50 (t, *J* = 7.1 Hz, 3H, CH_3_). ^13^C NMR (63 MHz, CDCl_3_): *δ* (ppm) 157.8 (C_q_), 145.6 (C_6_), 137.3 (C_q_), 110.0 (C_5_), 66.8 (CH_2_), 14.2 (CH_3_). IR (ATR diamond): *ν* (cm^−1^) 3130, 2978, 1652, 1555, 1470, 1274, 1111, 1018, 950, 845. HRMS (EI-MS) *m*/*z* calcd for C_6_H_8_N_3_OS: 170.0383 [M + H]^+^, found: 170.0385.

#### 5-Iodo-*N*-propylimidazo[1,2-*d*][1,2,4]thiadiazol-3-amine (16)

The reaction was carried out as described in general procedure B using 5 (200 mg, 1.1 mmol, 1.0 eq.), *N*-iodosuccinimide (371 mg, 1.21 mmol, 1.1 eq.) in dry DMF (5 mL). The crude mixture was purified by flash chromatography on silica gel (PE/EtOAc, 1/1) to afford 16 as a white solid (193 mg, 57%). *R*_f_ (PE/EtOAc, 1/1): 0.41. Mp: 153–155 °C. ^1^H NMR (400 MHz, CDCl_3_): *δ* (ppm) 7.28 (s, 1H, H_6_), 5.66 (s, 1H, NH), 3.48 (q, *J* = 7.0, 5.5 Hz, 2H, *CH*_2_CH_2_CH_3_), 1.74 (h, *J* = 7.3 Hz, 2H, CH_2_*CH*_2_CH_3_), 1.04 (t, *J* = 7.4 Hz, 3H, CH_2_CH_2_*CH*_3_). ^13^C NMR (101 MHz, CDCl_3_): *δ* (ppm) 161.6 (C_q_), 144.2 (C_6_), 143.2 (C_q_), 50.9 (C_5_), 43.9 (*CH*_2_CH_2_CH_3_), 21.9 (CH_2_*CH*_2_CH_3_), 10.8 (CH_2_CH_2_*CH*_3_). IR (ATR diamond): *ν* (cm^−1^) 3386, 3114, 2966, 2877, 1588, 1519, 1433, 1355, 1120, 952, 847, 700. HRMS (EI-MS) *m*/*z* calcd for C_7_H_10_IN_4_S: 308.9665 [M + H]^+^, found: 308.9661.

#### 5-Iodo-*N*-methyl-*N*-propylimidazo[1,2-*d*][1,2,4]thiadiazol-3-amine (17)

The reaction was carried out as described in general procedure B using 6 (125 mg, 0.64 mmol, 1.0 eq.), *N*-iodosuccinimide (160 mg, 0.70 mmol, 1.1 eq.) in dry DMF (5 mL). The crude mixture was purified by flash chromatography on silica gel (PE/EtOAc, 6/4) to afford 17 as a yellow oil (128 mg, 62%). *R*_f_ (PE/EtOAc, 6/4): 0.64. ^1^H NMR (250 MHz, CDCl_3_): *δ* (ppm) 7.32 (s, 1H, H_6_), 3.27–3.10 (m, 2H, *CH*_2_CH_2_CH_3_), 2.91 (s, 3H, CH_3_), 1.66 (h, *J* = 7.4 Hz, 2H, CH_2_*CH*_2_CH_3_), 0.92 (t, *J* = 7.4 Hz, 3H, CH_2_CH_2_*CH*_3_). ^13^C NMR (63 MHz, CDCl_3_) *δ* 161.6 (C_q_), 150.2 (C_q_), 146.0 (C_6_), 56.3 (*CH*_2_CH_2_CH_3_), 54.0 (C_5_), 41.5 (CH_3_), 19.9 (CH_2_*CH*_2_CH_3_), 11.5 (CH_2_CH_2_*CH*_3_). IR (ATR diamond): *ν* (cm^−1^) 3105, 2941, 2810, 1498, 1420, 1324, 1136, 1021, 903, 777. HRMS (EI-MS) *m*/*z* calcd for C_8_H_12_IN_4_S: 323.0345 [M + H]^+^, found: 323.0346.

#### *N*-Cyclopropyl-5-iodoimidazo[1,2-*d*][1,2,4]thiadiazol-3-amine (18)

The reaction was carried out as described in general procedure B using 7 (170 mg, 0.94 mmol, 1.0 eq.), *N*-iodosuccinimide (233 mg, 1.04 mmol, 1.1 eq.) in dry DMF (8 mL). The crude mixture was purified by flash chromatography on silica gel (CH_2_Cl_2_/MeOH, 98/2) to afford 18 as a white solid (20 mg, 7%). *R*_f_ (CH_2_Cl_2_/MeOH, 95/5): 0.57. Mp: 174–176 °C. ^1^H NMR (400 MHz, CDCl_3_): *δ* (ppm) 7.28 (s, 1H, H_6_), 5.96 (s, 1H, NH), 2.92–2.78 (m, 1H, CH), 0.97–0.86 (m, 2H, CH_2_), 0.79–0.65 (m, 2H, CH_2_). ^13^C (101 MHz, CDCl_3_): *δ* (ppm) 162.3 (C_q_), 145.1 (C_6_), 144.3 (C_q_), 51.6 (C_5_), 24.5 (CH), 7.6 (2 × CH_2_). IR (ATR diamond): *ν* (cm^−1^) 3381, 1599, 1584, 1511, 1488, 1428, 1343, 1264, 1122, 952. HRMS (EI-MS) *m*/*z* calcd for C_7_H_8_IN_4_S: 306.9509 [M + H]^+^, found: 306.9509.

#### *N*-Cyclohexyl-5-iodoimidazo[1,2-*d*][1,2,4]thiadiazol-3-amine (19)

The reaction was carried out as described in general procedure B using 8 (190 mg, 0.85 mmol, 1.0 eq.), *N*-iodosuccinimide (212 mg, 0.94 mmol, 1.1 eq.) in dry DMF (7 mL). The crude mixture was purified by flash chromatography on silica gel (PE/EtOAc, 9/1) to afford 19 as a white solid (250 mg, 84%). *R*_f_ (PE/EtOAc, 9/1): 0.42. Mp: 104–106 °C. ^1^H NMR (400 MHz, CDCl_3_): *δ* (ppm) 7.28 (s, 1H, H_6_), 5.64 (d, *J* = 7.4 Hz, 1H, NH), 3.87 (m, 1H, CH), 2.21–2.04 (m, 2H, CH_2_), 1.86–1.69 (m, 2H, CH_2_), 1.69–1.30 (m, 6H, 3 × CH_2_). ^13^C NMR (101 MHz, CDCl_3_): *δ* (ppm) 162.2 (C_q_), 144.9 (C_6_), 143.0 (C_q_), 51.5 (C_5_), 51.4 (CH), 32.9 (2 × CH_2_), 25.7 (CH_2_), 24.5 (2 × CH_2_). IR (ATR diamond): *ν* (cm^−1^) 3379, 2927, 2850, 1578, 1489, 1449, 1434, 1273, 1122, 1098. HRMS (EI-MS) *m*/*z* calcd for C_10_H_14_IN_4_S: 348.9978 [M + H]^+^, found: 348.9980.

#### 5-Iodo-3-(piperidin-1-yl)imidazo[1,2-*d*][1,2,4]thiadiazole (20)

The reaction was carried out as described in general procedure B using 9 (100 mg, 0.48 mmol, 1.0 eq.), *N*-iodosuccinimide (119 mg, 0.53 mmol, 1.1 eq.) in dry DMF (5 mL). The crude mixture was purified by flash chromatography on silica gel (CH_2_Cl_2_/MeOH, 98/2) to afford 20 as a white solid (100 mg, 62%). *R*_f_ (CH_2_Cl_2_/MeOH, 95/5): 0.67. Mp: 108–110 °C. ^1^H NMR (400 MHz, CDCl_3_): *δ* (ppm) 7.35 (s, 1H, H_6_), 3.30–3.22 (m, 4H, 2 × CH_2_), 1.89–1.78 (m, 4H, 2 × CH_2_), 1.74–1.60 (m, 2H, CH_2_). ^13^C NMR (101 MHz, CDCl_3_): *δ* (ppm) 161.8 (C_q_), 150.7 (C_q_), 145.9 (C_6_), 53.9 (C_5_), 52.6 (2 × CH_2_), 24.9 (2 × CH_2_), 23.9 (CH_2_). IR (ATR diamond): *ν* (cm^−1^) 2931, 2849, 1549, 1483, 1428, 1282, 1262, 1204, 1130, 883. HRMS (EI-MS) *m*/*z* calcd for C_9_H_12_IN_4_S: 334.9822 [M + H]^+^, found: 334.9819.

#### 5-Iodo-3-(4-methylpiperazin-1-yl)imidazo[1,2-*d*][1,2,4]thiadiazole (21)

The reaction was carried out as described in general procedure B using 10 (240 mg, 1.07 mmol, 1.0 eq.), *N*-Iodosuccinimide (266 mg, 1.18 mmol, 1.1 eq.) in dry DMF (12 mL). The crude mixture was purified by flash chromatography on silica gel (CH_2_Cl_2_/MeOH, 98/2) to afford 21 as a white solid (60 mg, 16%). *R*_f_ (CH_2_Cl_2_/MeOH, 95/5): 0.40. Mp: 154–156 °C. ^1^H NMR (400 MHz, CDCl_3_): *δ* (ppm) 7.35 (s, 1H, H_6_), 3.39 (m, 4H, 2 × CH_2_), 2.71 (m, 4H, 2 × CH_2_), 2.39 (s, 3H, CH_3_). ^13^C NMR (101 MHz, CDCl_3_): *δ* (ppm) 162.0 (C_q_), 149.5 (C_q_), 146.4 (C_6_), 54.0 (2 × CH_2_), 53.7 (C_5_), 51.2 (2 × CH_2_), 46.3 (CH_3_). IR (ATR diamond): *ν* (cm^−1^) 2942, 2848, 2808, 1680, 1549, 1485, 1453, 1426, 1395, 1263, 1134, 1002. HRMS (EI-MS) *m*/*z* calcd for C_9_H_13_IN_5_S: 349.9931 [M + H]^+^, found: 349.9931.

#### 5-Iodo-3-(morpholin-4-yl)imidazo[1,2-*d*][1,2,4]thiadiazole (22)

The reaction was carried out as described in general procedure B using 11 (100 mg, 0.48 mmol, 1.0 eq.), *N*-iodosuccinimide (119 mg, 0.53 mmol, 1.1 eq.) in dry DMF (5 mL). The crude mixture was purified by flash chromatography on silica gel (CH_2_Cl_2_/MeOH, 98/2) to afford 22 as a pale yellow solid (148 mg, 92%). *R*_f_ (PE/EtOAc, 1/1): 0.75. Mp: 236–238 °C. ^1^H NMR (400 MHz, CDCl_3_): *δ* (ppm) 7.36 (s, 1H, H_6_), 4.01–3.82 (m, 4H, 2 × CH_2_), 3.45–3.25 (m, 4H, 2 × CH_2_). ^13^C NMR (101 MHz, CDCl_3_): *δ* (ppm) 161.9 (C_q_), 149.2 (C_q_), 146.4 (C_6_), 66.0 (2 × CH_2_), 53.6 (C_5_), 51.5 (2 × CH_2_). IR (ATR diamond): *ν* (cm^−1^) 3115, 2953, 2859, 1562, 1425, 1295, 1267, 1106, 1015, 892, 786. HRMS (EI-MS) *m*/*z* calcd for C_8_H_10_IN_4_OS: 336.9615 [M + H]^+^, found: 336.9615.

#### *N*-Benzyl-5-iodoimidazo[1,2-*d*][1,2,4]thiadiazol-3-amine (23)

The reaction was carried out as described in general procedure B using 12 (200 mg, 0.87 mmol, 1.0 eq.), *N*-iodosuccinimide (215 mg, 0.96 mmol, 1.1 eq.) in dry DMF (10 mL). The crude mixture was purified by flash chromatography on silica gel (PE/EtOAc, 9/1) to afford 23 as a yellow solid (184 mg, 59%). *R*_f_ (PE/EtOAc, 6/4): 0.59. Mp: 154–156 °C. ^1^H NMR (400 MHz, CDCl_3_): *δ* (ppm) 7.47–7.32 (m, 5H, 5 × H_Ar_), 7.30 (s, 1H, H_6_), 5.95 (s, 1H, NH), 4.71 (d, *J* = 5.5 Hz, 2H, CH_2_). ^13^C NMR (101 MHz, CDCl_3_): *δ* (ppm) 162.4 (C_q_), 145.0 (C_6_), 143.6 (C_q_), 137.4 (C_q_), 129.1 (2 × CH_Ar_), 128.2 (C_Ar_), 127.8 (2 × CH_Ar_), 51.7 (C_5_), 46.7 (CH_2_). IR (ATR diamond): *ν* (cm^−1^) 3382, 2922, 2868, 1605, 1506, 1489, 1434, 1280, 1127, 743, 691. HRMS (EI-MS) *m*/*z* calcd for C_11_H_10_IN_4_S: 356.9665 [M + H]^+^, found: 356.9663.

#### 5-Iodo-3-methoxyimidazo[1,2-*d*][1,2,4]thiadiazole (24)

The reaction was carried out as described in general procedure B using 14 (150 mg, 0.97 mmol, 1.0 eq.), *N*-iodosuccinimide (240 mg, 1.07 mmol, 1.1 eq.) in dry DMF (5 mL). The crude mixture was purified by flash chromatography on silica gel (PE/EtOAc, 7/3) to afford 24 as an off-white solid (227 mg, 83%). *R*_f_ (PE/EtOAc, 1/1): 0.74. Mp: 229–231 °C. ^1^H NMR (400 MHz, CDCl_3_): *δ* (ppm) 7.28 (s, 1H, H_6_), 4.22 (s, 3H, CH_3_). ^13^C NMR (101 MHz, CDCl_3_): *δ* (ppm) 160.9 (C_q_), 146.6 (C_q_), 145.3 (C_6_), 57.6 (CH_3_), 53.3 (C_5_). IR (ATR diamond): *ν* (cm^−1^) 3100, 2945, 1589, 1448, 1397, 1289, 1118, 968, 851, 698. HRMS (EI-MS) *m*/*z* calcd for C_5_H_5_IN_3_OS: 281.9193 [M + H]^+^, found: 281.9191.

#### 5-Iodo-3-ethoxyimidazo[1,2-*d*][1,2,4]thiadiazole (25)

The reaction was carried out as described in general procedure B using 15 (160 mg, 0.95 mmol, 1.0 eq.), *N*-Iodosuccinimide (235 mg, 1.05 mmol, 1.1 eq.) in dry DMF (8 mL). The crude mixture was purified by flash chromatography on silica gel (PE/EtOAc, 7/3) to afford 25 as a white solid (250 mg, 89%). *R*_f_ (PE/EtOAc, 1/1): 0.74. Mp: 228–230 °C. ^1^H NMR (250 MHz, CDCl_3_): *δ* (ppm) 7.28 (s, 1H), 4.62 (q, *J* = 7.1 Hz, 2H), 1.54 (t, *J* = 7.1 Hz, 3H). ^13^C NMR (63 MHz, CDCl_3_): *δ* (ppm) 160.6 (C_q_), 146.0 (C_q_), 145.2 (C_6_), 67.3 (CH_2_), 53.4 (C_5_), 14.4 (CH_3_). IR (ATR diamond): *ν* (cm^−1^) 3124, 2985, 1592, 1441, 1381, 1274, 1110, 958, 843, 703. HRMS (EI-MS) *m*/*z* calcd for C_6_H_7_IN_3_OS: 295.9512 [M + H]^+^, found: 295.9511.

#### 5-Bromo-3-(morpholin-4-yl)imidazo[1,2-*d*][1,2,4]thiadiazole (26)

The reaction was carried out as described in general procedure B using 11 (40 mg, 0.20 mmol, 1.0 eq.), *N*-bromosuccinimide (39 mg, 0.22 mmol, 1.1 eq.) in dry DMF (2 mL). The crude mixture was purified by flash chromatography on silica gel (PE/EtOAc, 5/5) to afford 26 as a white solid (20 mg, 34%). *R*_f_ (PE/EtOAc, 5/5): 0.57. Mp: 159–161 °C. ^1^H NMR (400 MHz, DMSO): *δ* (ppm) 7.43 (s, 1H, H_6_), 3.79 (t, *J* = 4.8 Hz, 4H, 2 × CH_2_), 3.29 (t, *J* = 4.7 Hz, 4H, 2 × CH_2_). ^13^C NMR (101 MHz, DMSO): *δ* (ppm) 158.7 (C_q_), 148.6 (C_q_), 138.6 (C_6_), 93.6 (C_q_), 65.2 (2 × CH_2_), 50.8 (2 × CH_2_). IR (ATR diamond): *ν* (cm^−1^) 2925, 2852, 1562, 1435, 1266, 1110, 1019, 894, 838, 786. HRMS (EI-MS) *m*/*z* calcd for C_8_H_10_BrN_4_OS: 288.9753 [M + H]^+^, found: 288.9754.

#### 5-Bromo-3-methoxy-imidazo[1,2-*d*][1,2,4]thiadiazole (27)

The reaction was carried out as described in general procedure B using 14 (190 mg, 1.22 mmol, 1.0 eq.), *N*-Bromosuccinimide (239 mg, 1.35 mmol, 1.1 eq.) in dry DMF (5 mL). The crude mixture was purified by flash chromatography on silica gel (PE/EtOAc, 6/4) to afford 27 as a beige solid (210 mg, 73%). *R*_f_ (PE/EtOAc, 5/5): 0.61 Mp: 137–139 °C. ^1^H NMR (250 MHz, CDCl_3_) *δ* (ppm) 7.19 (s, 1H, H_6_), 4.21 (s, 3H, OCH_3_). ^13^C NMR (63 MHz, CDCl_3_) *δ* 158.9 (C_q_), 146.4 (C_q_), 138.2 (C_6_), 93.1 (C_q_), 57.7 (OCH_3_). IR (ATR diamond): *ν* (cm^−1^) 2810, 2450, 1620, 1400, 1102, 1110, 1005, 850, 808, 775. HRMS (EI-MS) *m*/*z* calcd for C_5_H_5_BrN_3_OS: 232.9312 [M + H]^+^, found: 232.9325.

#### 4-(5-(*p*-Tolyl)imidazo[1,2-*d*][1,2,4]thiadiazol-3-yl)morpholine (28)

The reaction was carried out as described in general procedure C using 22 (50 mg, 0.15 mmol, 1.0 eq.), cesium carbonate (98 mg, 0.30 mmol, 2.0 eq.), *p*-tolylboronic acid (25 mg, 0.18 mmol, 1.2 eq.) in degassed dioxane (1.5 mL). The crude mixture was purified by flash chromatography on silica gel (PE/EtOAc, 6/4) to afford 28 as a white solid (27 mg, 60%). *R*_f_ (PE/EtOAc, 1/1): 0.41. Mp: 188–190 °C. ^1^H NMR (400 MHz, CDCl_3_): *δ* (ppm) 7.43 (d, *J* = 7.7 Hz, 2H, 2 × H_Ar_), 7.28 (d, *J* = 4.5 Hz, 2H, 2 × H_Ar_), 7.26 (s, 1H H_6_), 3.53–3.45 (m, 4H, 2 × CH_2_), 3.06–2.97 (m, 4H, 2 × CH_2_), 2.42 (s, 3H, CH_3_). ^13^C NMR (101 MHz, CDCl_3_): *δ* (ppm) 149.0 (C_q_), 138.6 (C_q_), 136.7 (C_6_), 129.3 (2 × CH_Ar_), 129.2 (2 × CH_Ar_), 125.9 (C_q_), 65.6 (2 × CH_2_), 50.2 (2 × CH_2_), 21.4 (CH_3_). IR (ATR diamond): *ν* (cm^−1^) 2954, 2861, 1541, 1457, 1439, 1390, 1274, 1252, 1113, 811, 719. HRMS (EI-MS) *m*/*z* calcd for C_15_H_17_N_4_OS: 301.1118 [M + H]^+^, found: 301.1117.

#### 4-(5-Phenylimidazo[1,2-*d*][1,2,4]thiadiazol-3-yl)morpholine (29)

The reaction was carried out as described in general procedure C using 22 (50 mg, 0.15 mmol, 1.0 eq.), cesium carbonate (98 mg, 0.30 mmol, 2.0 eq.), phenylboronic acid (22 mg, 0.18 mmol, 1.2 eq.) in degassed dioxane (1.5 mL). The crude mixture was purified by flash chromatography on silica gel (PE/EtOAc, 6/4) to afford 29 as a white solid (31 mg, 72%). *R*_f_ (PE/EtOAc, 1/1): 0.39. Mp: 183–185 °C. ^1^H NMR (400 MHz, CDCl_3_): *δ* (ppm) 7.54 (d, *J* = 7.4 Hz, 2H, 2 × H_Ar_), 7.49–7.37 (m, 3H, 3 × H_Ar_), 7.32 (s, 1H, H_6_), 3.49–3.39 (m, 4H, 2 × CH_2_), 3.05–2.98 (m, 4H, 2 × CH_2_). ^13^C NMR (101 MHz, CDCl_3_): *δ* (ppm) 160.3 (C_q_), 149.0 (C_q_), 137.0 (C_6_), 129.2 (2 × CH_Ar_), 128.8 (C_q_), 128.6 (2 × CH_Ar_), 128.5 (CH_Ar_), 128.2 (C_q_), 65.5 (2 × CH_2_), 50.1 (2 × CH_2_). IR (ATR diamond): *ν* (cm^−1^) 2952, 2919, 1563, 1537, 1453, 1369, 1273, 1256, 1113, 844, 764. HRMS (EI-MS) *m*/*z* calcd for C_14_H_15_N_4_OS: 287.0961 [M + H]^+^, found: 287.0961.

#### 4-(5-(4-Methoxyphenyl)imidazo[1,2-*d*][1,2,4]thiadiazol-3-yl)morpholine (30)

The reaction was carried out as described in general procedure C using 22 (50 mg, 0.15 mmol, 1.0 eq.), cesium carbonate (98 mg, 0.30 mmol, 2.0 eq.), 4-methoxyphenylboronic acid (27 mg, 0.18 mmol, 1.2 eq.) in degassed dioxane (1.5 mL). The crude mixture was purified by flash chromatography on silica gel (PE/EtOAc, 1/1) to afford 30 as a white solid (31 mg, 65%). *R*_f_ (PE/EtOAc, 1/1): 0.31. Mp: 169–171 °C. ^1^H NMR (400 MHz, CDCl_3_): *δ* (ppm) 7.46 (d, *J* = 8.4 Hz, 2H, 2 × H_Ar_), 7.25 (s, 1H, H_6_), 6.98 (d, *J* = 8.3 Hz, 2H, 2 × H_Ar_), 3.86 (s, 3H, CH_3_), 3.53–3.44 (m, 4H, 2 × CH_2_), 3.06–2.99 (m, 4H, 2 × CH_2_). ^13^C NMR (101 MHz, CDCl_3_): *δ* (ppm) 160.0 (C_q_), 159.7 (C_q_), 149.0 (C_q_), 136.5 (C_6_), 130.7 (2 × CH_Ar_), 128.0 (C_q_), 121.2 (C_q_), 114.0 (2 × CH_Ar_), 65.6 (2 × CH_2_), 55.6 (CH_3_), 50.2 (2 × CH_2_). IR (ATR diamond): *ν* (cm^−1^) 2953, 2853, 1543, 1498, 1446, 1261, 1244, 1113, 820, 719. HRMS (EI-MS) *m*/*z* calcd for C_15_H_17_N_4_O_2_S: 317.1067 [M + H]^+^, found: 317.1067.

#### 4-(5-(3-Methoxyphenyl)imidazo[1,2-*d*][1,2,4]thiadiazol-3-yl)morpholine (31)

The reaction was carried out as described in general procedure C using 22 (50 mg, 0.15 mmol, 1.0 eq.), cesium carbonate (98 mg, 0.30 mmol, 2.0 eq.), 3-methoxyphenylboronic acid (27 mg, 0.18 mmol, 1.2 eq.) in degassed dioxane (1.5 mL). The crude mixture was purified by flash chromatography on silica gel (PE/EtOAc, 1/1) to afford 31 as a white solid (24 mg, 50%). *R*_f_ (PE/EtOAc, 1/1): 0.30. Mp: 150–152 °C. ^1^H NMR (250 MHz, CDCl_3_): *δ* (ppm) 7.42–7.31 (m, 2H, 2 × H_Ar_), 7.15–7.05 (m, 2H, 2 × H_Ar_), 7.00–6.88 (m, 1H, H_Ar_), 3.87 (s, 3H), 3.54–3.46 (m, 4H, 2 × CH_2_), 3.07–2.99 (m, 4H, 2 × CH_2_). ^13^C NMR (63 MHz, CDCl_3_): *δ* (ppm) 160.4 (C_q_), 159.5 (C_q_), 149.0 (C_q_), 137.1 (C_6_), 130.0 (C_q_), 129.7 (CH_Ar_), 128.1 (C_q_), 121.4 (CH_Ar_), 115.0 (CH_Ar_), 113.8 (CH_Ar_), 65.6 (2 × CH_2_), 55.5 (CH_3_), 50.2 (2 × CH_2_). IR (ATR diamond): *ν* (cm^−1^) 2898, 2850, 1568, 1541, 1463, 1448, 1292, 1260, 1110, 845, 786. HRMS (EI-MS) *m*/*z* calcd for C_15_H_17_N_4_O_2_S: 317.1067 [M + H]^+^, found: 317.1071.

#### 4-(5-(2-Methoxyphenyl)imidazo[1,2-*d*][1,2,4]thiadiazol-3-yl)morpholine (32)

The reaction was carried out as described in general procedure C using 22 (50 mg, 0.15 mmol, 1.0 eq.), cesium carbonate (98 mg, 0.30 mmol, 2.0 eq.), 2-methoxyphenylboronic acid (27 mg, 0.18 mmol, 1.2 eq.) in degassed dioxane (1.5 mL). The crude mixture was purified by flash chromatography on silica gel (PE/EtOAc, 1/1) to afford 32 as a white solid (16 mg, 34%). *R*_f_ (PE/EtOAc, 1/1): 0.31. Mp: 148–150 °C. ^1^H NMR (250 MHz, CDCl_3_): *δ* (ppm) 7.48–7.28 (m, 3H, 3 × H_Ar_), 7.08–6.92 (m, 2H, 2 × H_Ar_), 3.79 (s, 3H, CH_3_), 3.35–3.27 (m, 4H, 2 × CH_2_), 3.01–2.93 (m, 4H, 2 × CH_2_). ^13^C NMR (63 MHz, CDCl_3_): *δ* (ppm) 159.7 (C_q_), 157.9 (C_q_), 149.4 (C_q_), 137.5 (C_6_), 132.0 (CH_Ar_), 130.7 (CH_Ar_), 123.6 (C_q_), 120.5 (CH_Ar_), 118.0 (C_q_), 110.9 (CH_Ar_), 65.7 (2 × CH_2_), 55.4 (CH_3_), 50.2 (2 × CH_2_). IR (ATR diamond): *ν* (cm^−1^) 2972, 2849, 1542, 1487, 1401, 1283, 1262, 1115, 840, 758. HRMS (EI-MS) *m*/*z* calcd for C_15_H_17_N_4_O_2_S: 317.1067 [M + H]^+^, found: 317.1071.

#### 4-(3-Morpholinoimidazo[1,2-*d*][1,2,4]thiadiazol-5-yl)phenol (33)

The reaction was carried out as described in general procedure C using 22 (50 mg, 0.15 mmol, 1.0 eq.), cesium carbonate (98 mg, 0.30 mmol, 2.0 eq.), 4-(tetrahydro-2*H*-pyran-2-yloxy)phenylboronic acid (40 mg, 0.18 mmol, 1.2 eq.) in degassed dioxane (1.5 mL). The crude mixture was purified by flash chromatography on silica gel (PE/EtOAc, 6/4) to afford the –OTHP protected derivative. The product was dissolved in a mixture of AcOH/THF/Water (4 : 2 : 1, 2 mL) and heated at 45 °C for 12 h. The reaction was then concentrated and the residue was taken up in CH_2_Cl_2_ (5 mL). The product was precipitated by addition of pentane (10 mL) and filtered to afford 33 (27 mg, 59%, over 2 steps) as a white solid. *R*_f_ (PE/EtOAc, 1/1): 0.21. Mp >250 °C. ^1^H NMR (400 MHz, DMSO*-d*_6_): *δ* (ppm) 9.75 (s, 1H, OH), 7.40 (d, *J* = 8.5 Hz, 2H, 2 × H_Ar_), 7.28 (s, 1H, H_6_), 6.89 (d, *J* = 8.5 Hz, 2H, 2 × H_Ar_), 3.51–3.27 (m, 4H, 2 × CH_2_), 2.96–2.85 (m, 4H, 2 × CH_2_). ^13^C 135DEPT NMR (101 MHz, DMSO*-d*_6_): *δ* (ppm) 135.6 (C_6_), 130.5 (2 × CH_Ar_), 115.2 (2 × CH_Ar_), 64.7 (2 × CH_2_), 49.7 (2 × CH_2_). IR (ATR diamond): *ν* (cm^−1^) 2916, 2855, 1544, 1450, 1369, 1278, 1258, 1111, 831, 798. HRMS (EI-MS) *m*/*z* calcd for C_14_H_15_N_4_O_2_S: 303.0910 [M + H]^+^, found: 303.0912.

#### 4-(5-(4-Nitrophenyl)imidazo[1,2-*d*][1,2,4]thiadiazol-3-yl)morpholine (34)

The reaction was carried out as described in general procedure C using 22 (50 mg, 0.15 mmol, 1.0 eq.), cesium carbonate (98 mg, 0.30 mmol, 2.0 eq.), 4-nitrophenylboronic acid (30 mg, 0.18 mmol, 1.2 eq.) in degassed dioxane (1.5 mL). The crude mixture was purified by flash chromatography on silica gel (PE/EtOAc, 1/1) to afford 34 as a yellow solid (20 mg, 40%). *R*_f_ (PE/EtOAc, 1/1): 0.28. Mp >250 °C. ^1^H NMR (250 MHz, CDCl_3_): *δ* (ppm) 8.34 (d, *J* = 8.8 Hz, 2H, 2 × H_Ar_), 7.77 (d, *J* = 8.8 Hz, 2H, 2 × H_Ar_), 7.51 (s, 1H, H_Ar_), 3.60–3.52 (m, 4H, 2 × CH_2_), 3.10–3.04 (m, 4H, 2 × CH_2_). ^13^C 135DEPT NMR (63 MHz, CDCl_3_): *δ* (ppm) 128.9 (2 × CH_Ar_), 124.1 (2 × CH_Ar_), 65.6 (2 × CH_2_), 50.1 (2 × CH_2_). IR (ATR diamond): *ν* (cm^−1^) 2924, 2863, 1591, 1508, 1437, 1339, 1277, 1256, 1110, 846, 747. HRMS (EI-MS) *m*/*z* calcd for C_14_H_14_N_5_O_3_S: 332.0812 [M + H]^+^, found: 332.0813.

#### 4-(5-(4-Fluorophenyl)imidazo[1,2-*d*][1,2,4]thiadiazol-3-yl)morpholine (35)

The reaction was carried out as described in general procedure C using 22 (50 mg, 0.15 mmol, 1.0 eq.), cesium carbonate (98 mg, 0.30 mmol, 2.0 eq.), 4-fluorophenylboronic acid (25 mg, 0.18 mmol, 1.2 eq.) in degassed dioxane (1.5 mL). The crude mixture was purified by flash chromatography on silica gel (PE/EtOAc, 6/4) to afford 35 as a white solid (17 mg, 38%). *R*_f_ (PE/EtOAc, 1/1): 0.38. Mp = 149–151 °C. ^1^H NMR (400 MHz, CDCl_3_): *δ* (ppm) 7.56–7.49 (m, 2H, 2 × H_Ar_), 7.30 (s, 1H, H_6_), 7.16 (t, *J* = 8.6 Hz, 2H, 2 × H_Ar_), 3.52–3.45 (m, 4H, 2 × CH_2_), 3.06–2.99 (m, 4H, 2 × CH_2_). ^13^C NMR (101 MHz, CDCl_3_): *δ* (ppm) 162.9 (d, *J* = 249.5 Hz, C_q_F), 160.4 (C_q_), 148.9 (C_q_), 137.1 (C_6_), 131.0 (d, *J* = 8.1 Hz, 2 × CH_Ar_), 127.0 (C_q_), 124.9 (d, *J* = 3.4 Hz, C_q_), 115.7 (d, *J* = 21.7 Hz, 2 × CH_Ar_), 65.6 (2 × CH_2_), 50.2 (2 × CH_2_). ^19^F NMR (376 MHz, CDCl_3_): *δ* (ppm) −112.81. IR (ATR diamond): *ν* (cm^−1^)2961, 2854, 1541, 1495, 1459, 1387, 1276, 1256, 1215, 1118, 831, 719. HRMS (EI-MS) *m*/*z* calcd for C_14_H_14_FN_4_OS: 305.0867 [M + H]^+^, found: 305.0869.

#### 4-[5-(3-Thienyl)imidazo[1,2-*d*][1,2,4]thiadiazol-3-yl]morpholine (36)

The reaction was carried out as described in general procedure C using 22 (50 mg, 0.15 mmol, 1.0 eq.), cesium carbonate (98 mg, 0.30 mmol, 2.0 eq.), 3-thiopheneboronic acid (23 mg, 0.18 mmol, 1.2 eq.) in degassed dioxane (1.5 mL). The crude mixture was purified by flash chromatography on silica gel (PE/EtOAc, 1/1) to afford 36 as a white solid (31 mg, 71%). *R*_f_ (PE/EtOAc, 1/1): 0.38. Mp = 177–179 °C. ^1^H NMR (250 MHz, CDCl_3_): *δ* (ppm) 7.54–7.19 (m, 4H, 4 × H_Ar_), 3.67–3.48 (m, 4H, 2 × CH_2_), 3.20–2.92 (m, 4H, 2 × CH_2_). ^13^C NMR (63 MHz, CDCl_3_): *δ* (ppm) 159.9 (C_q_), 149.1 (C_q_), 137.0 (C_6_), 128.9 (C_q_), 128.8 (CH_Ar_), 126.1 (CH_Ar_), 124.1 (CH_Ar_), 123.1 (C_q_), 65.6 (2 × CH_2_), 50.4 (2 × CH_2_). IR (ATR diamond): *ν* (cm^−1^) 3117, 2958, 2859, 1557, 1447, 1269, 1246, 1112, 842, 789. HRMS (EI-MS) *m*/*z* calcd for C_12_H_13_N_4_OS_2_: 293.0525 [M + H]^+^, found: 293.0529.

#### *N*-Methyl-*N*-propyl-5-(*p*-tolyl)imidazo[1,2-*d*][1,2,4]thiadiazol-3-amine (40)

The reaction was carried out as described in general procedure C using 17 (260 mg, 0.80 mmol, 1.0 eq.), cesium carbonate (520 mg, 1.60 mmol, 2.0 eq.), *p*-tolylboronic acid (130 mg, 0.96 mmol, 1.2 eq.) in degassed dioxane (4 mL). The crude mixture was purified by flash chromatography on silica gel (PE/EtOAc, 7/3) to afford 40 as an amorphous solid (163 mg, 71%). *R*_f_ (PE/EtOAc, 7/3): 0.42. ^1^H NMR (400 MHz, CDCl_3_): *δ* (ppm) 7.41 (d, *J* = 8.1 Hz, 2H, 2 × H_Ar_), 7.27 (s, 1H, H_6_), 7.22 (d, *J* = 7.8 Hz, 2H, 2 × H_Ar_), 3.01–2.89 (m, 2H, *CH*_2_CH_2_CH_3_), 2.49 (s, 3H, CH_3_), 2.40 (s, 3H, CH_3_), 1.45 (h, *J* = 7.4 Hz, 2H, CH_2_*CH*_2_CH_3_), 0.77 (t, *J* = 7.4 Hz, 3H, CH_2_CH_2_*CH*_3_). ^13^C NMR (101 MHz, CDCl_3_): *δ* (ppm) 159.9 (C_q_), 149.8 (C_q_), 138.0 (C_q_), 136.4 (C_6_), 129.0 (2 × CH_Ar_), 128.8 (2 × CH_Ar_), 128.5 (C_q_), 126.2 (C_q_), 55.0 (*CH*_2_CH_2_CH_3_), 38.9 (CH_3_), 21.3 (CH_3_), 19.7 (CH_2_*CH*_2_CH_3_), 11.4 (CH_2_CH_2_*CH*_3_). IR (ATR diamond): *ν* (cm^−1^)2964, 2873, 1541, 1456, 1389, 1251, 1140, 815, 717. HRMS (EI-MS) *m*/*z* calcd for C_15_H_19_N_4_S: 287.1325 [M + H]^+^, found: 287.1326.

#### 3-(Piperidin-1-yl)-5-(*p*-tolyl)imidazo[1,2-*d*][1,2,4]thiadiazole (43)

The reaction was carried out as described in general procedure C using 20 (50 mg, 0.15 mmol, 1.0 eq.), cesium carbonate (98 mg, 0.30 mmol, 2.0 eq.), *p*-tolylboronic acid (25 mg, 0.18 mmol, 1.2 eq.) in degassed dioxane (1.5 mL). The crude mixture was purified by flash chromatography on silica gel (CH_2_Cl_2_/MeOH, 97/3) to afford 43 as a beige solid (31 mg, 70%). *R*_f_ (CH_2_Cl_2_/MeOH, 95/5): 0.38. Mp: 114–116 °C. ^1^H NMR (400 MHz, CDCl_3_): *δ* (ppm) 7.43 (d, *J* = 8.0 Hz, 2 × H_Ar_), 7.27 (s, 1H, H_6_), 7.25 (d, *J* = 8.0 Hz, 2 × H_Ar_), 2.98 (m, 4H, 2 × CH_2_), 2.41 (s, 3H, CH_3_), 1.47–1.42 (m, 2H, CH_2_), 1.36–1.30 (m, 4H, 2 × CH_2_). ^13^C NMR (101 MHz, CDCl_3_): *δ* (ppm) 160.0 (C_q_), 150.2 (C_q_), 138.1 (C_q_), 136.7 (C_6_), 129.1 (2 × CH_Ar_), 129.0 (2 × CH_Ar_), 126.1 (C_q_), 100.1 (C_q_), 51.0 (2 × CH_2_), 24.4 (2 × CH_2_), 23.9 (CH_2_), 21.4 (CH_3_). IR (ATR diamond): *ν* (cm^−1^) 2917, 2849, 1538, 1449, 1438, 1401, 1280, 1245, 1165, 810. HRMS (EI-MS) *m*/*z* calcd for C_16_H_19_N_4_S: 299.1325 [M + H]^+^, found: 299.1323.

#### 3-(4-Methylpiperazin-1-yl)-5-(*p*-tolyl)imidazo[1,2-*d*][1,2,4]thiadiazole (44)

The reaction was carried out as described in general procedure C using 21 (40 mg, 0.11 mmol, 1.0 eq.), cesium carbonate (75 mg, 0.23 mmol, 2.0 eq.), *p*-tolylboronic acid (19 mg, 0.14 mmol, 1.2 eq.) in degassed dioxane (1.5 mL). The crude mixture was purified by flash chromatography on silica gel (CH_2_Cl_2_/MeOH, 95/5) to afford 44 as a white solid (20 mg, 56%). *R*_f_ (CH_2_Cl_2_/MeOH, 95/5): 0.47. Mp: 168–170 °C. ^1^H NMR (400 MHz, CDCl_3_): *δ* (ppm) 7.41 (d, *J* = 8.2 Hz, 2H, 2 × H_Ar_), 7.27 (s, 1H, H_6_), 7.24 (d, *J* = 7.9 Hz, 2H, 2 × H_Ar_), 3.06 (t, *J* = 4.9 Hz, 4H, 2 × CH_2_), 2.42 (s, 3H, CH_3_), 2.25–2.17 (m, 7H, 2 × CH_2_ + CH_3_). ^13^C NMR (101 MHz, CDCl_3_): *δ* (ppm) 160.0 (C_q_), 149.3 (C_q_), 138.3 (C_q_), 136.7 (C_6_), 129.3 (2 × CH_Ar_), 129.1 (2 × CH_Ar_), 128.4 (C_q_), 126.0 (C_q_), 53.6 (2 × CH_2_), 49.8 (2 × CH_2_), 46.2 (CH_3_), 21.4 (CH_3_). IR (ATR diamond): *ν* (cm^−1^) 2846, 2798, 1563, 1544, 1448, 1442, 1395, 1372, 1288, 1272, 1145, 1005, 814. HRMS (EI-MS) *m*/*z* calcd for C_16_H_20_N_5_S: 314.1434 [M + H]^+^, found: 314.1434.

#### 3-Methoxy-5-(*p*-tolyl)imidazo[1,2-*d*][1,2,4]thiadiazole (46)

The reaction was carried out as described in general procedure C using 24 (100 mg, 0.35 mmol, 1.0 eq.), cesium carbonate (228 mg, 0.70 mmol, 2.0 eq.), *p*-tolylboronic acid (68 mg, 0.50 mmol, 1.2 eq.) in degassed dioxane/water mixture (9/1, 2 mL). The crude mixture was purified by flash chromatography on silica gel (PE/EtOAc, 7/3) to afford 46 as a white solid (31 mg, 36%). *R*_f_ (PE/EtOAc, 1/1): 0.59. Mp: 170–172 °C. ^1^H NMR (400 MHz, CDCl_3_): *δ* (ppm) 7.43–7.38 (m, 2H, 2 × H_Ar_), 7.31–7.21 (m, 3H, 2 × H_Ar_ + H_6_), 4.15 (s, 3H, OCH_3_), 2.43 (s, 3H, CH_3_). ^13^C NMR (101 MHz, CDCl_3_): *δ* (ppm) 158.8 (C_q_), 147.3 (C_q_), 138.2 (C_q_), 135.5 (C_6_), 129.1 (2 × CH_Ar_), 128.8 (2 × CH_Ar_), 128.2 (C_q_), 125.0 (C_q_), 57.5 (OCH_3_), 21.4 (CH_3_). IR (ATR diamond): *ν* (cm^−1^) 2953, 1600, 1544, 1499, 1458, 1393, 1253, 1137, 961, 814. HRMS (EI-MS) *m*/*z* calcd for C_12_H_12_N_3_OS: 246.0696 [M + H]^+^, found: 246.0694.

#### 3-Ethoxy-5-(*p*-tolyl)imidazo[1,2-*d*][1,2,4]thiadiazole (47)

The reaction was carried out as described in general procedure C using 25 (150 mg, 0.51 mmol, 1.0 eq.), cesium carbonate (392 mg, 1.02 mmol, 2.0 eq.), *p*-tolylboronic acid (83 mg, 0.61 mmol, 1.2 eq.) in degassed dioxane/water mixture (9/1 3 mL). The crude mixture was purified by flash chromatography on silica gel (PE/EtOAc 9/1) to afford 47 as a white solid (80 mg, 60%). *R*_f_ (PE/EtOAc, 9/1): 0.24. Mp: 156–158 °C. ^1^H NMR (400 MHz, CDCl_3_): *δ* (ppm) 7.41 (d, *J* = 8.1 Hz, 2H, 2 × H_Ar_), 7.28 (s, 1H, H_6_), 7.21 (d, *J* = 8.2 Hz, 2H, 2 × H_Ar_), 4.55 (q, *J* = 7.1 Hz, 2H, O*CH*_2_CH_3_), 2.40 (s, 3H), 1.40 (t, *J* = 7.1 Hz, 3H, OCH_2_*CH*_3_). ^13^C NMR (101 MHz, CDCl_3_): *δ* (ppm) 158.7 (C_q_), 146.8 (C_q_), 138.1 (C_q_), 135.5 (C_6_), 129.0 (2 × CH_Ar_), 128.8 (2 × CH_Ar_), 128.6 (C_q_), 125.0 (C_q_), 67.1 (O*CH*_2_CH_3_), 21.4 (CH_3_), 14.3 (OCH_2_*CH*_3_). IR (ATR diamond): *ν* (cm^−1^) 2982, 2920, 1597, 1472, 1456, 1425, 1318, 1133, 1048, 821. HRMS (EI-MS) *m*/*z* calcd for C_13_H_14_N_3_OS: 260.0852 [M + H]^+^, found: 260.0854.

## Conflicts of interest

The authors declare no conflict of interest.

## Supplementary Material

RA-012-D1RA07208K-s001
