# Supplementary material for: Synthesis of novel series of 3,5-disubstituted imidazo[1,2-d] [1,2,4]thiadiazoles involving SNAr and Suzuki–Miyaura cross-coupling reactions
Source: RSC Adv. 2022 Feb 23;12(10):6303–13. doi: 10.1039/d1ra07208k (PMC8981913; doi:10.1039/d1ra07208k)

Electronic Supplementary Material (ESI) for RSC Advances.
This journal is © The Royal Society of Chemistry 2022

Synthesis of novel series of 3,5-disubstituted imidazo[1,2-*d*][1,2,4]thiadiazoles involving S_NAr and Suzuki-Miyaura cross-coupling reactions

Clémentine Pescheteau[a,†], Matthieu Place[a,†], Alexandru Sava[a,b], Léa Nunes[a], Lenuta Profire[b], Sylvain Routier[a,*] and Frédéric Buron[a,*]

[a] Institut de Chimie Organique et Analytique, ICOA, UMR CNRS 7311, Université d'Orléans, Orléans, France

[b] Department of Pharmaceutical Chemistry, Faculty of Pharmacy, "Grigore T. Popa" University of Medicine and Pharmacy of Iași, 16 University Street, Iasi, Romania.

† These authors equally contributed to the presented work.

The supporting information contains the ^{1}H NMR and ^{13}C NMR copy spectra.

***N*-propylimidazo[1,2-*d*][1,2,4]thiadiazol-3-amine (5)**

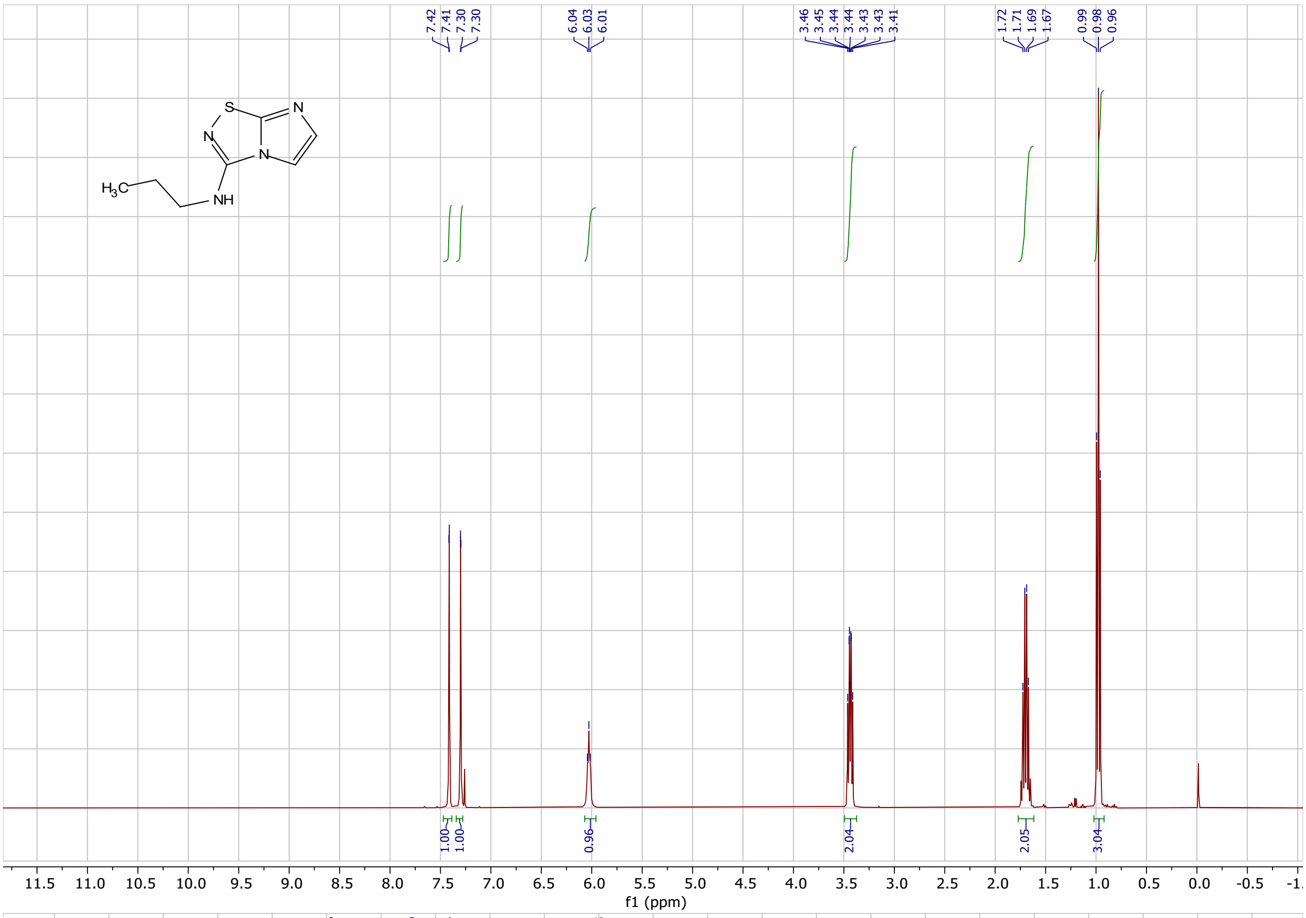

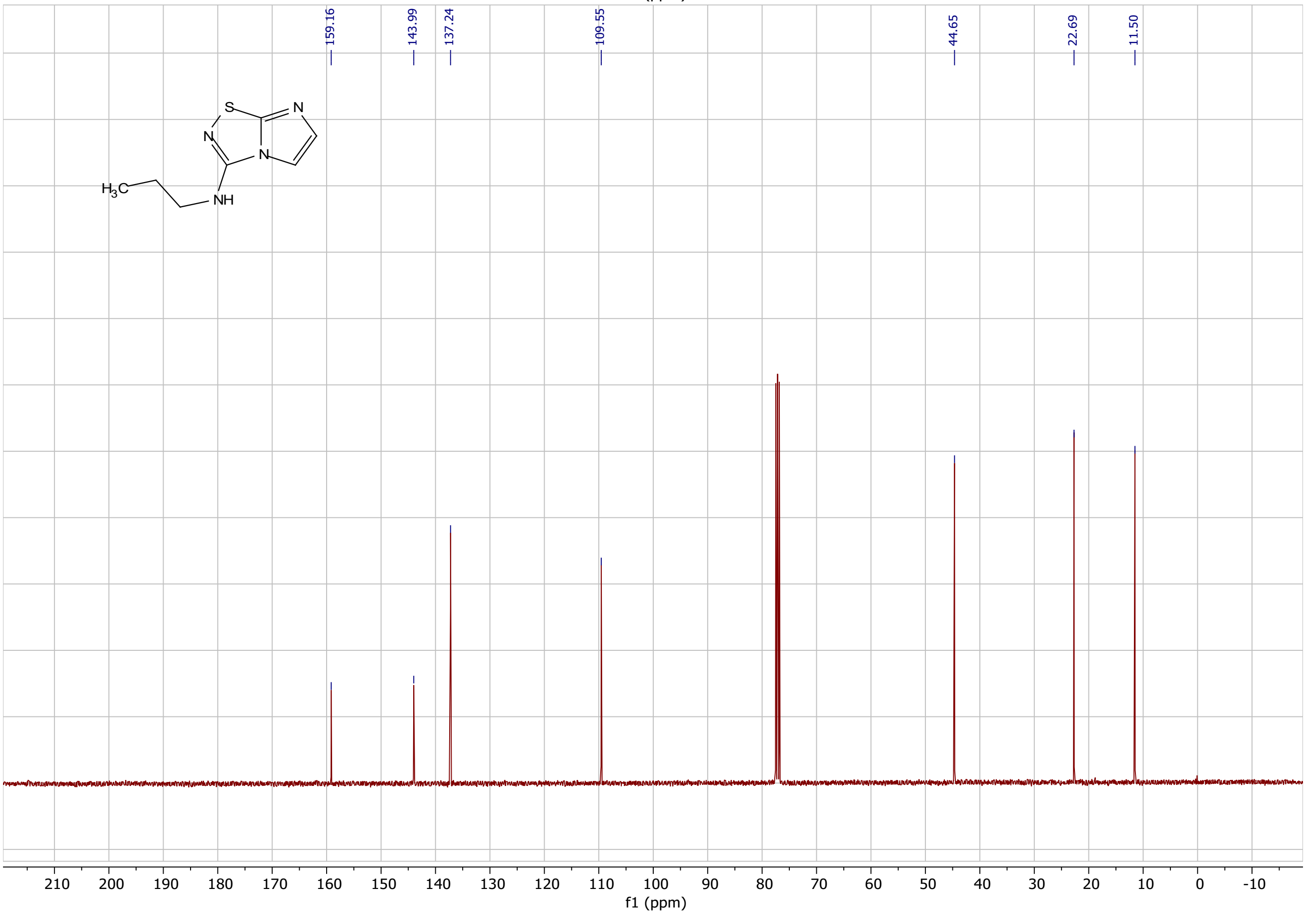

N-methyl-*N*-propylimidazo[1,2-*d*][1,2,4]thiadiazol-3-amine (6)

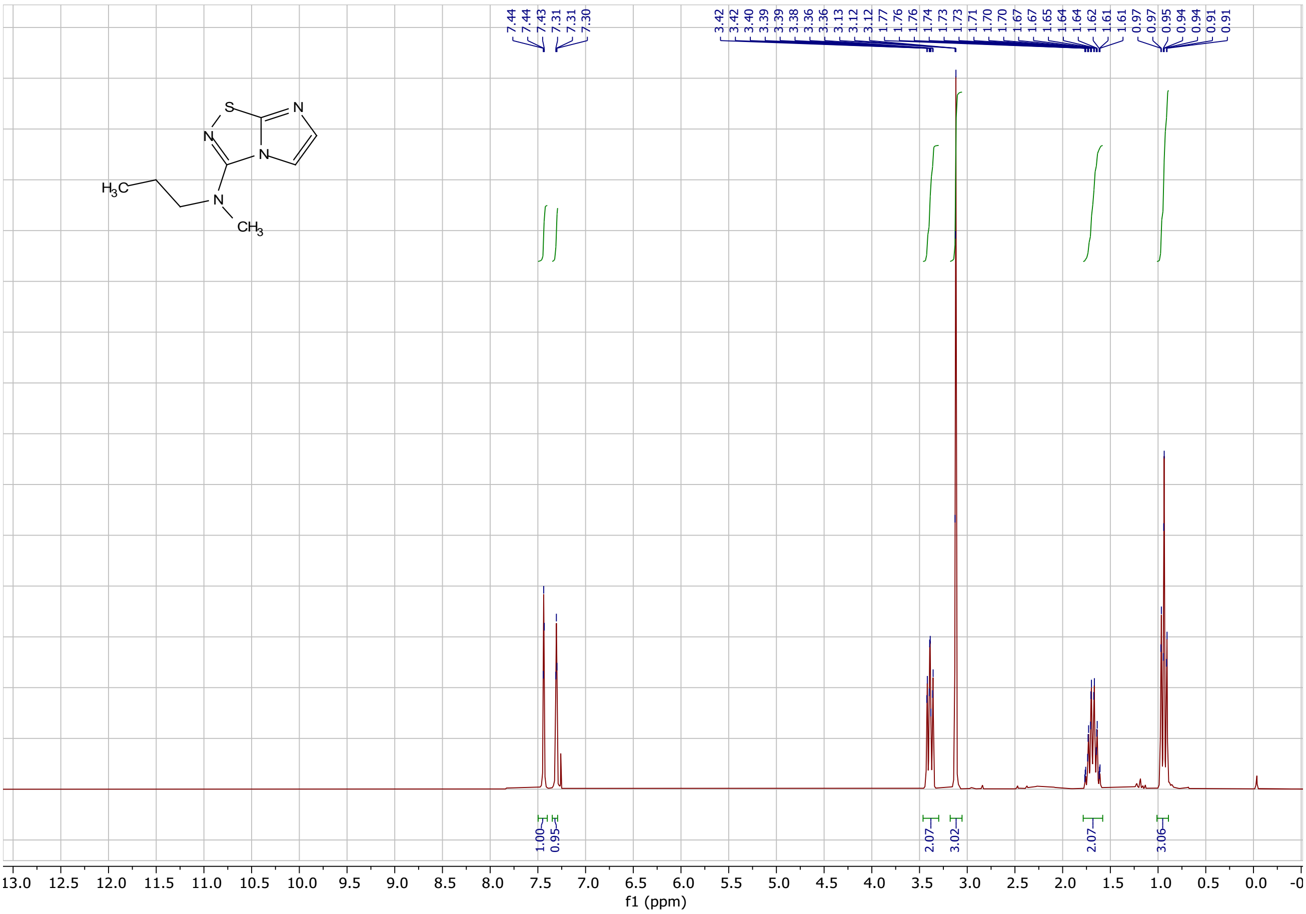

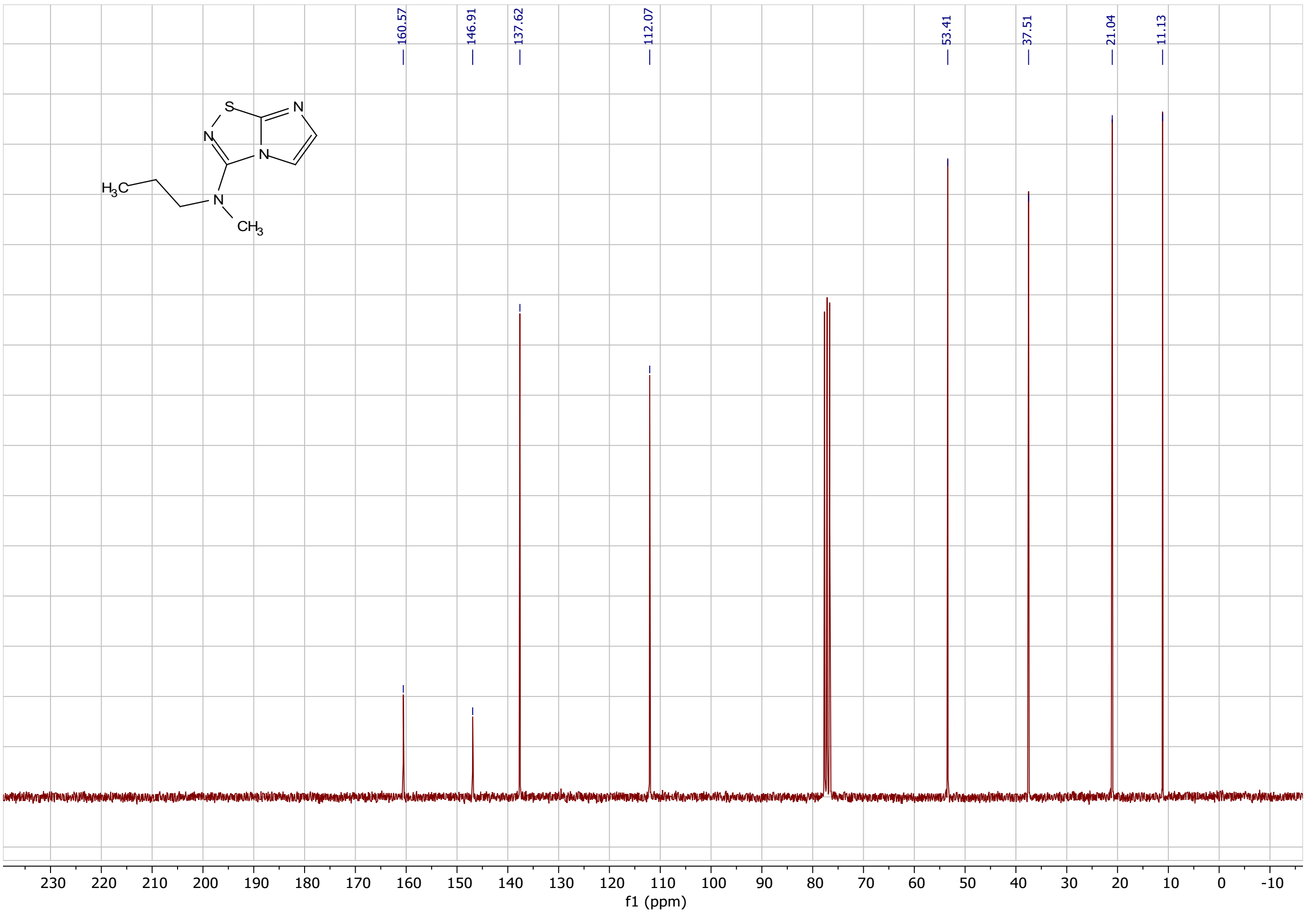

N-cyclopropylimidazo[1,2-*d*][1,2,4]thiadiazol-3-amine (7)

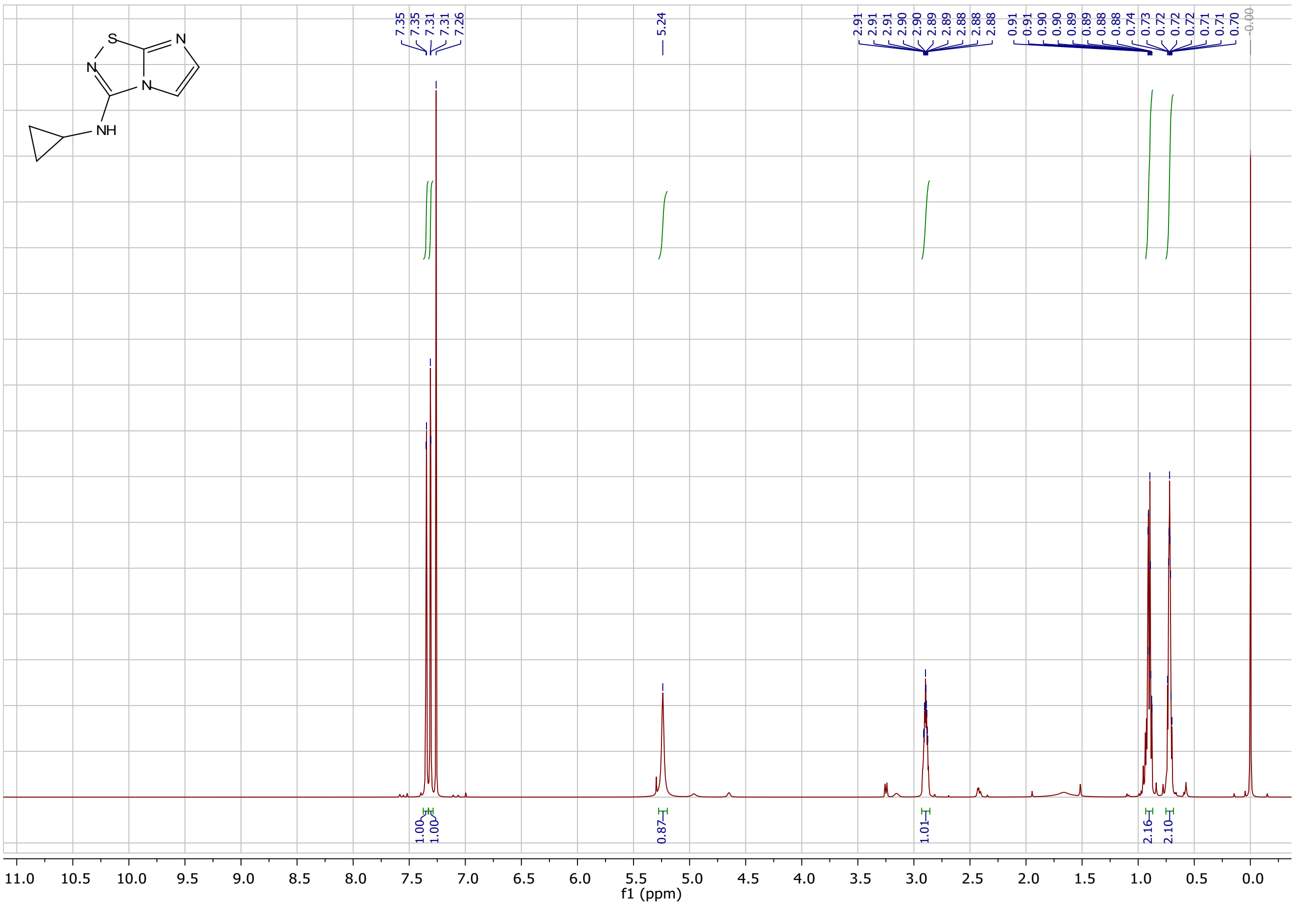

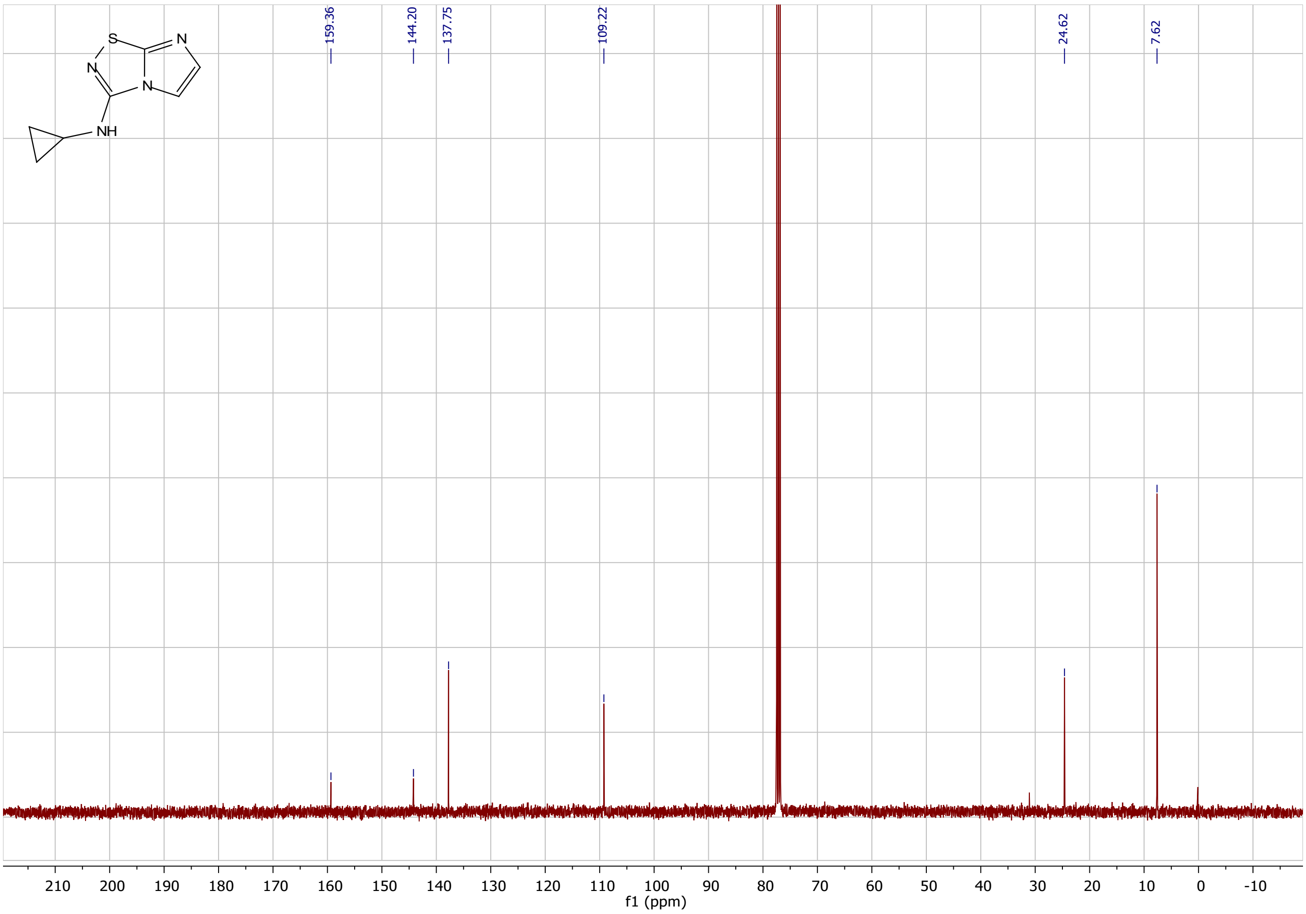

N-cyclohexylimidazo[1,2-*d*][1,2,4]thiadiazol-3-amine (8)

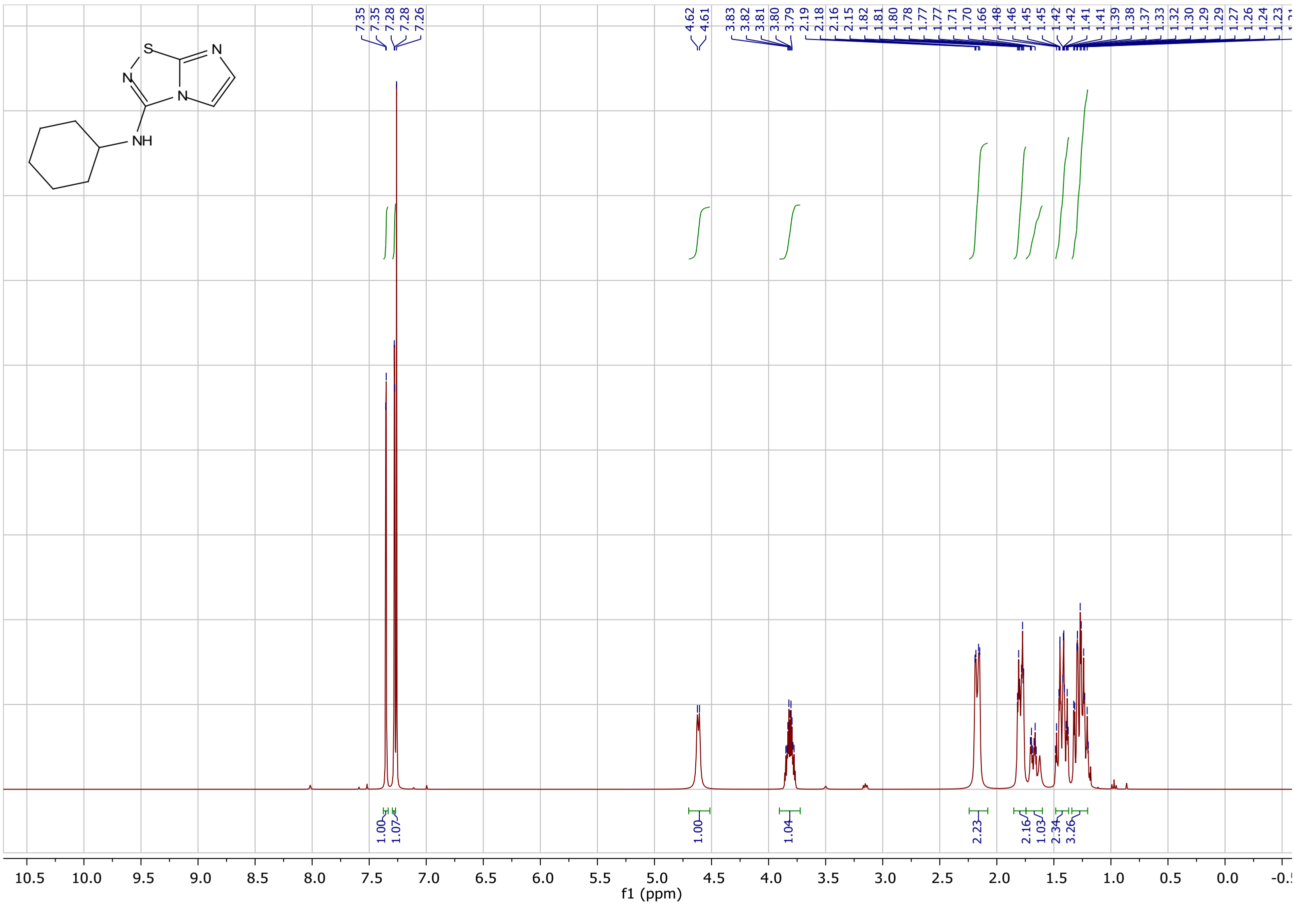

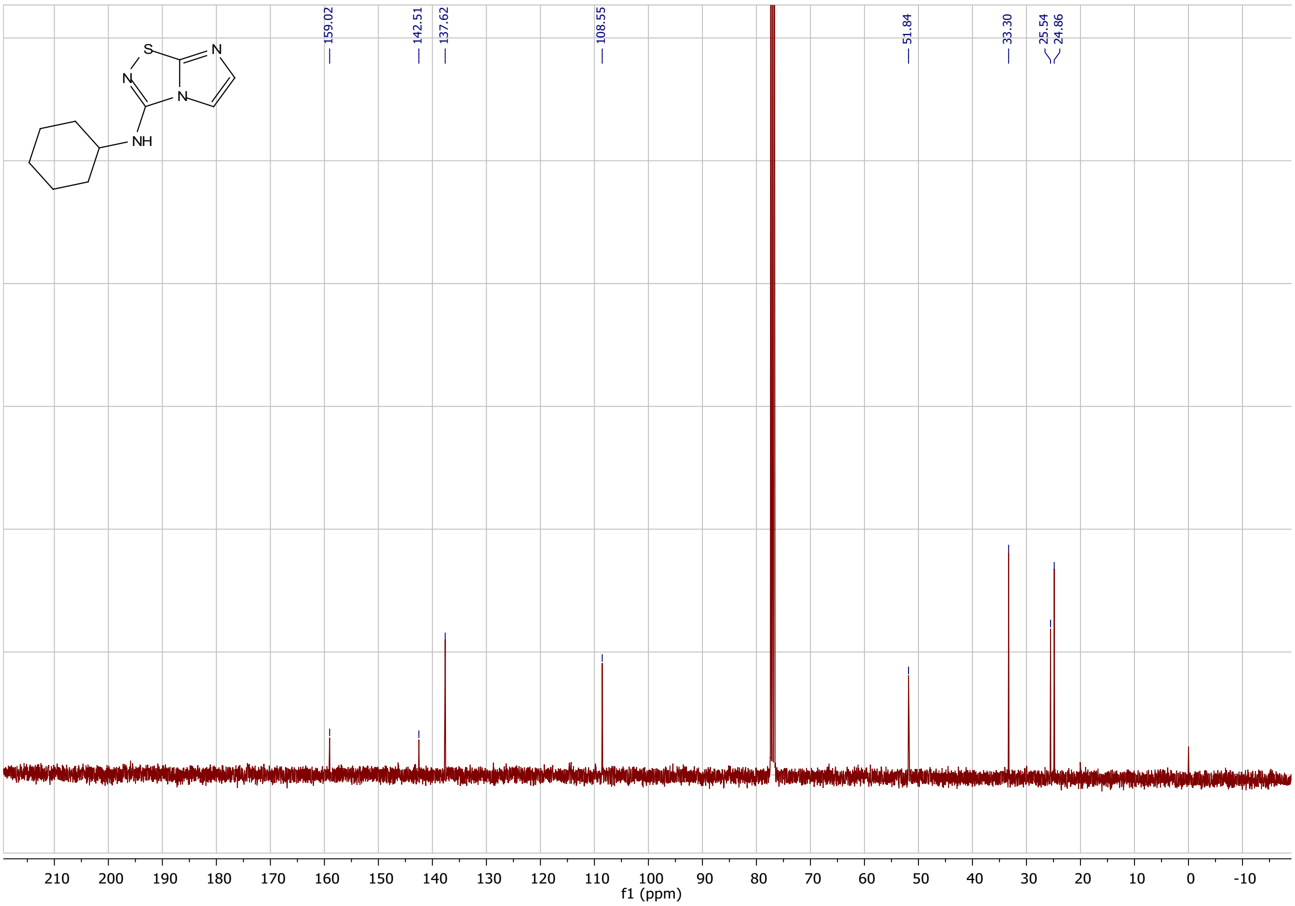

3-(piperidin-1-yl)imidazo[1,2-*d*][1,2,4]thiadiazole (9)

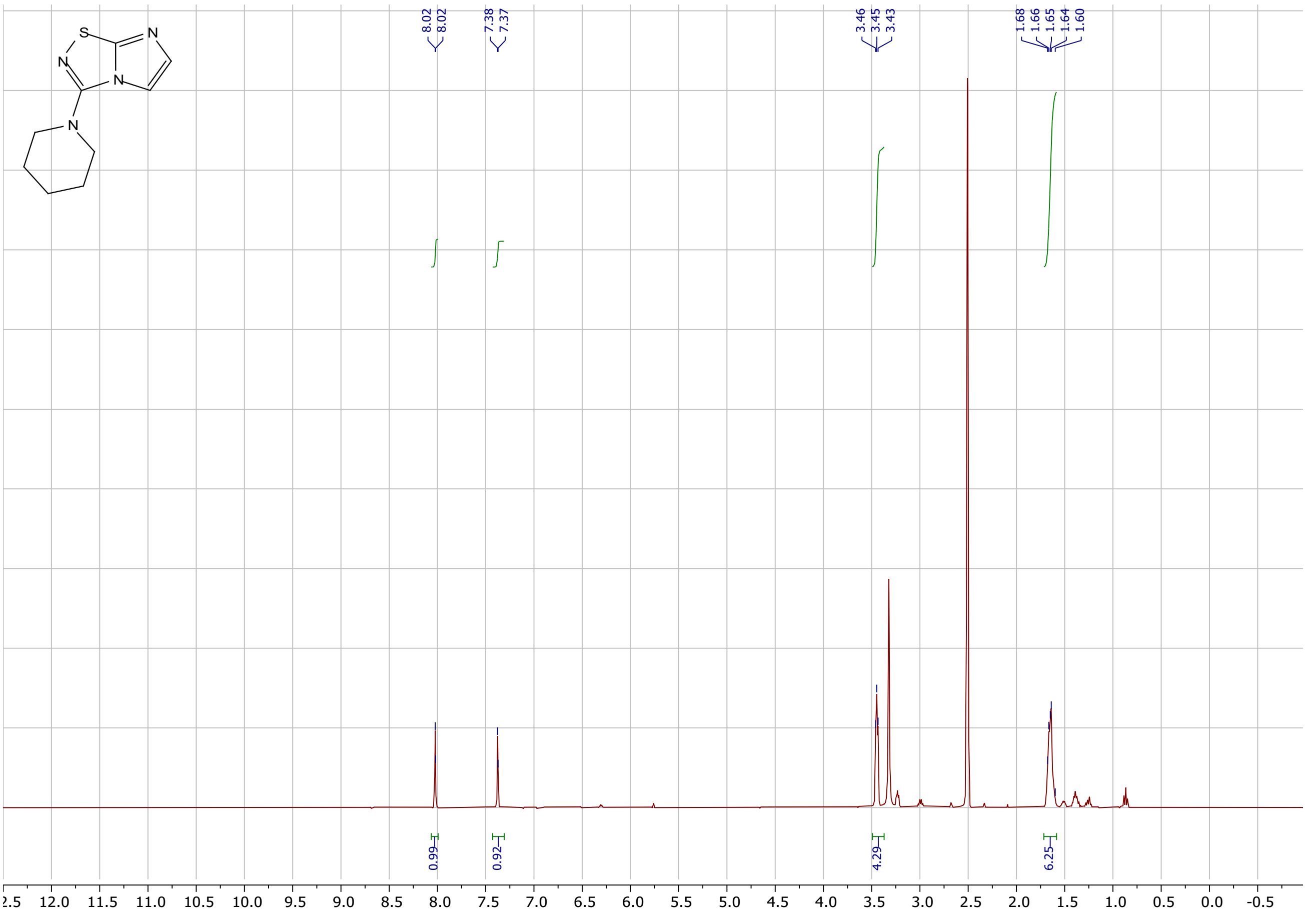

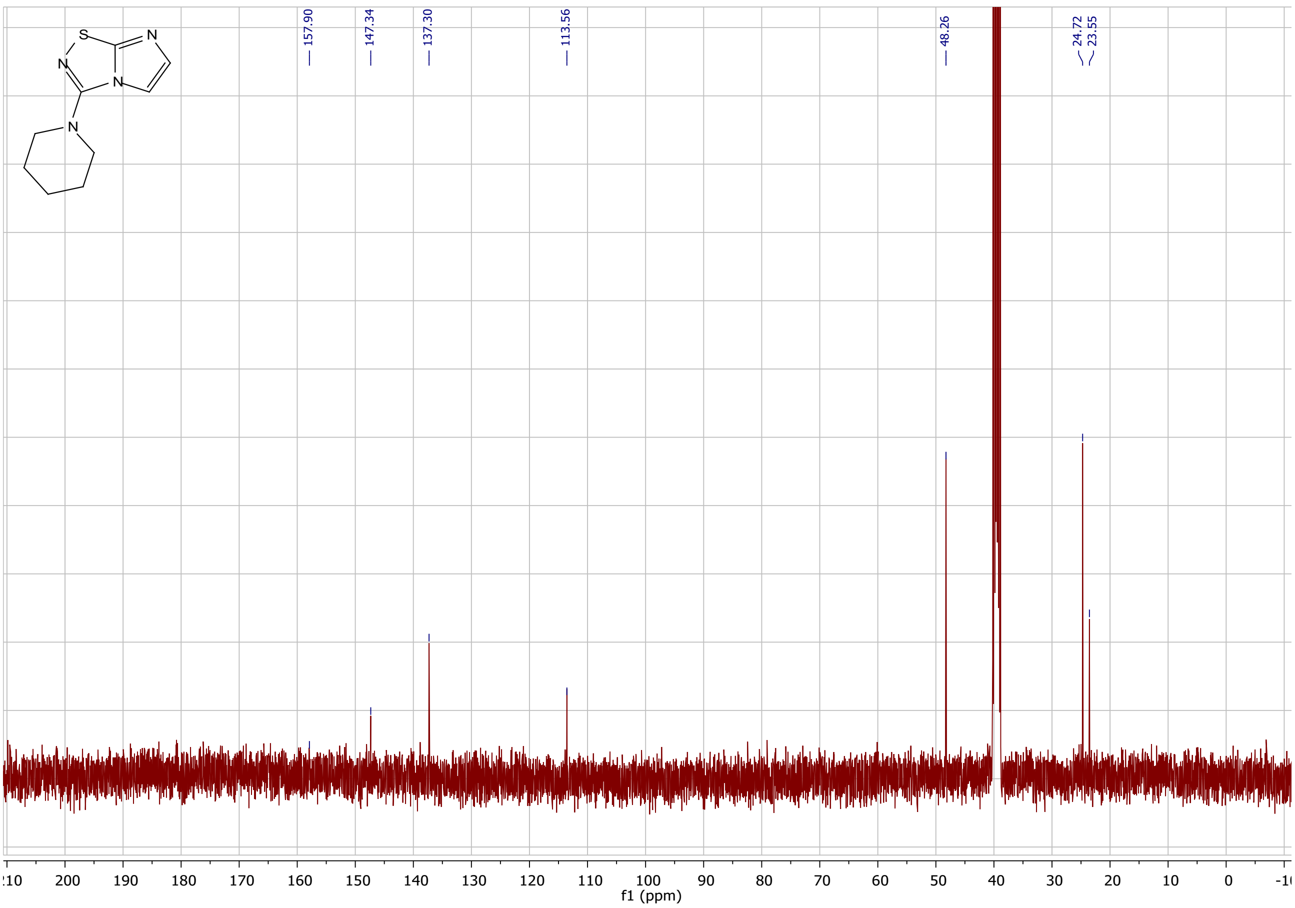

3-(4-methylpiperazin-1-yl)imidazo[1,2-*d*][1,2,4]thiadiazole (10)

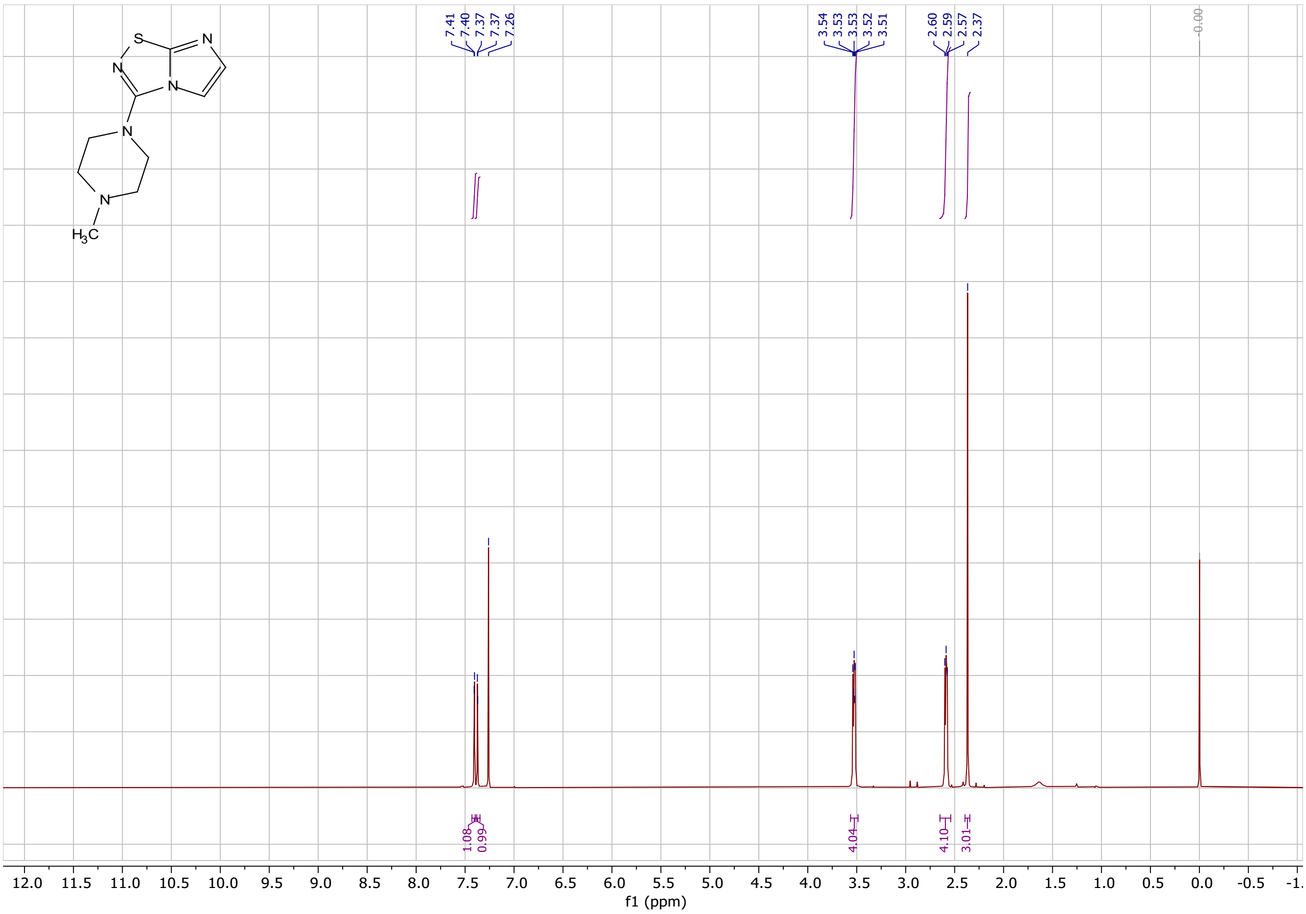

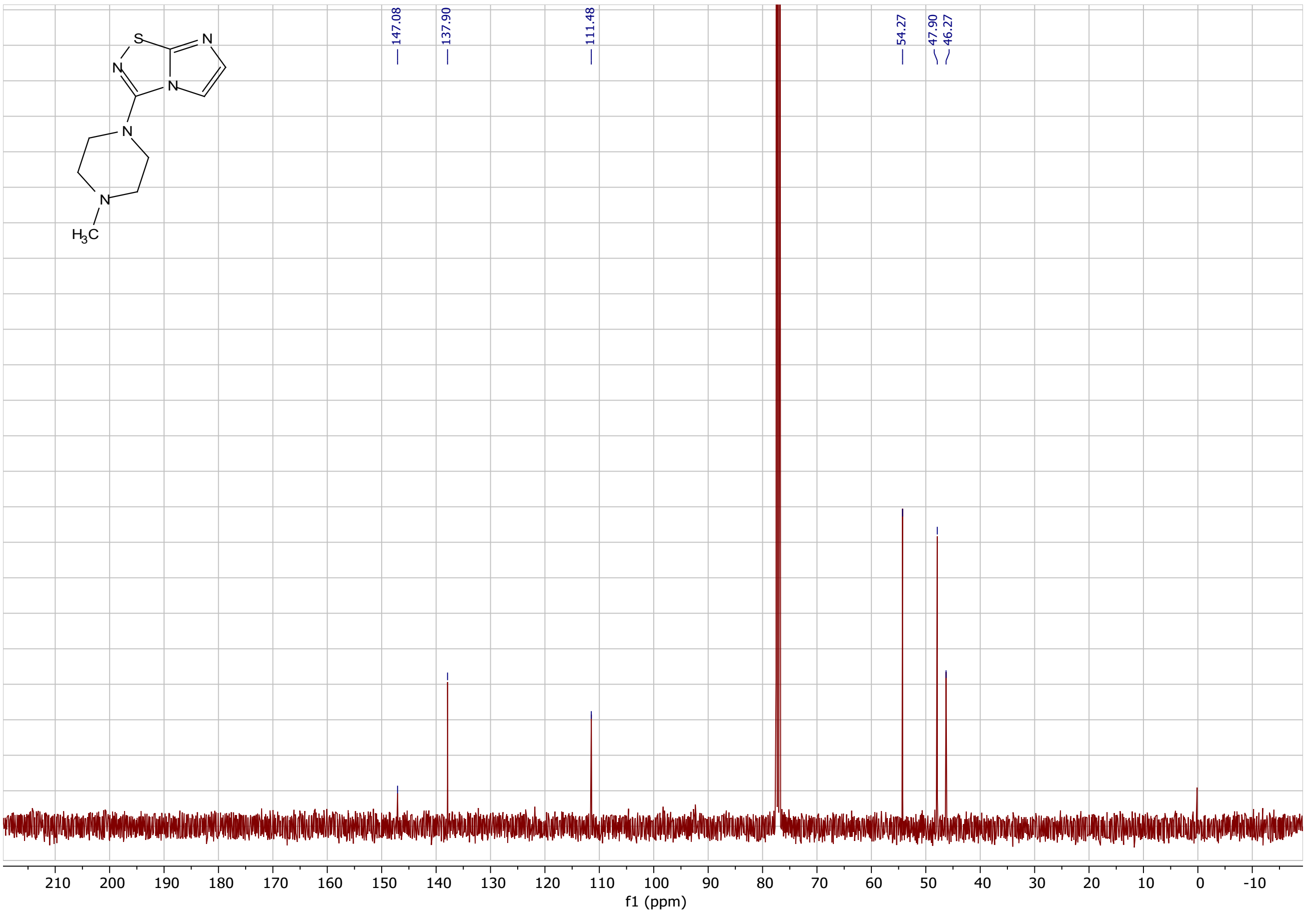

3-(Morpholin-4-yl)imidazo[1,2-*d*][1,2,4]thiadiazole (11)

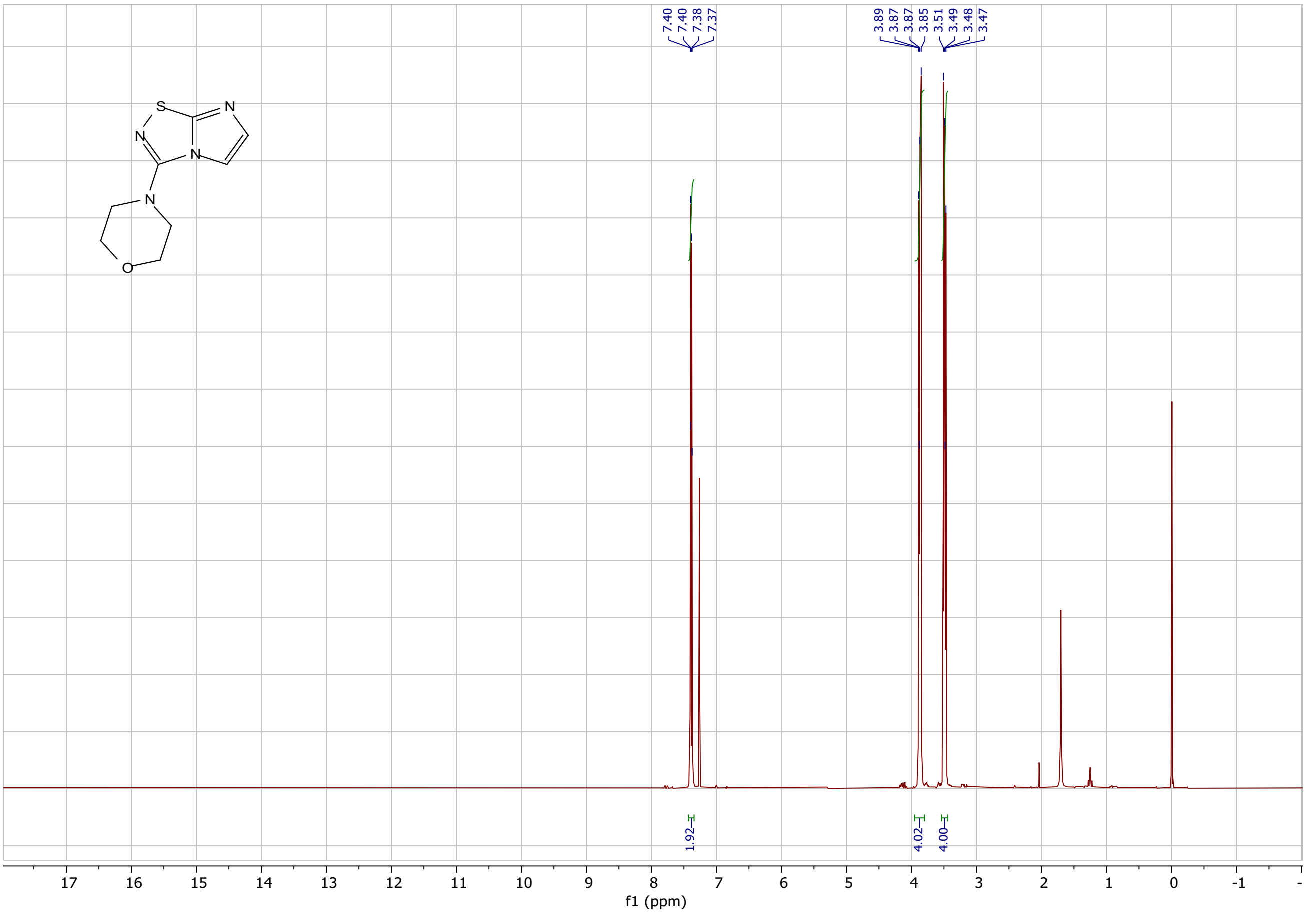

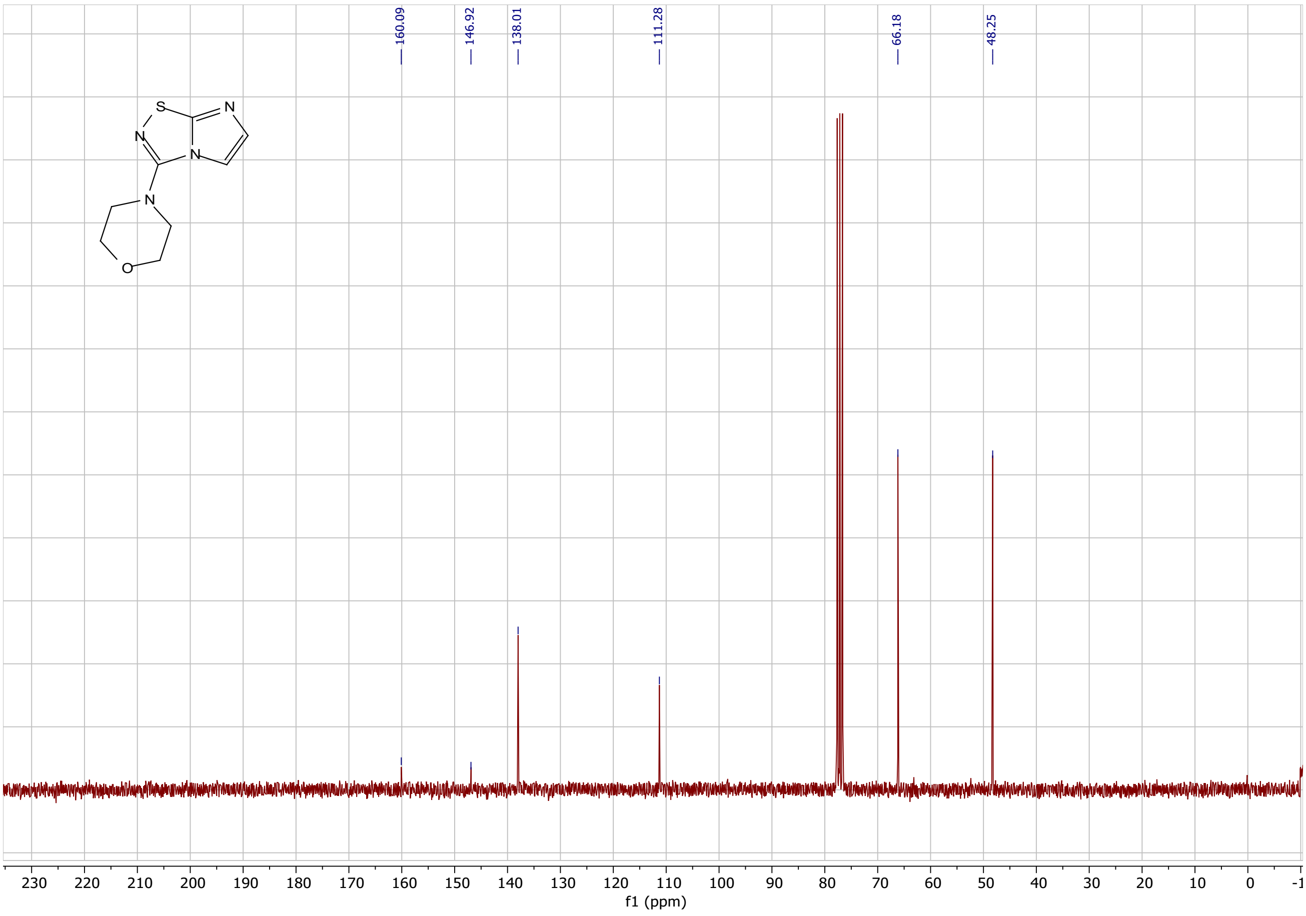

***N*-benzylimidazo[1,2-*d*][1,2,4]thiadiazol-3-amine (12)**

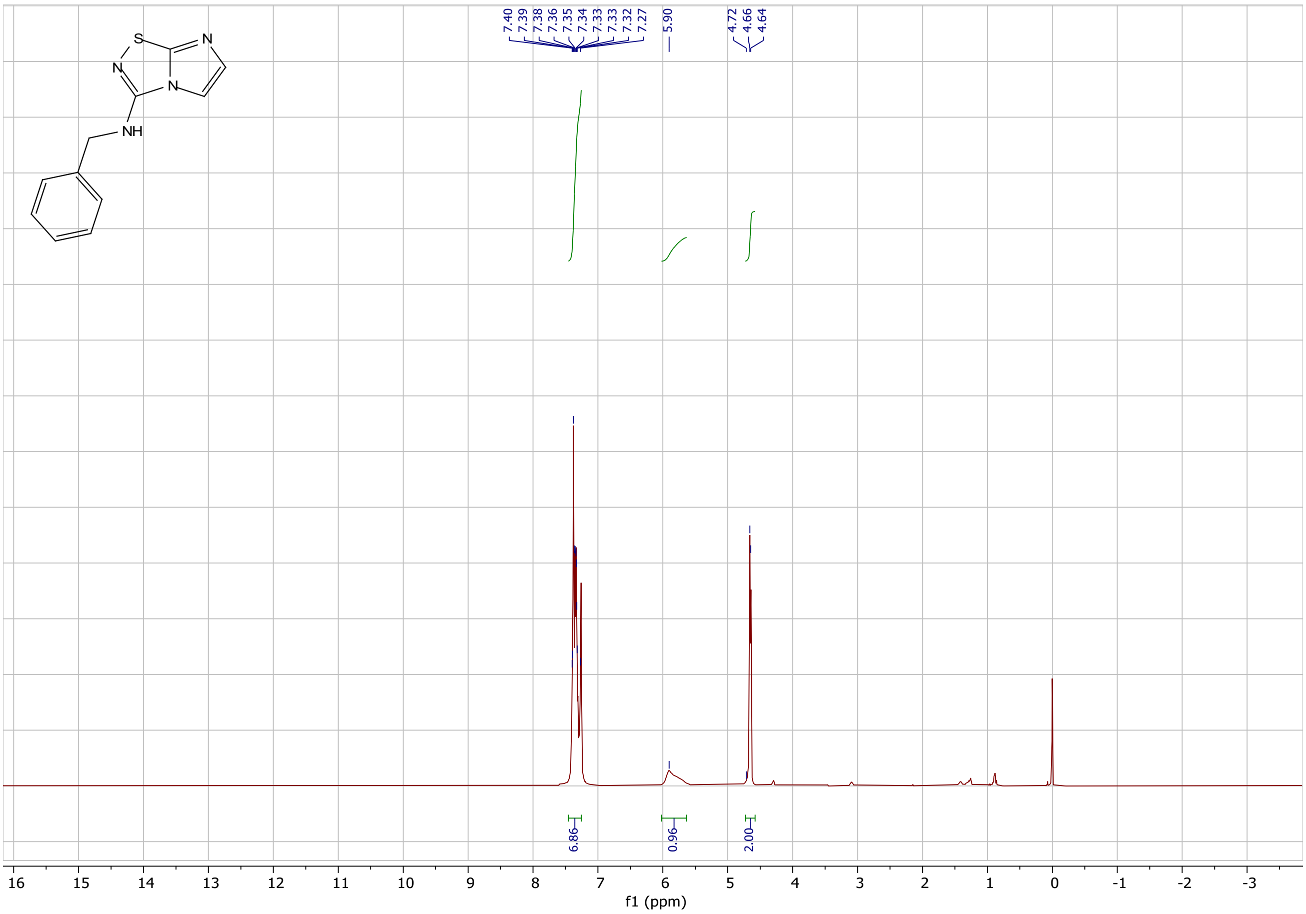

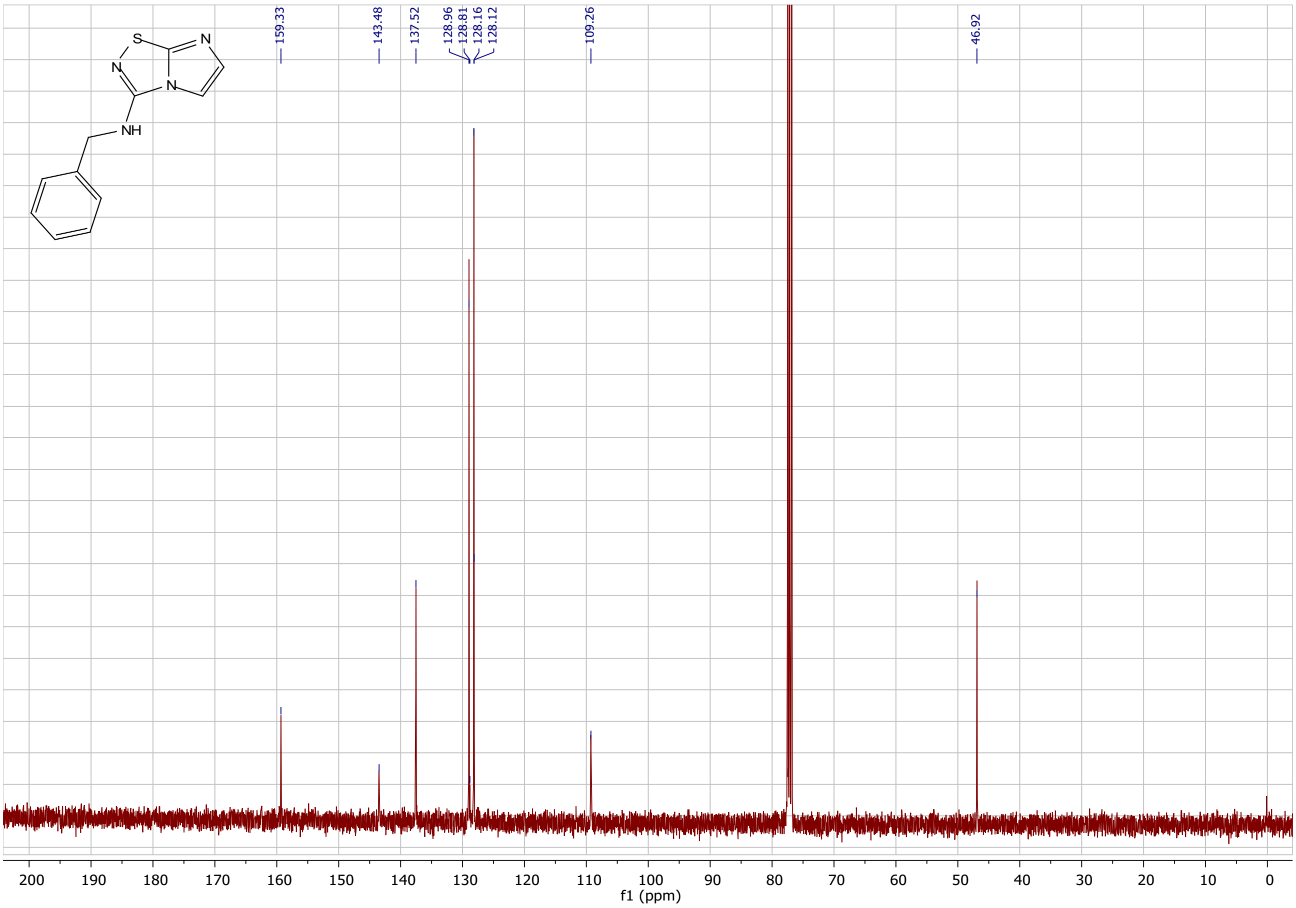

3-Methoxyimidazo[1,2-*d*][1,2,4]thiadiazole (14)

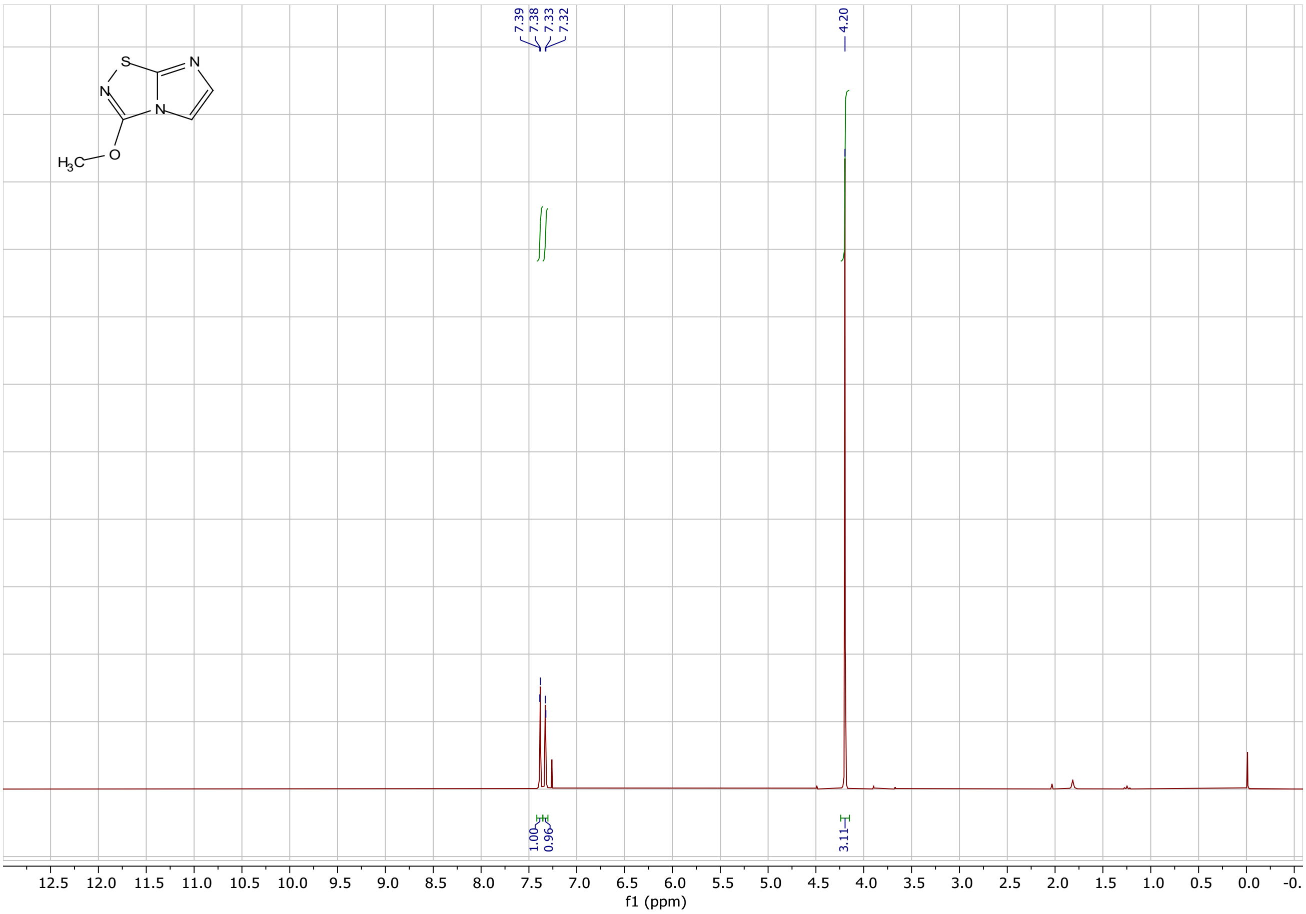

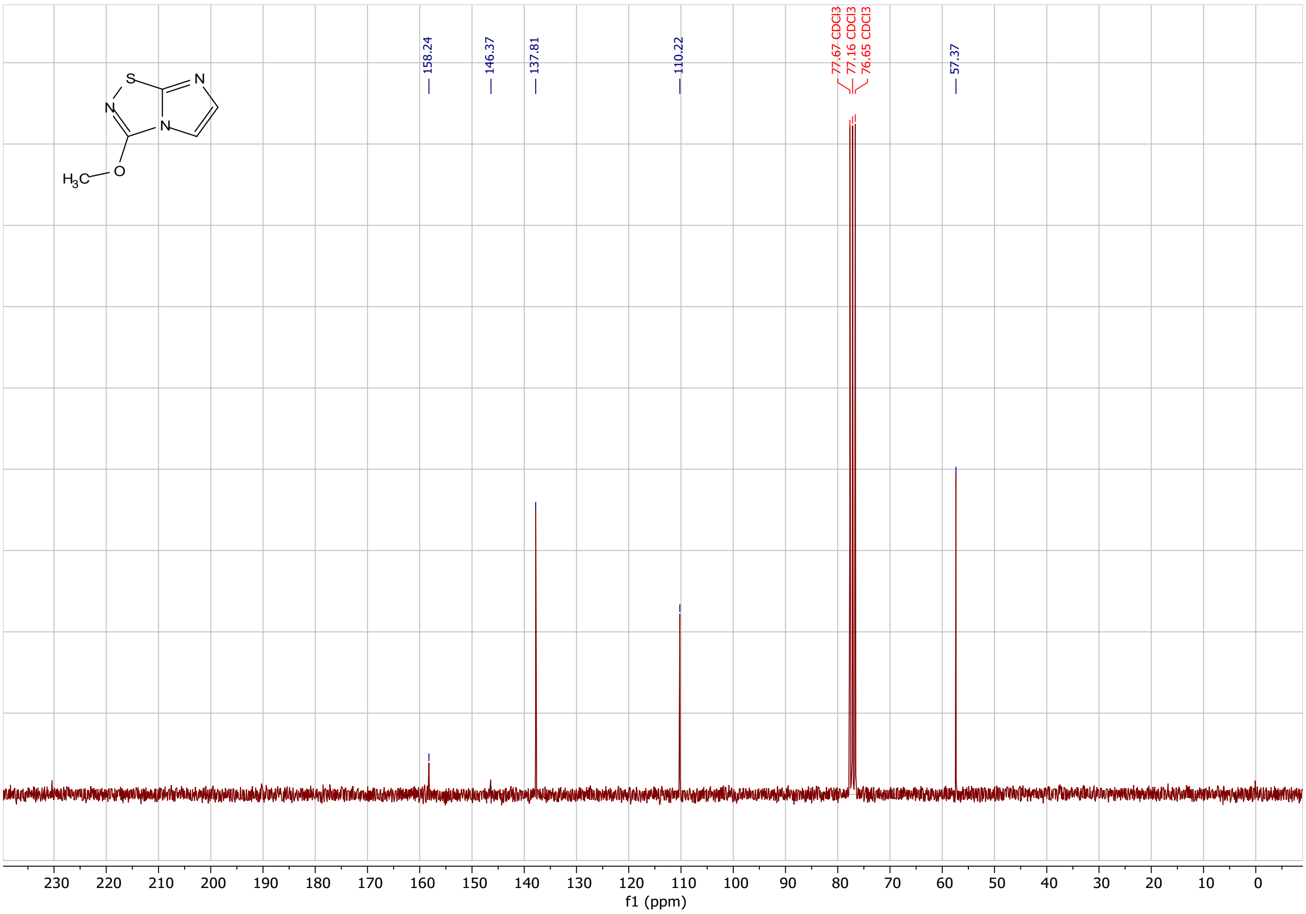

3-Ethoxyimidazo[1,2-*d*][1,2,4]thiadiazole (15)

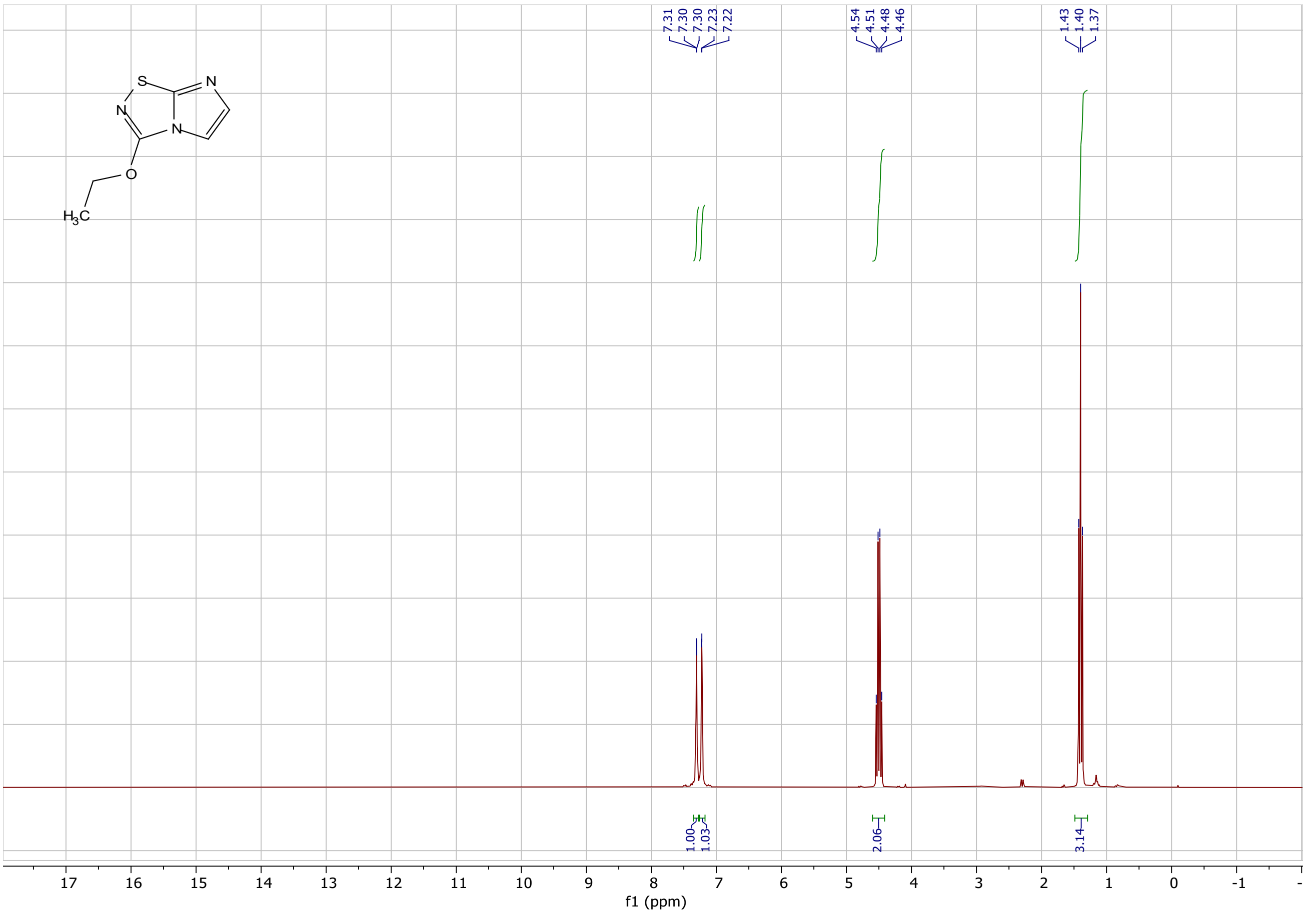

7.31
7.30
7.30
7.23
7.22
4.54
4.51
4.48
4.46
1.43
1.40
1.37
1.00
1.03
2.06
3.14
f1 (ppm)

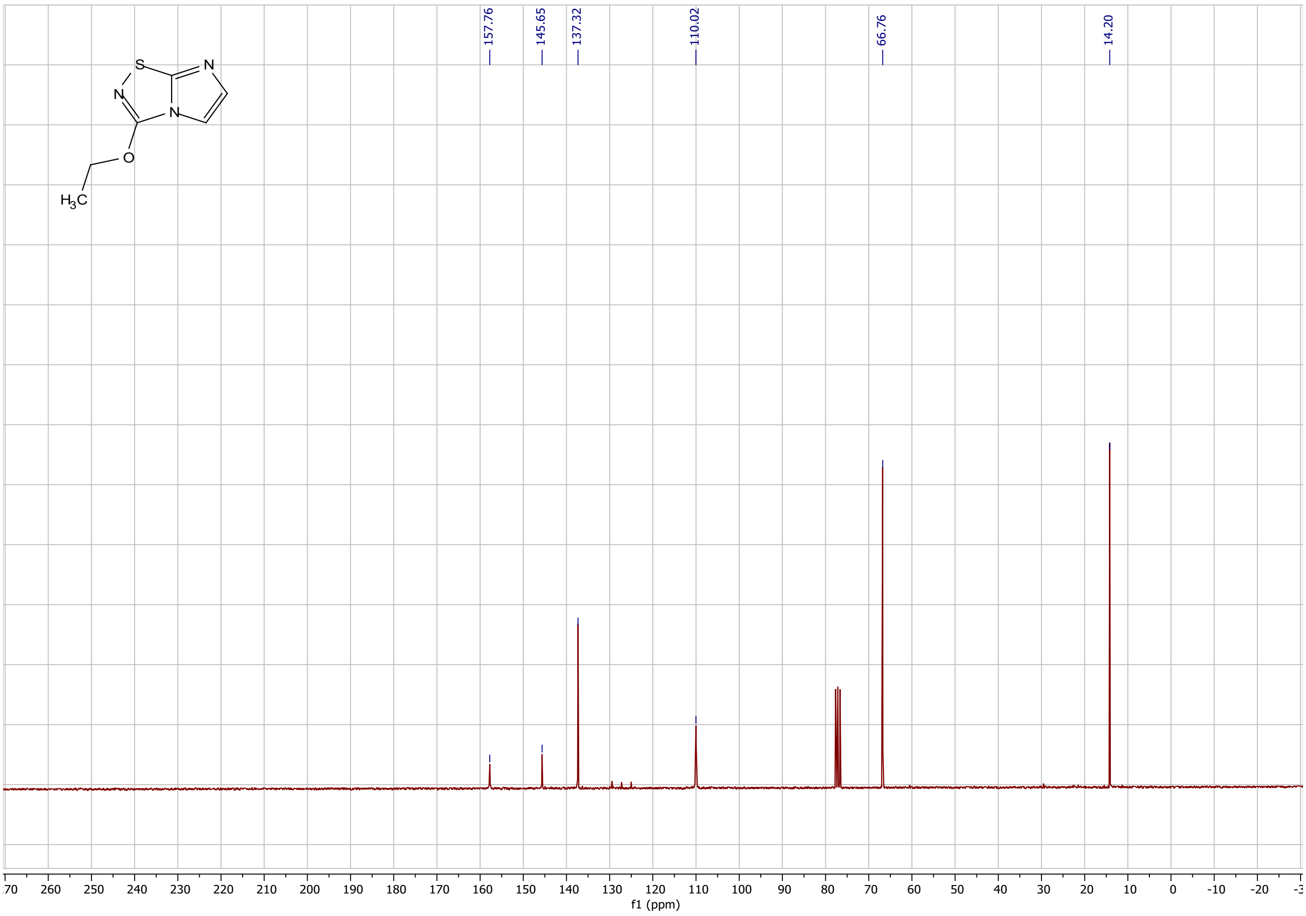

157.76
145.65
137.32
110.02
66.76
14.20
f1 (ppm)

5-iodo-*N*-propylimidazo[1,2-*d*][1,2,4]thiadiazol-3-amine (16)

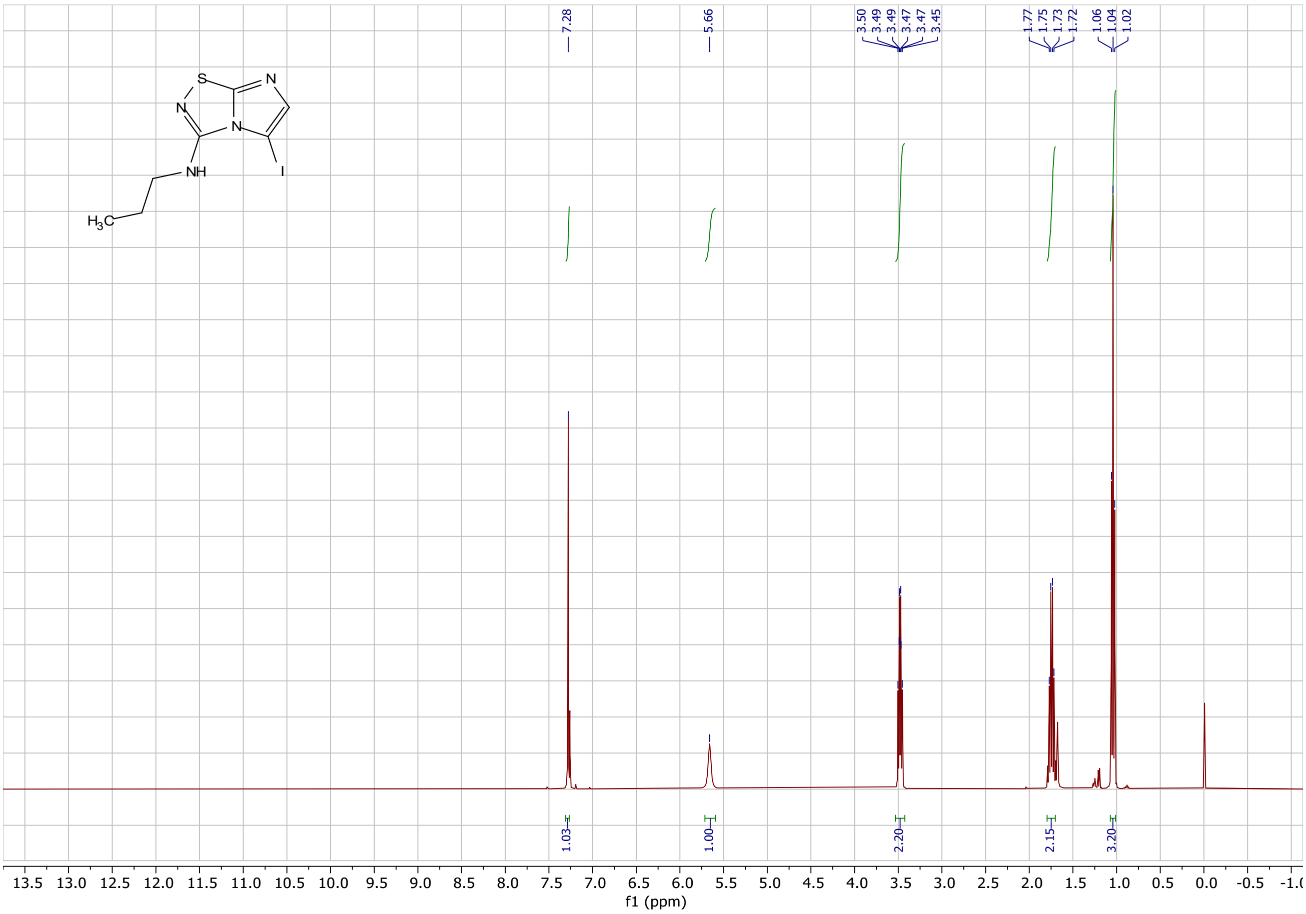

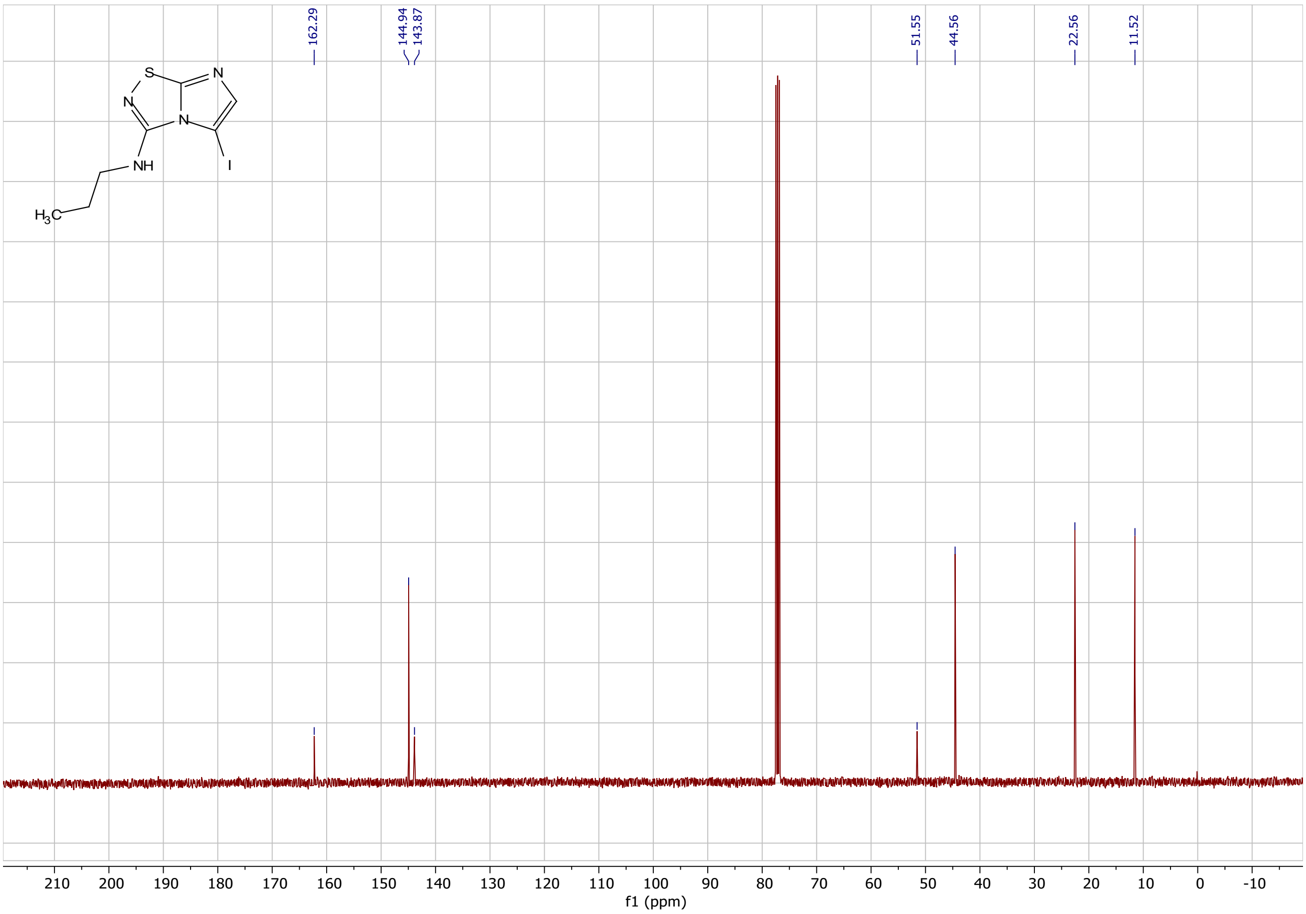

5-iodo-*N*-methyl-*N*-propylimidazo[1,2-*d*][1,2,4]thiadiazol-3-amine (17)

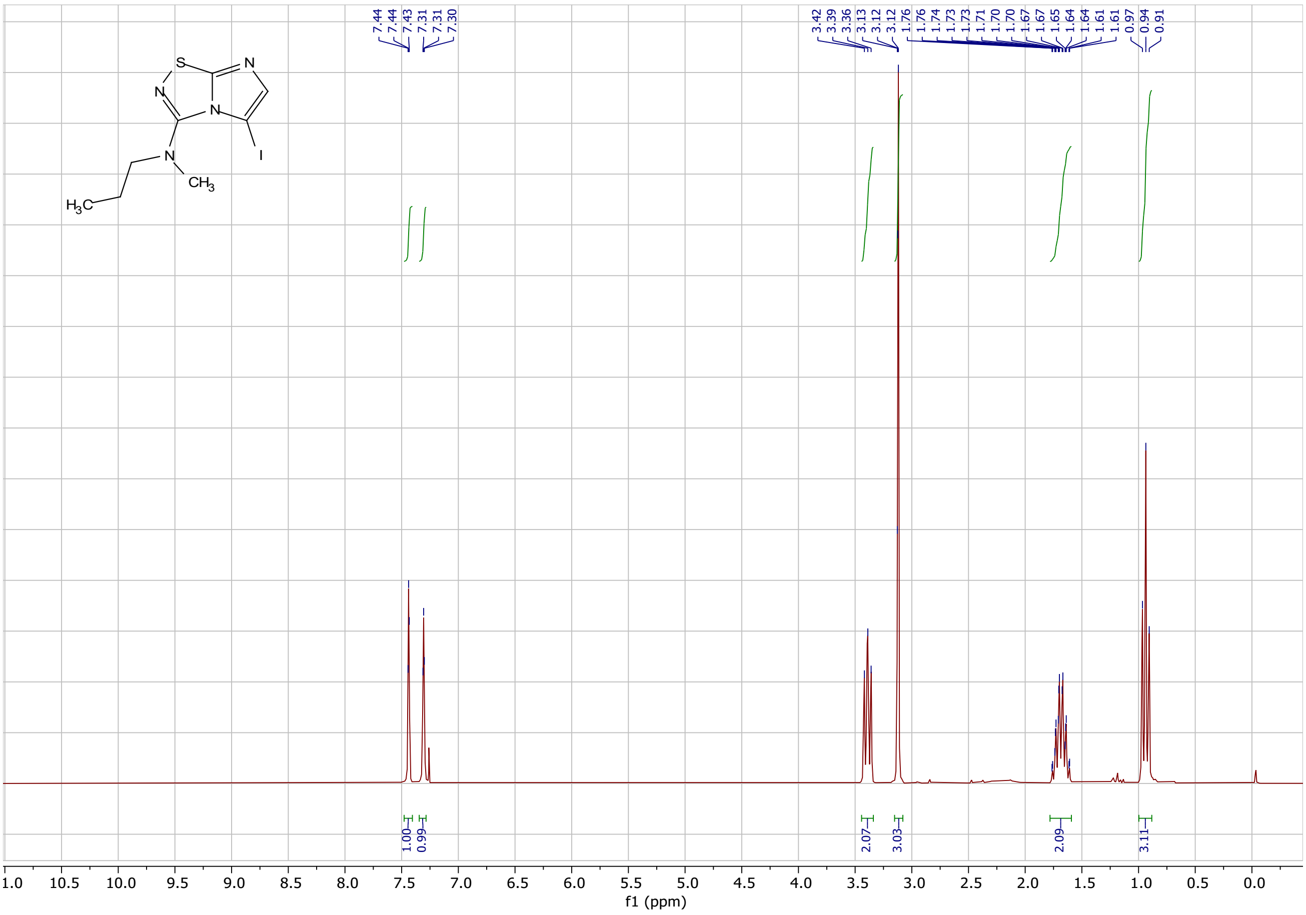

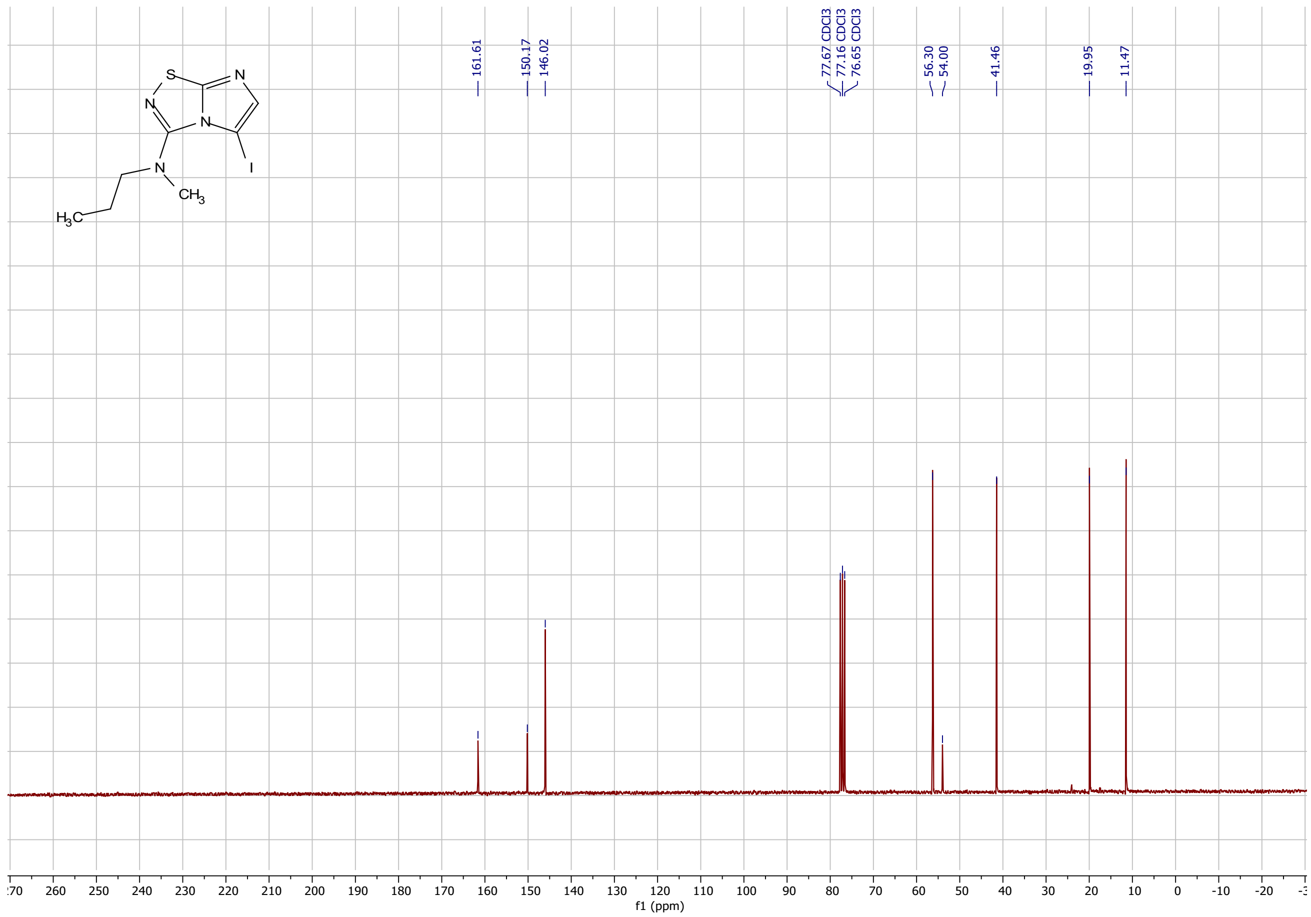

N-cyclopropyl-5-iodoimidazo[1,2-d][1,2,4]thiadiazol-3-amine (18)

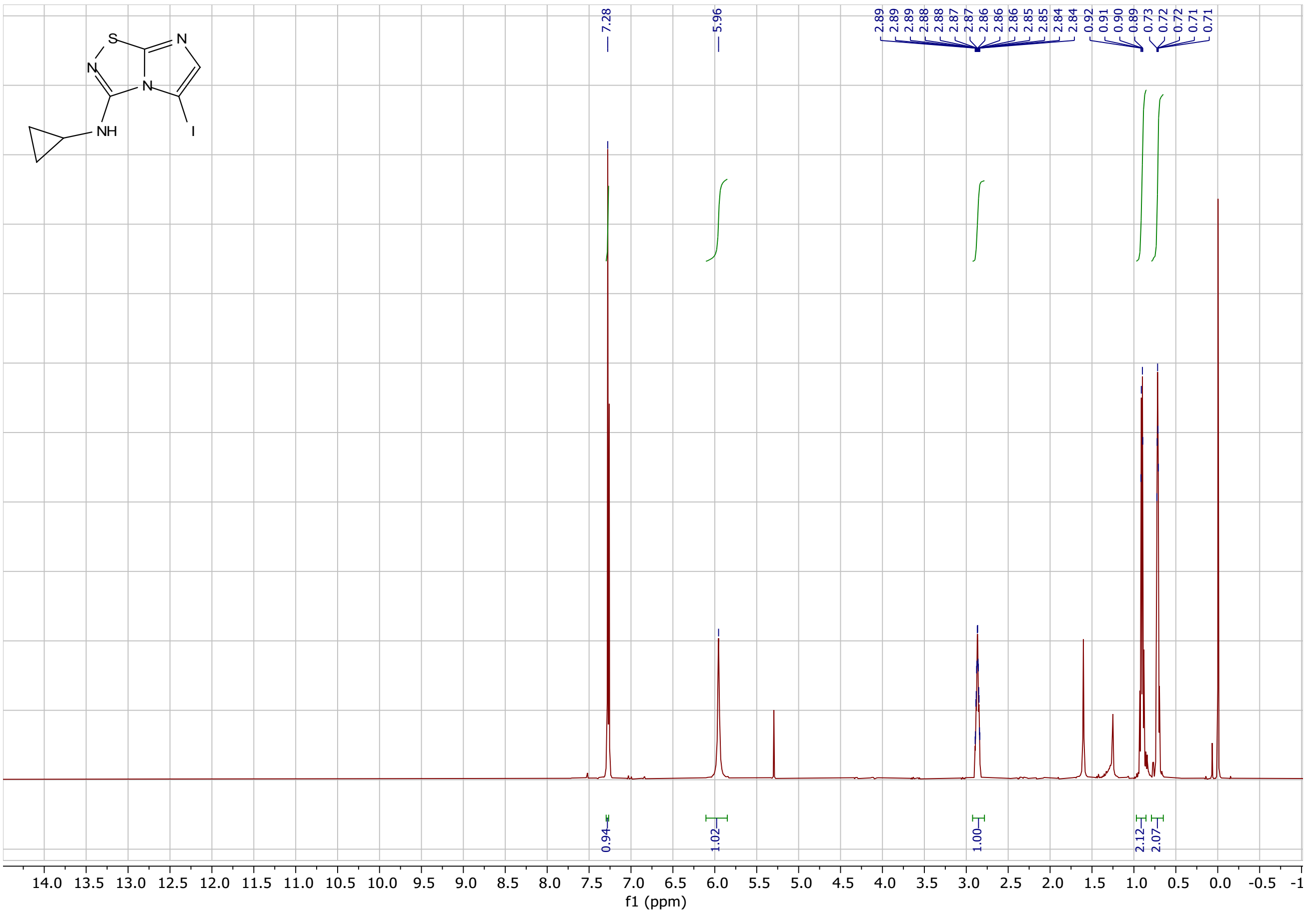

7.28
5.96
2.89
2.89
2.89
2.88
2.88
2.87
2.87
2.86
2.86
2.86
2.85
2.85
2.84
2.84
0.92
0.91
0.90
0.89
0.73
0.72
0.72
0.71
0.71
0.94
1.02
1.00
2.12
2.07
f1 (ppm)

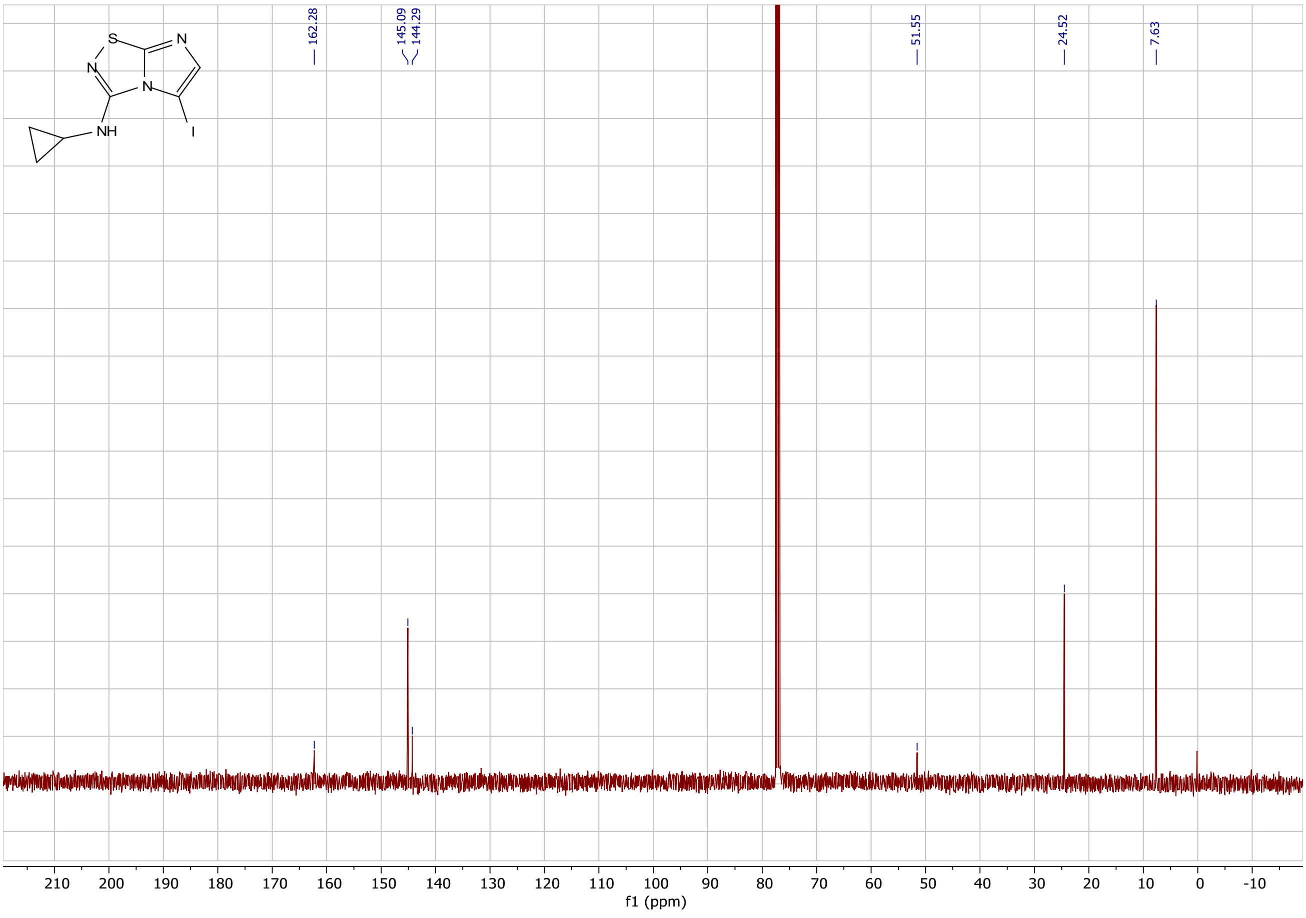

162.28
145.09
144.29
51.55
24.52
7.63
f1 (ppm)

N-cyclohexyl-5-iodoimidazo[1,2-*d*][1,2,4]thiadiazol-3-amine (19)

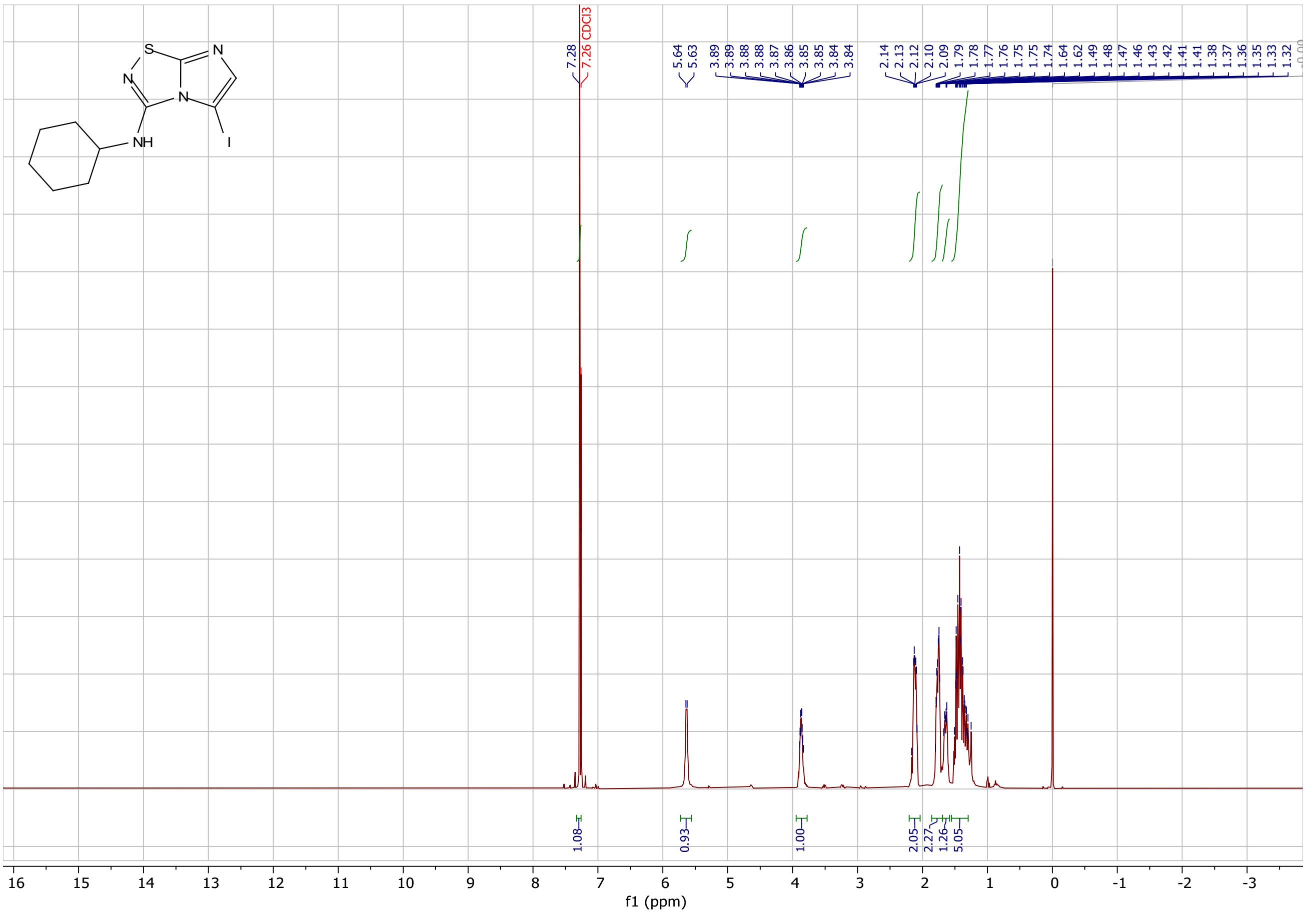

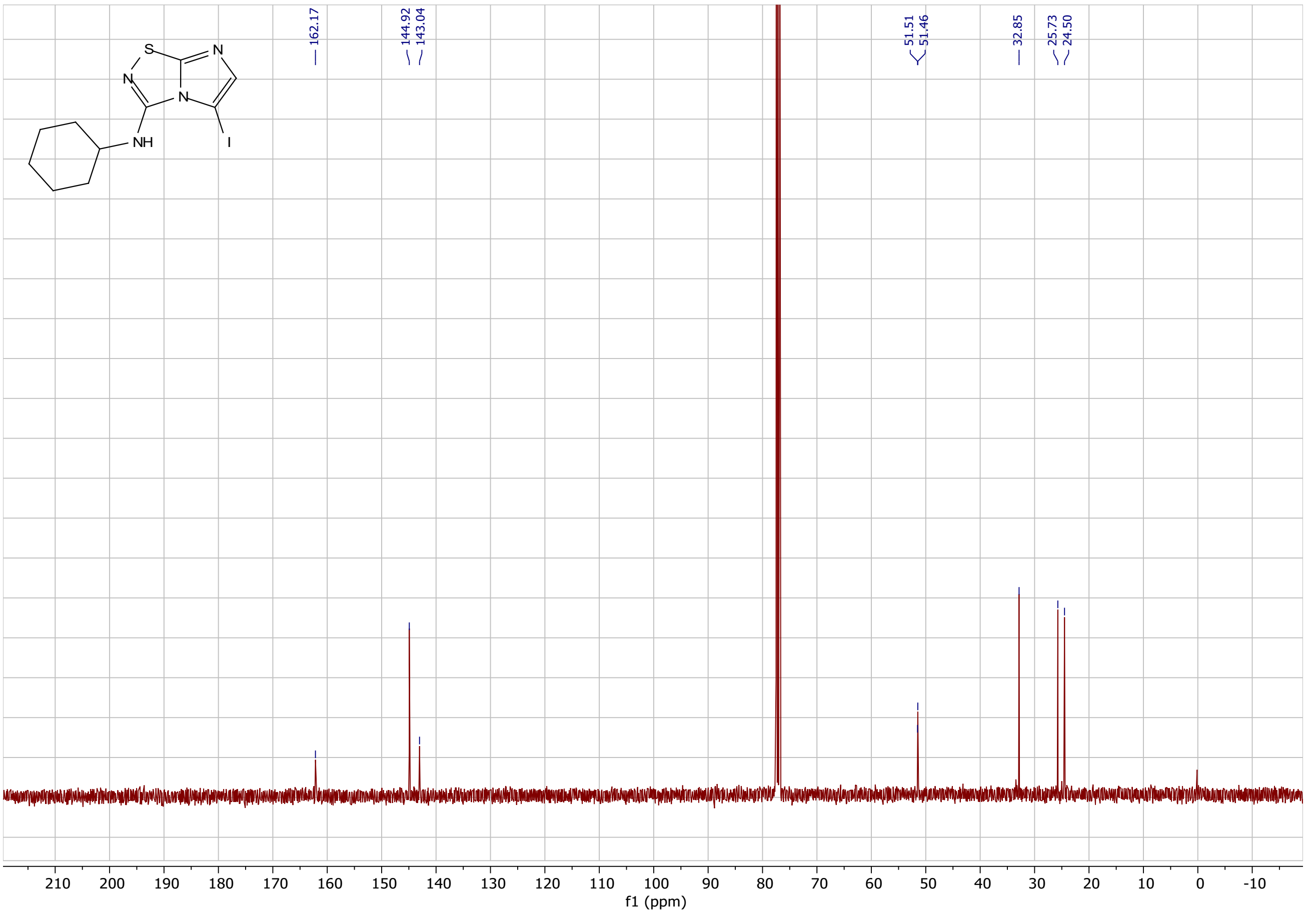

5-iodo-3-(piperidin-1-yl)imidazo[1,2-*d*][1,2,4]thiadiazole (20)

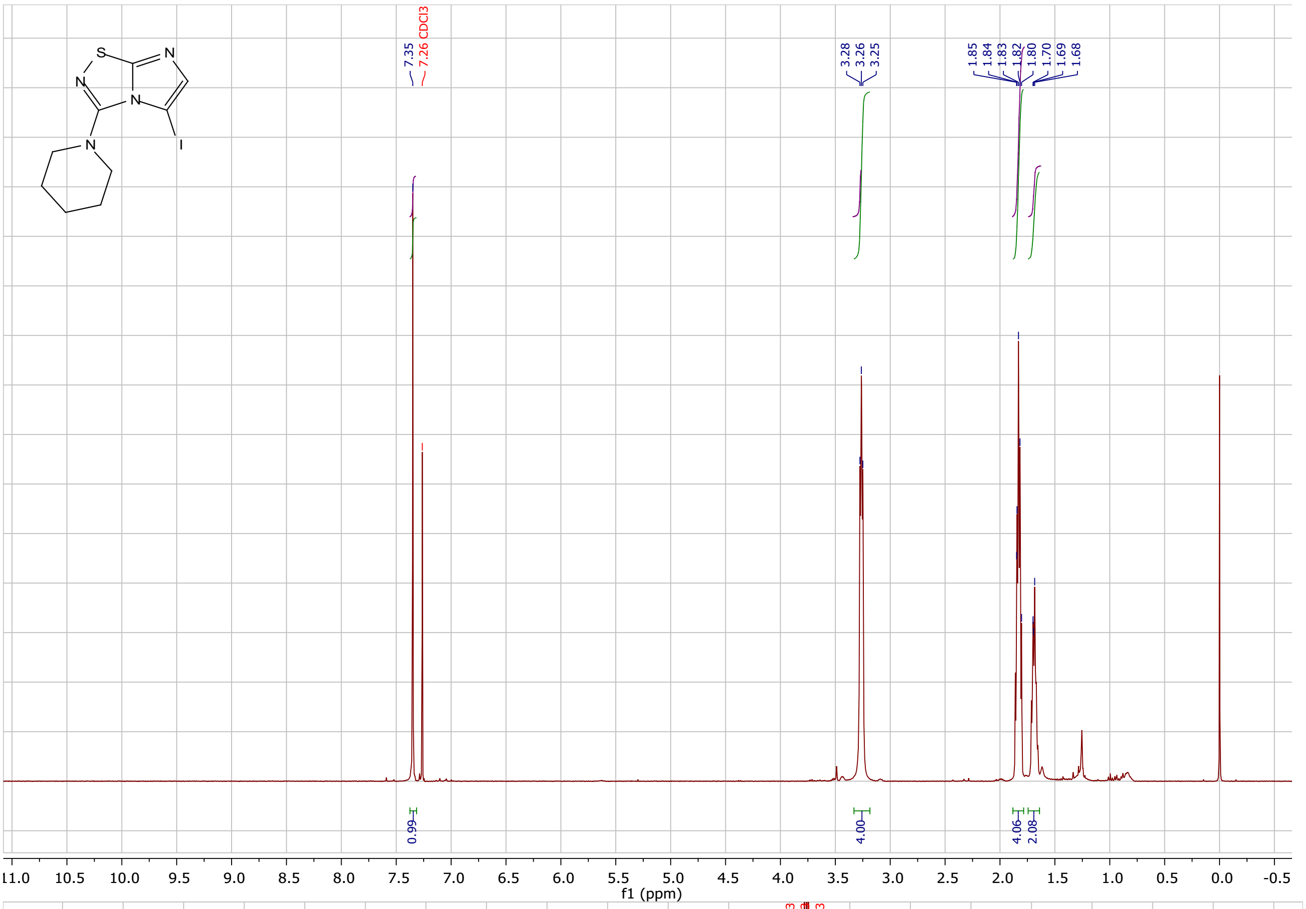

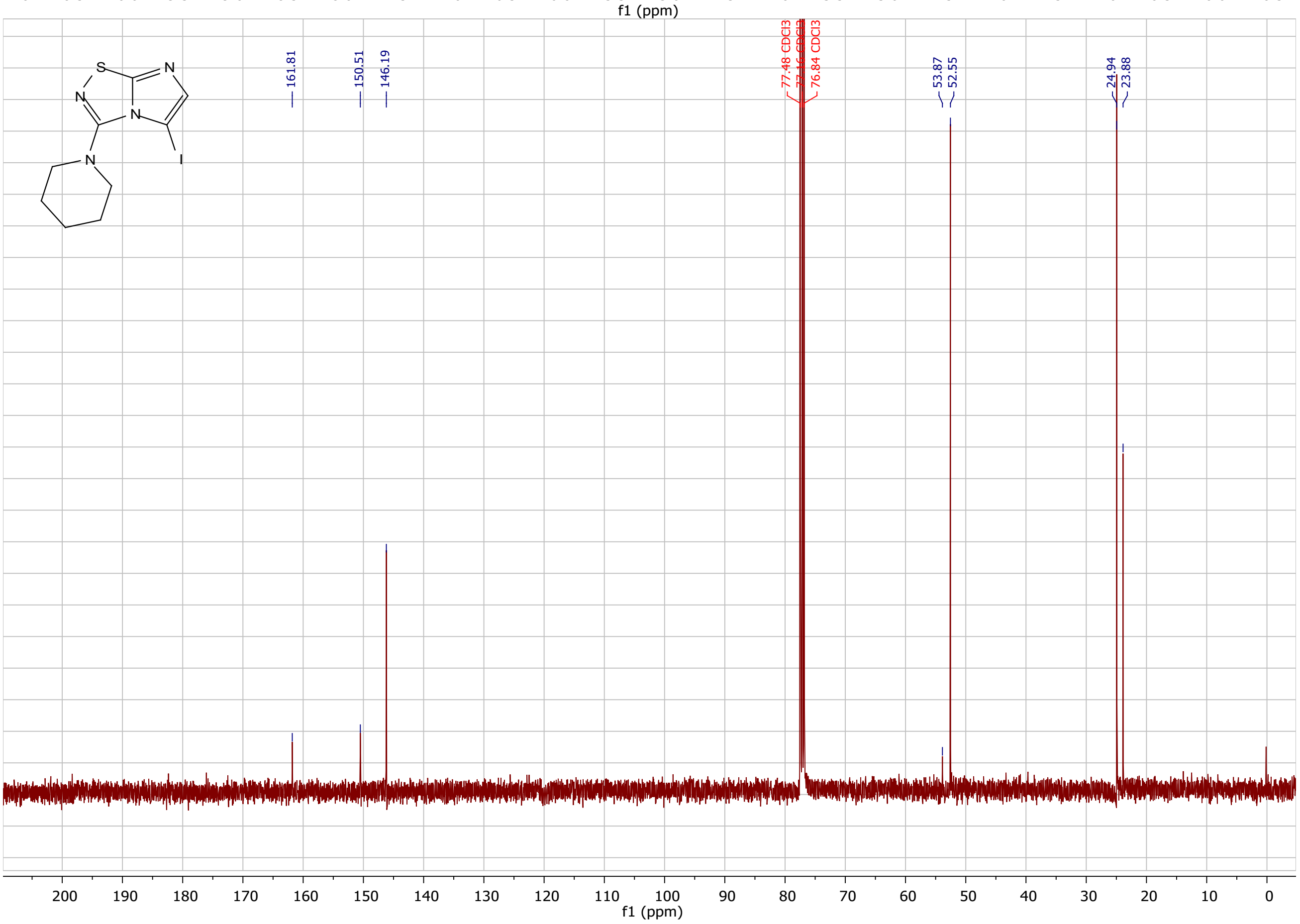

5-iodo-3-(4-methylpiperazin-1-yl)imidazo[1,2-*d*][1,2,4]thiadiazole (21)

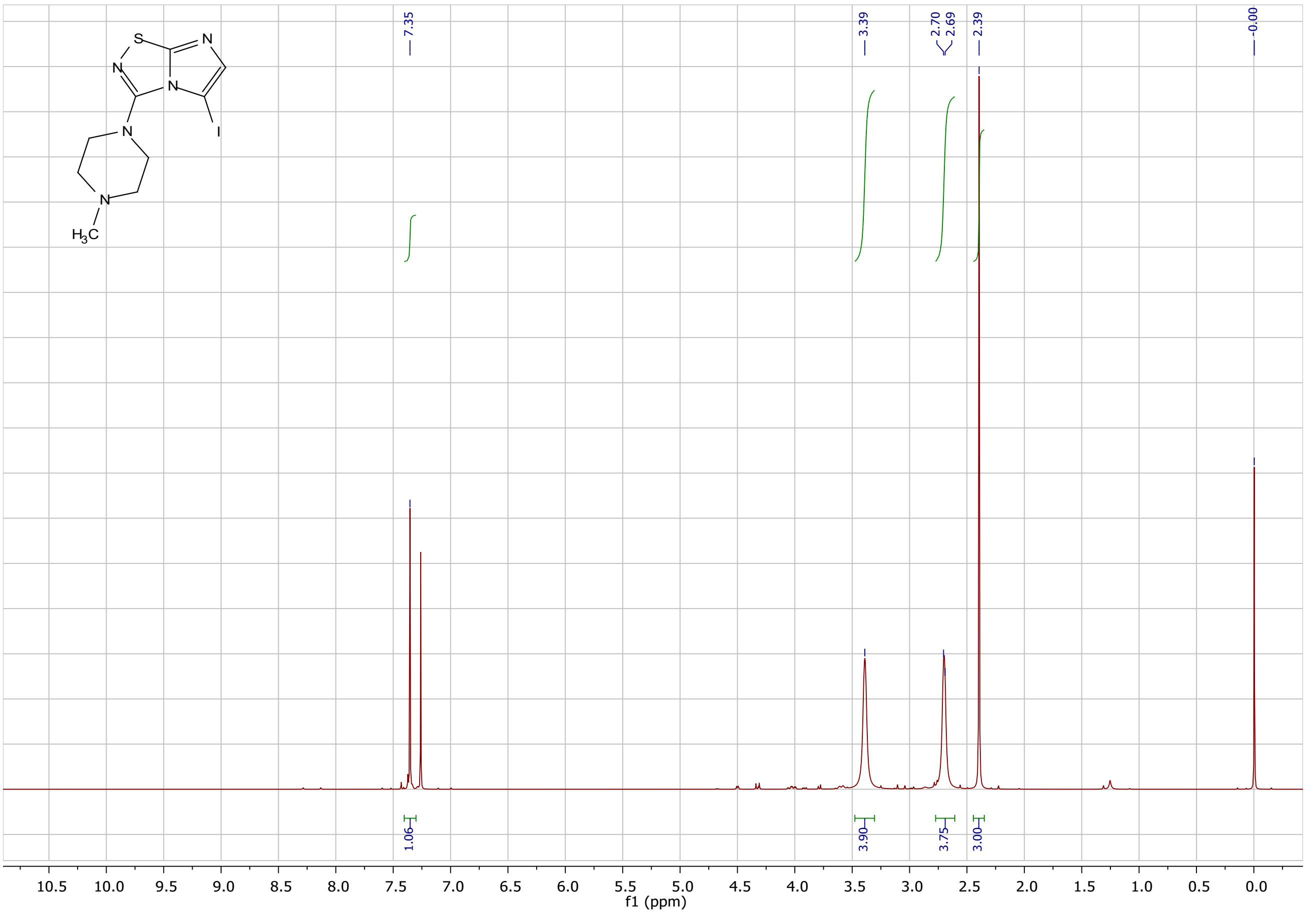

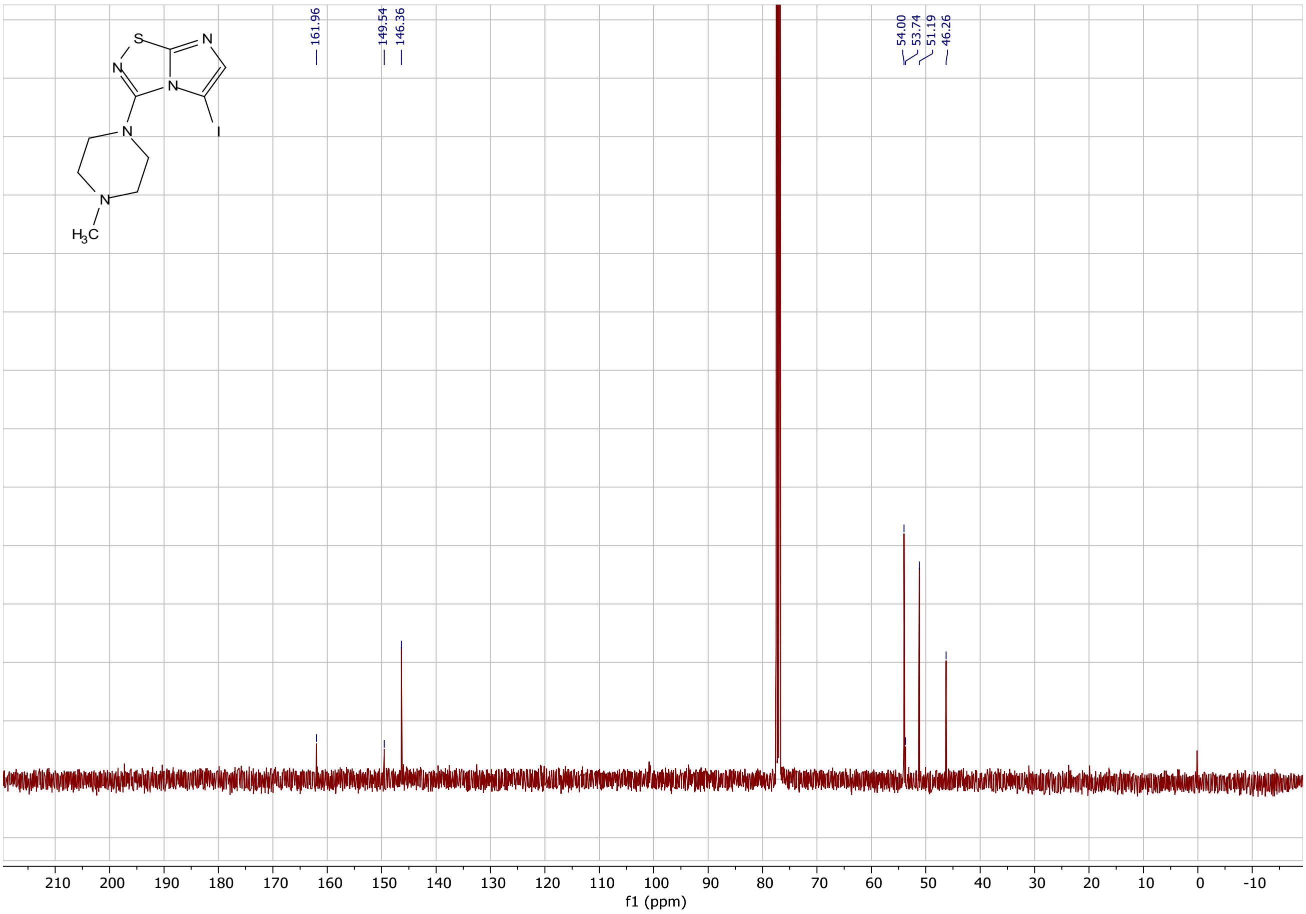

5-iodo-3-(morpholin-4-yl)imidazo[1,2-*d*][1,2,4]thiadiazole (22)

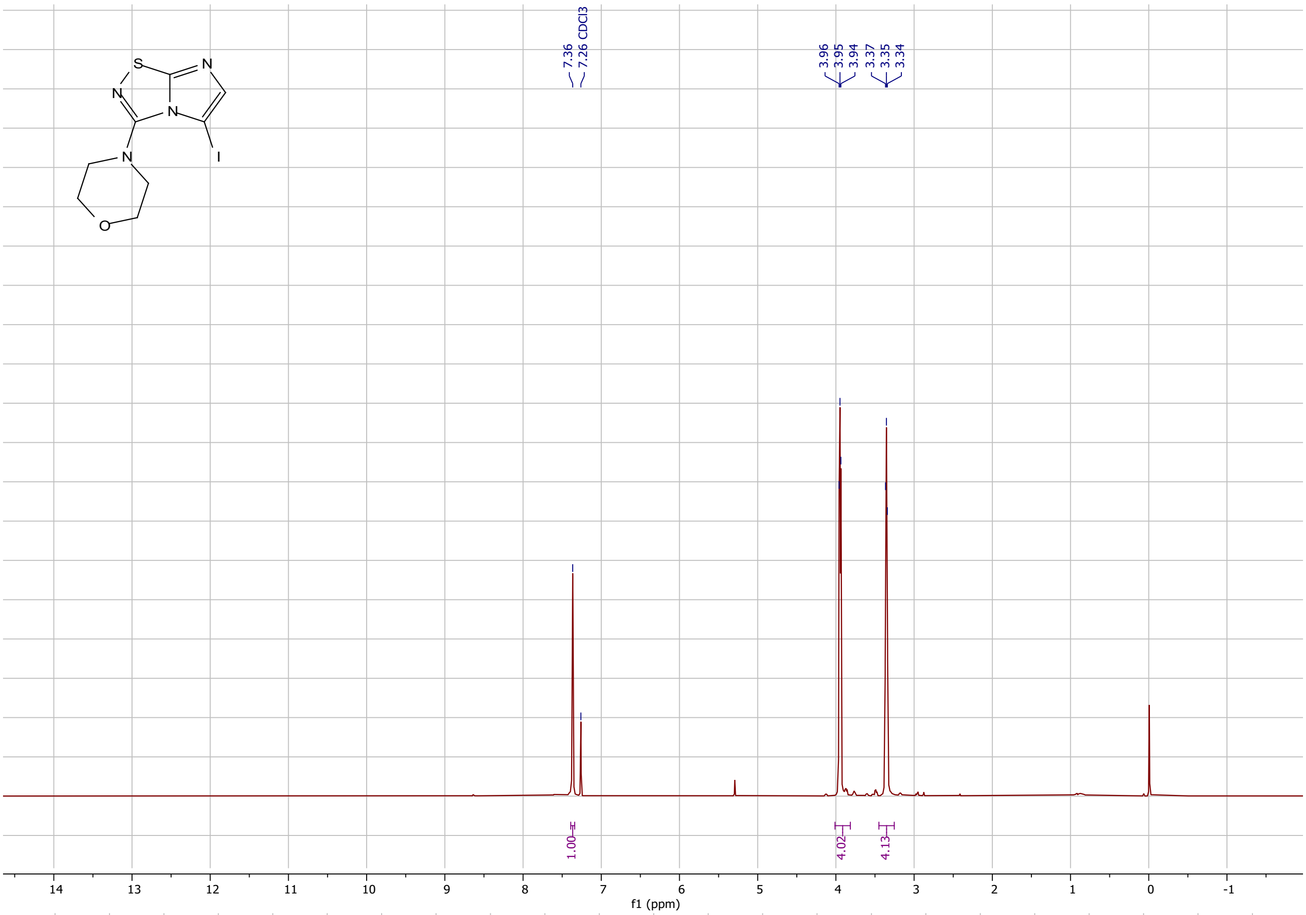

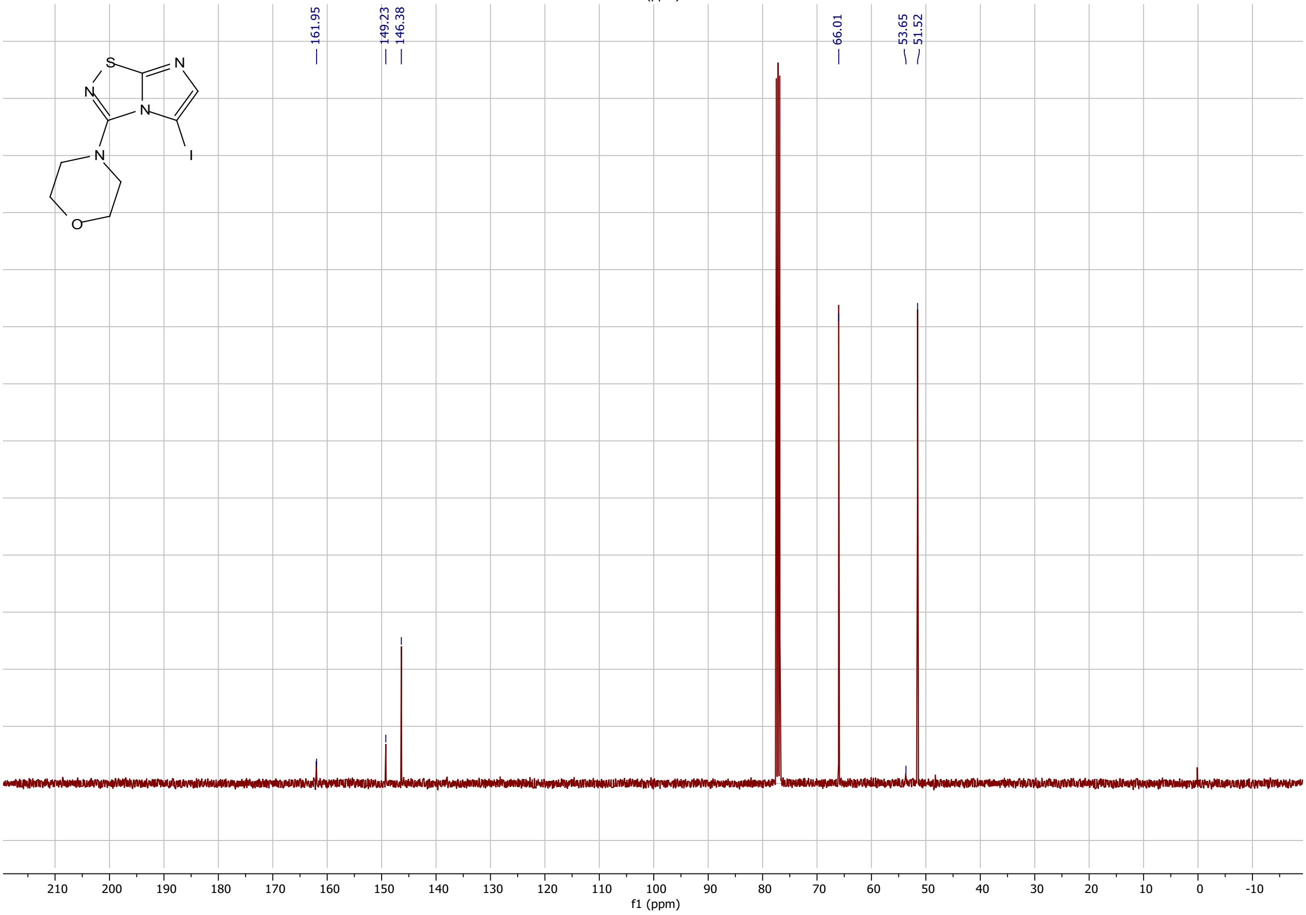

N-benzyl-5-iodoimidazo[1,2-*d*][1,2,4]thiadiazol-3-amine (23)

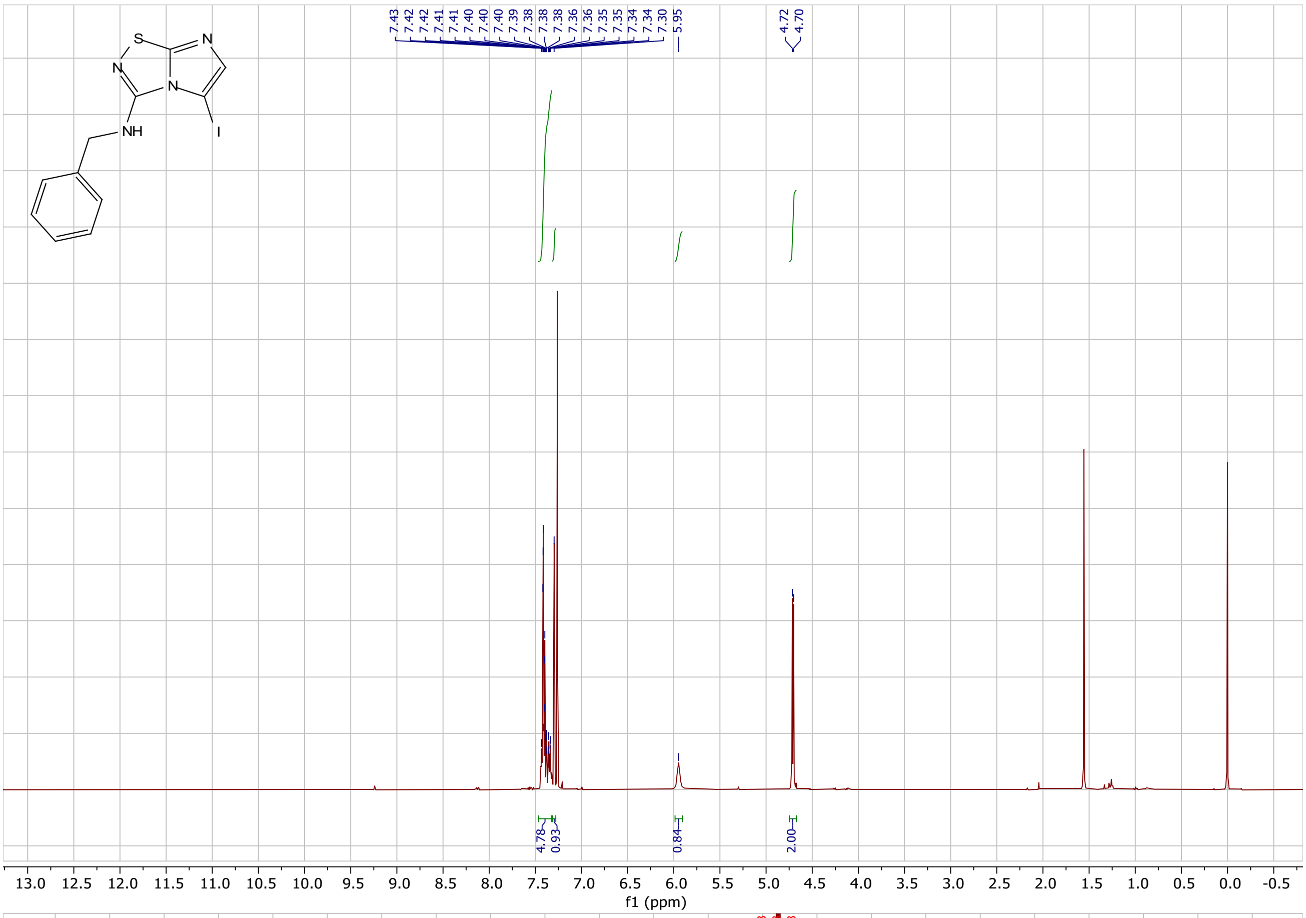

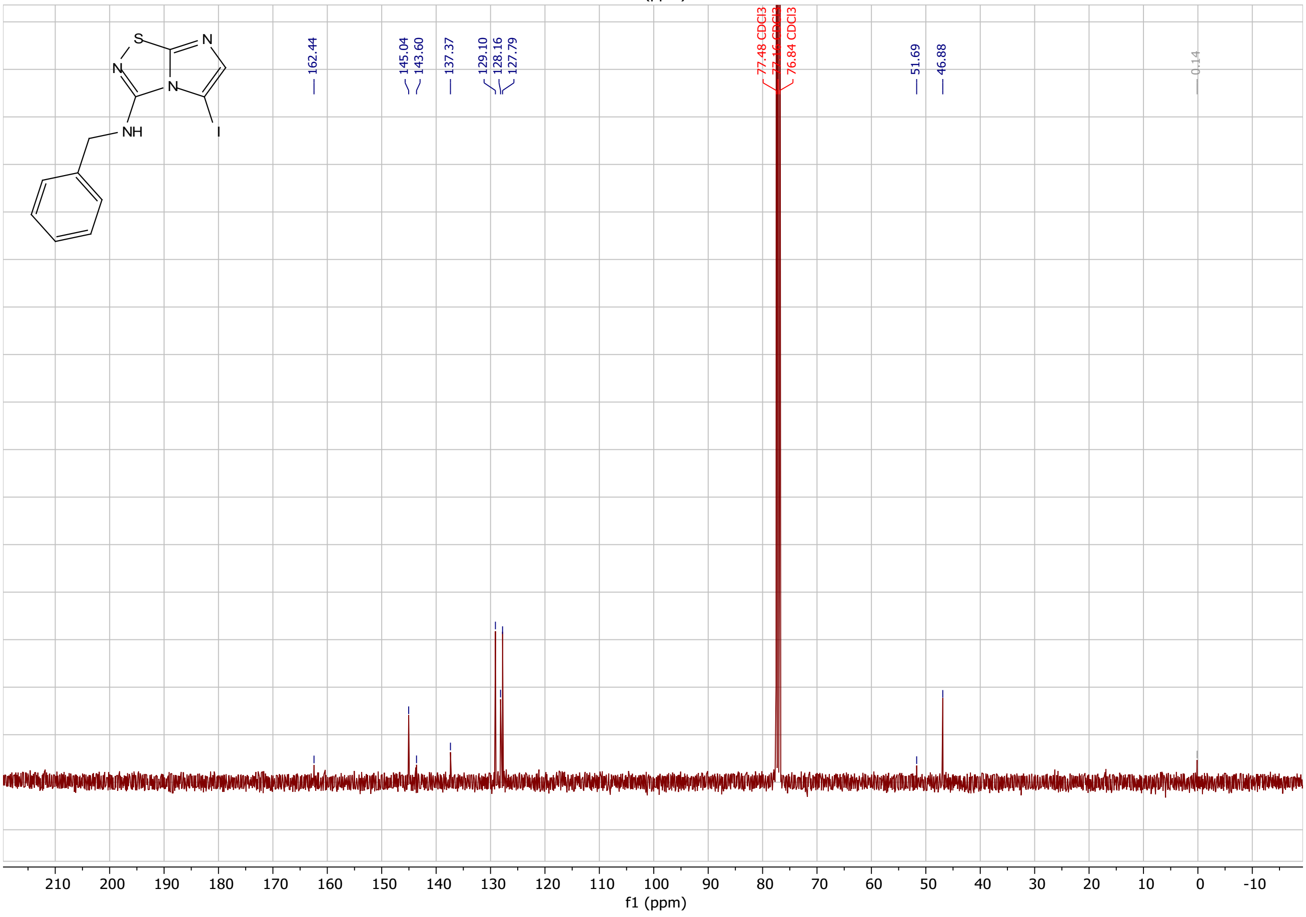

5-iodo-3-methoxyimidazo[1,2-*d*][1,2,4]thiadiazole (24)

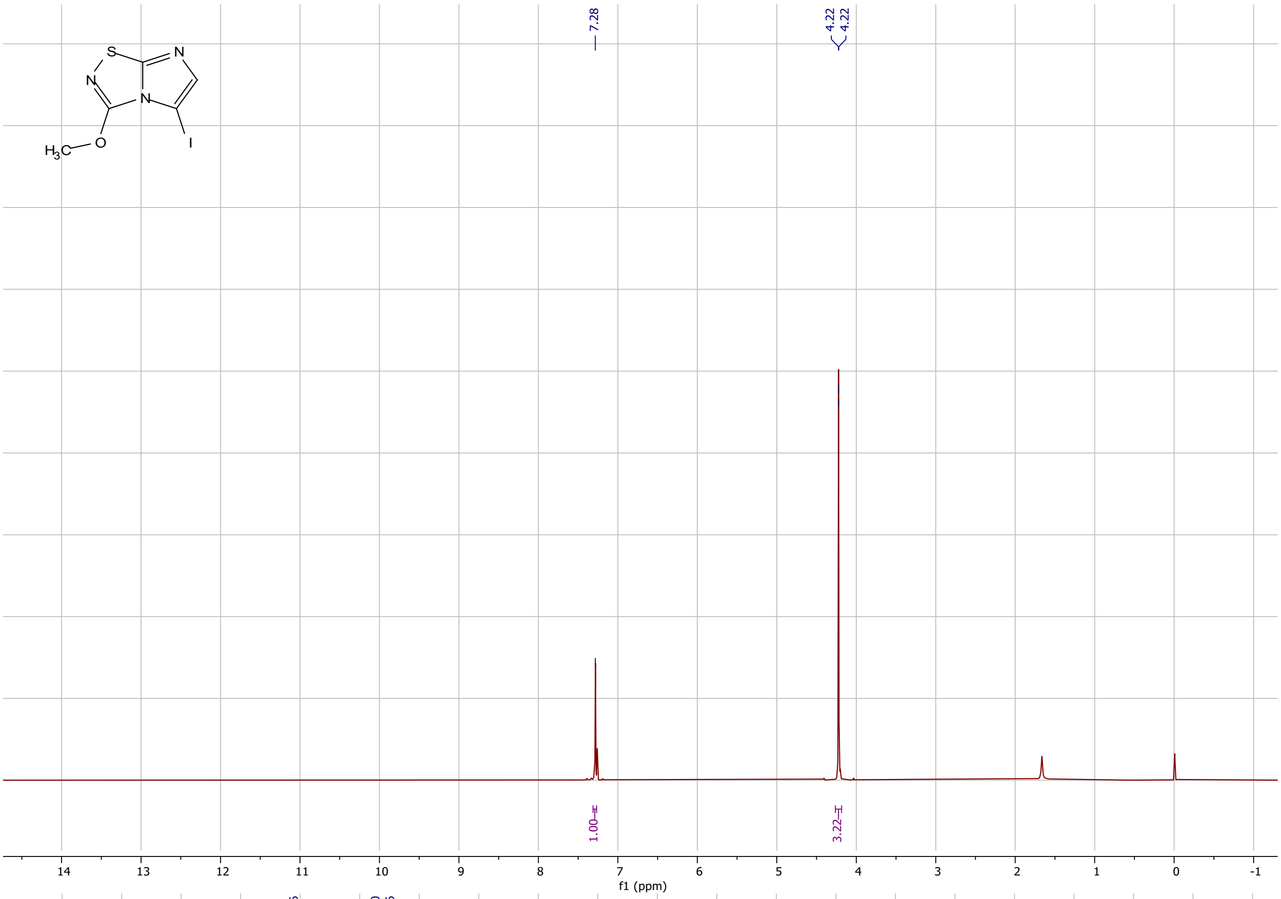

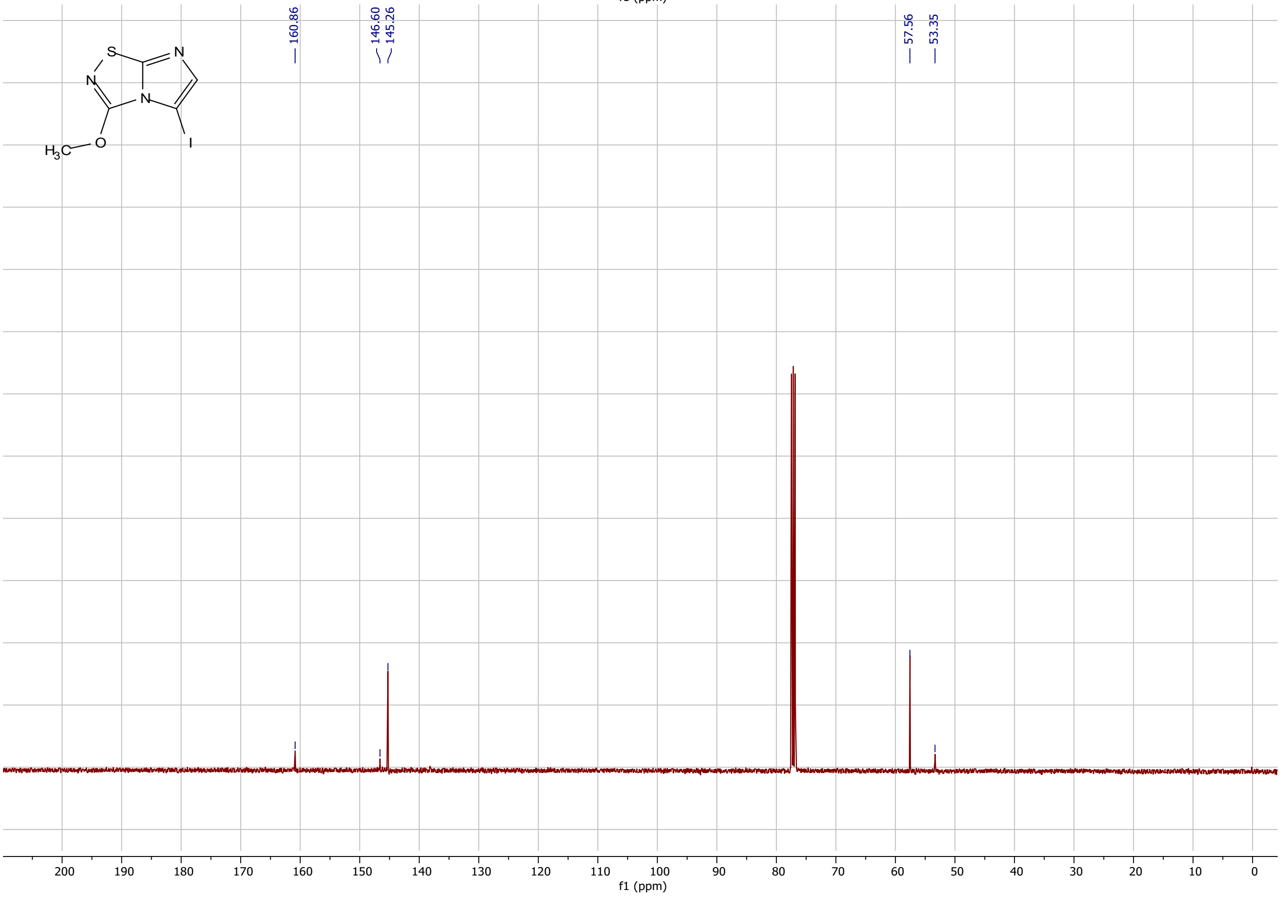

5-iodo-3-ethoxyimidazo[1,2-*d*][1,2,4]thiadiazole (25)

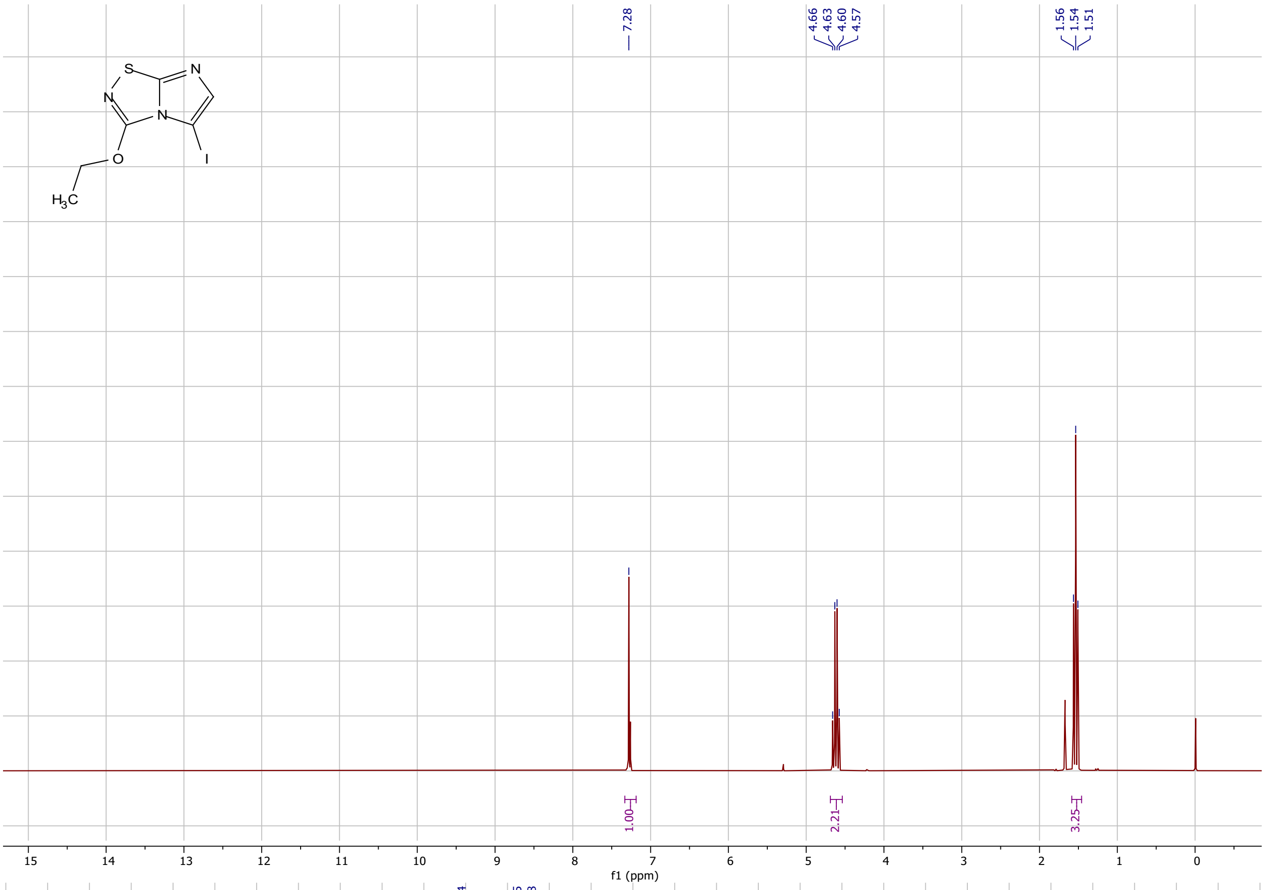

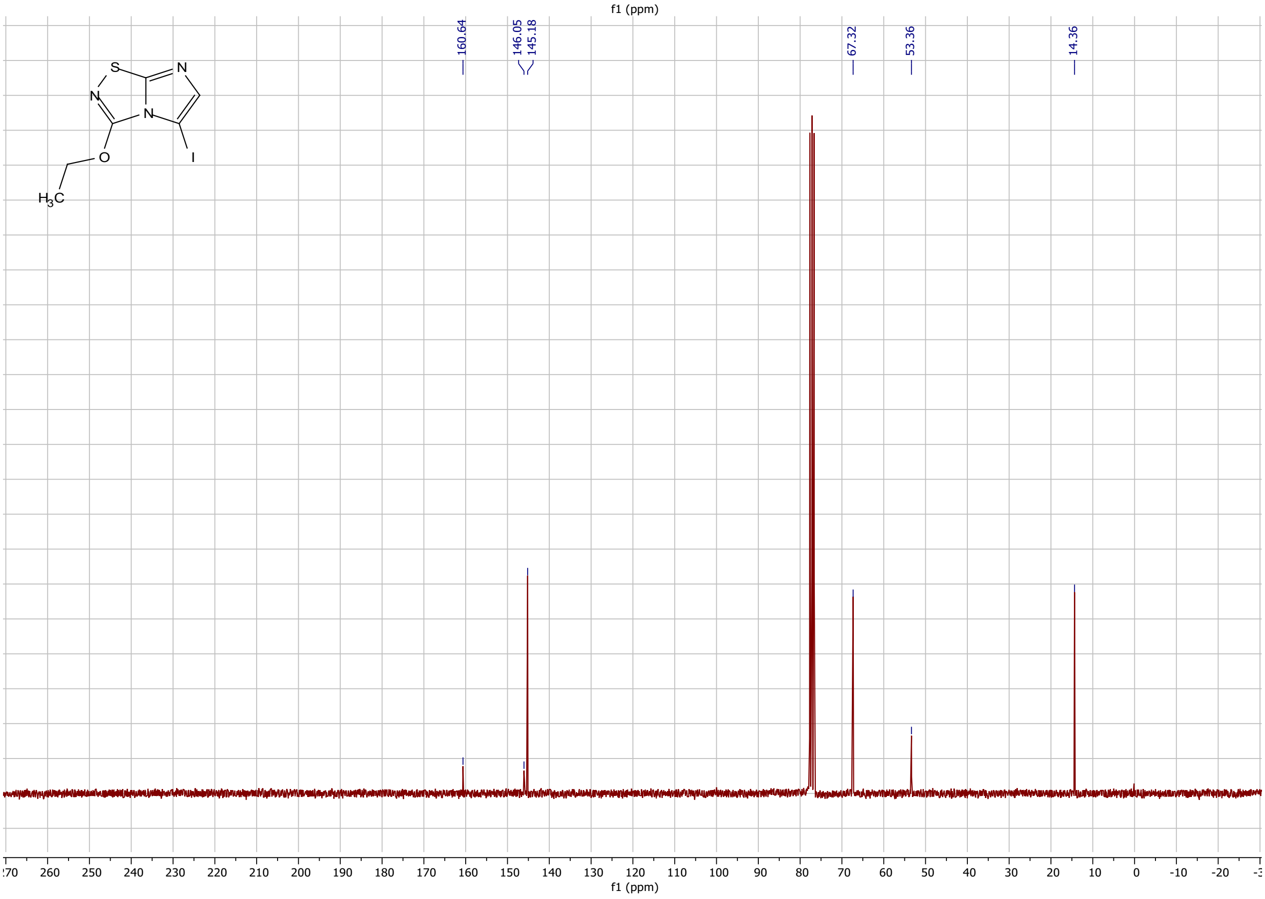

5-bromo-3-(morpholin-4-yl)imidazo[1,2-*d*][1,2,4]thiadiazole (26)

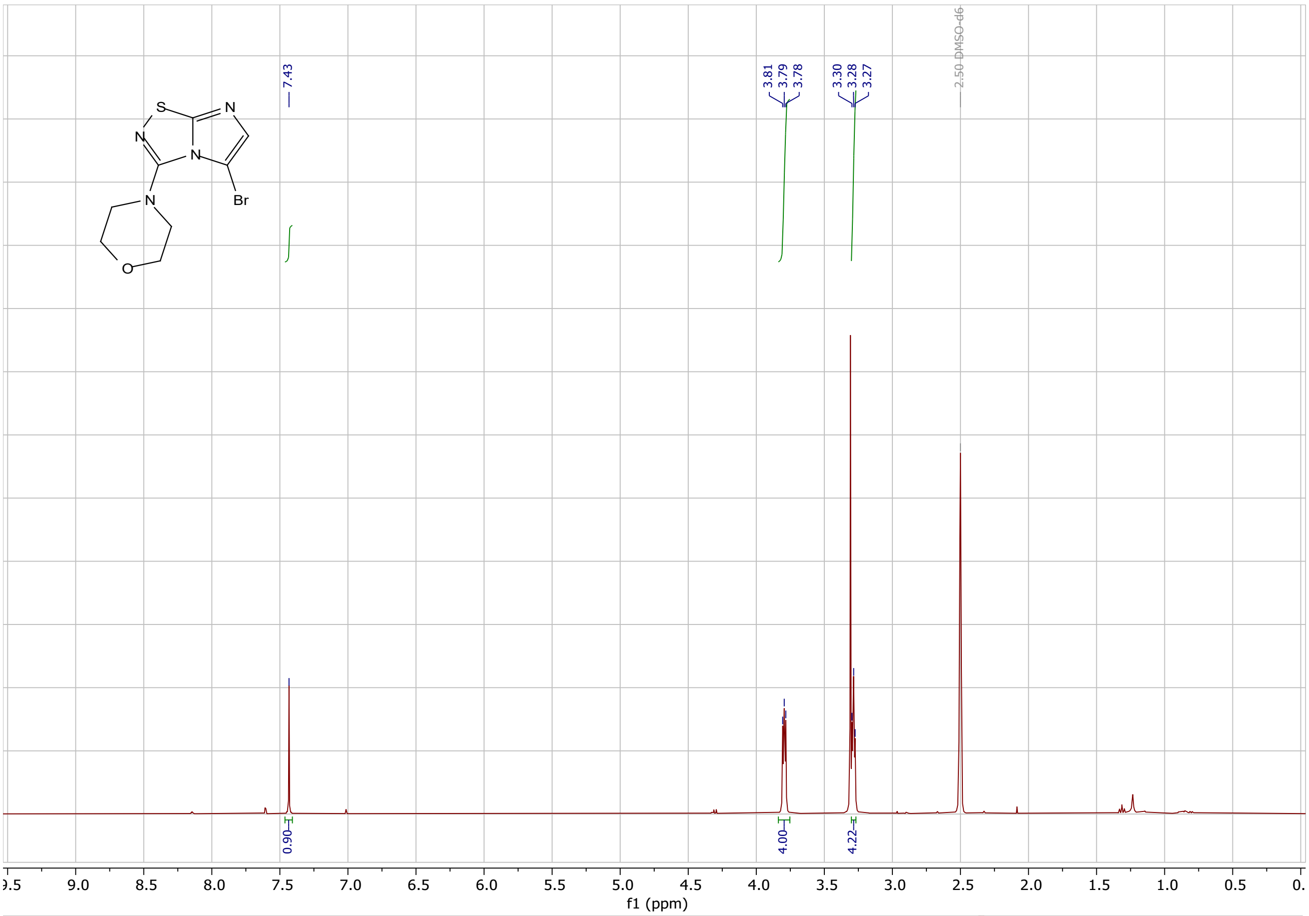

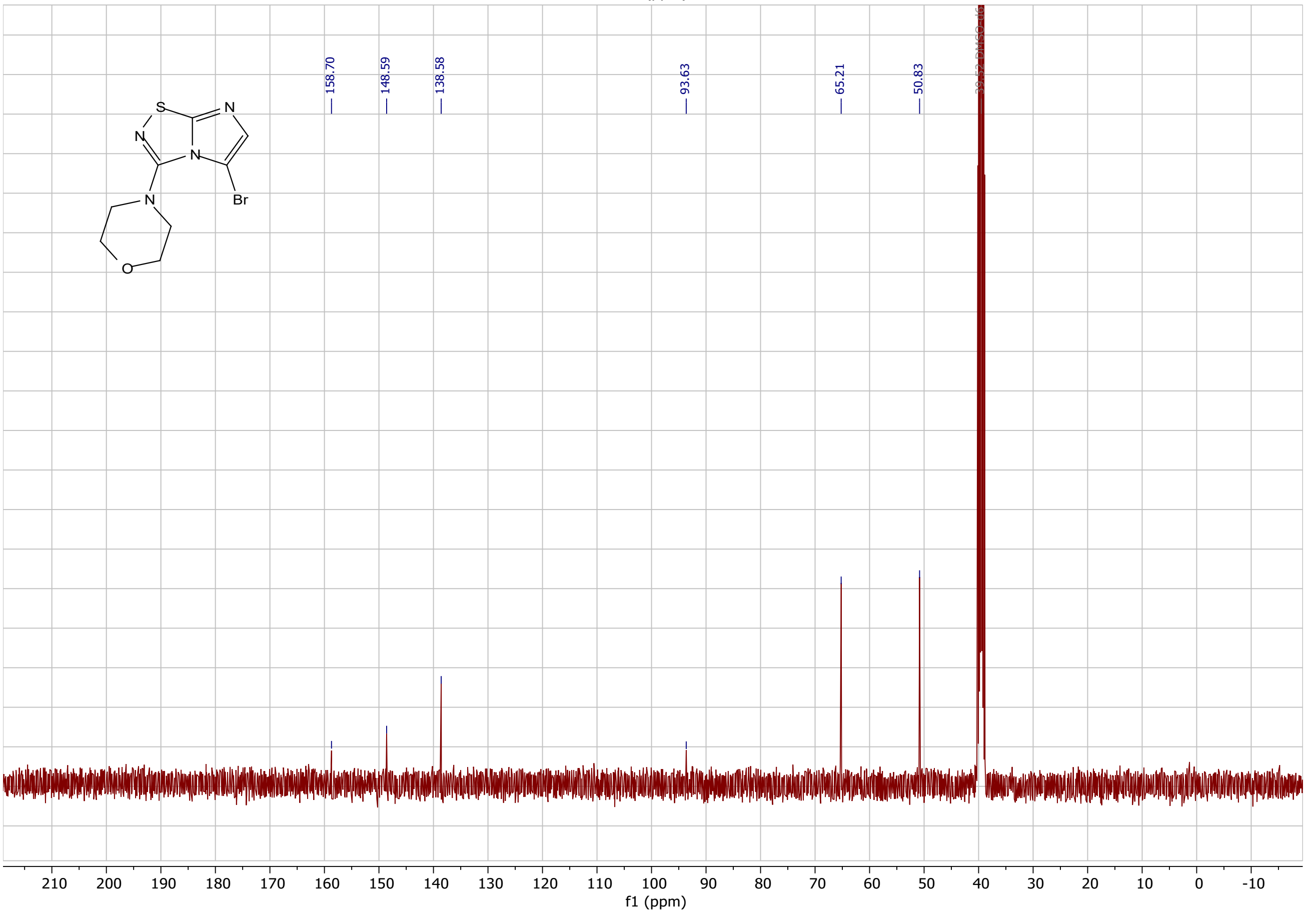

5-bromo-3-methoxy-imidazo[1,2-*d*][1,2,4]thiadiazole (27)

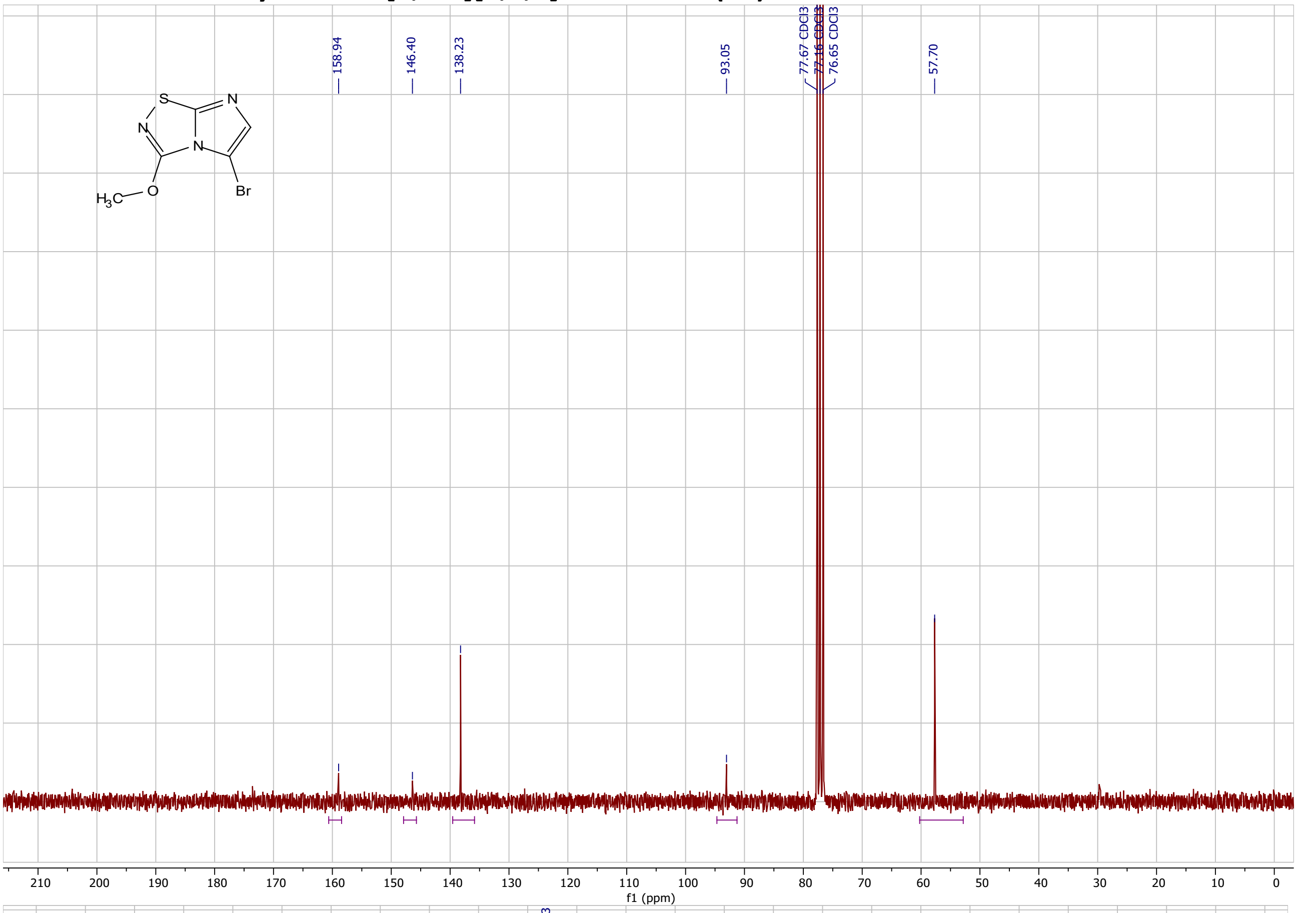

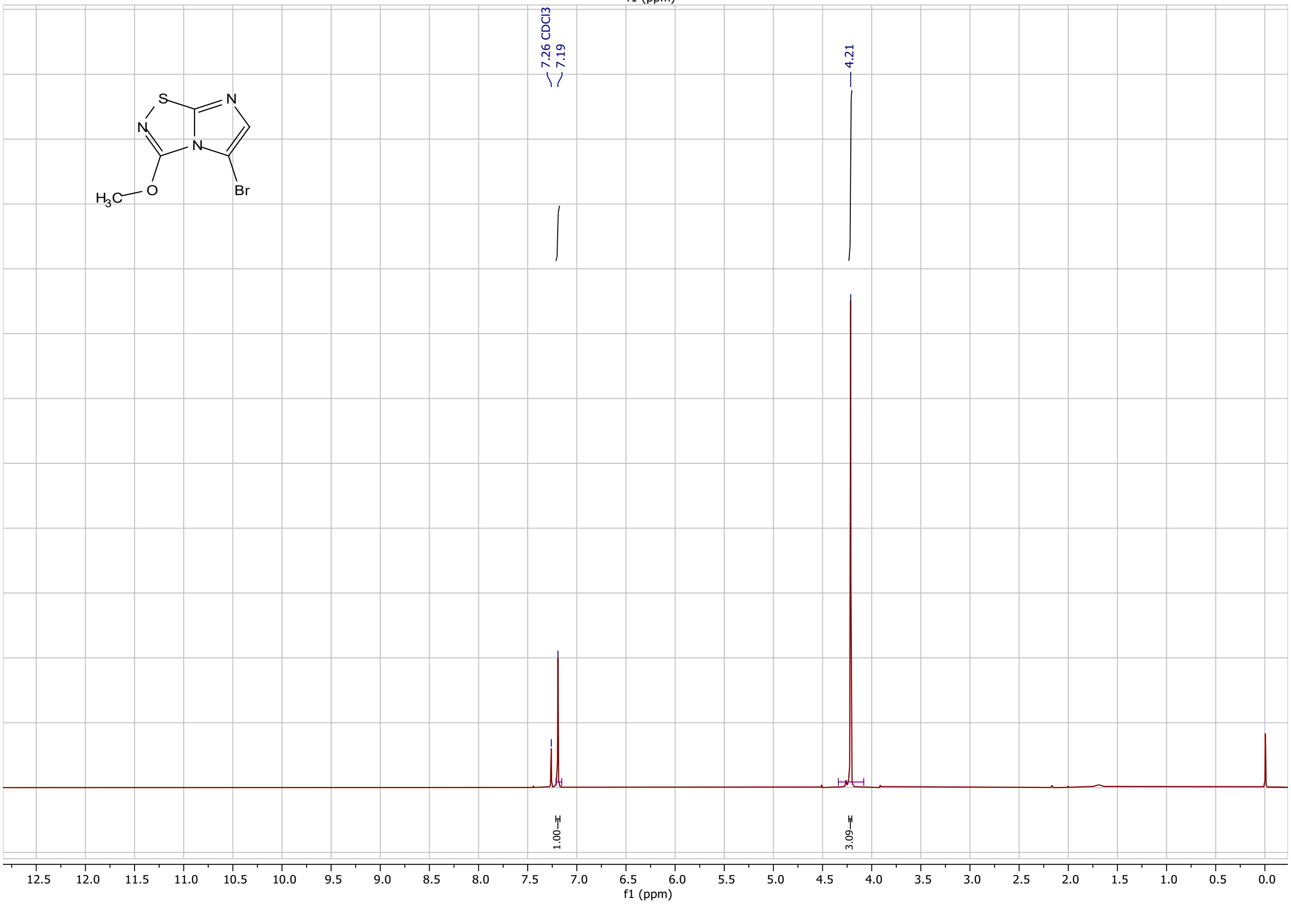

4-(5-(p-tolyl)imidazo[1,2-*d*][1,2,4]thiadiazol-3-yl)morpholine (28)

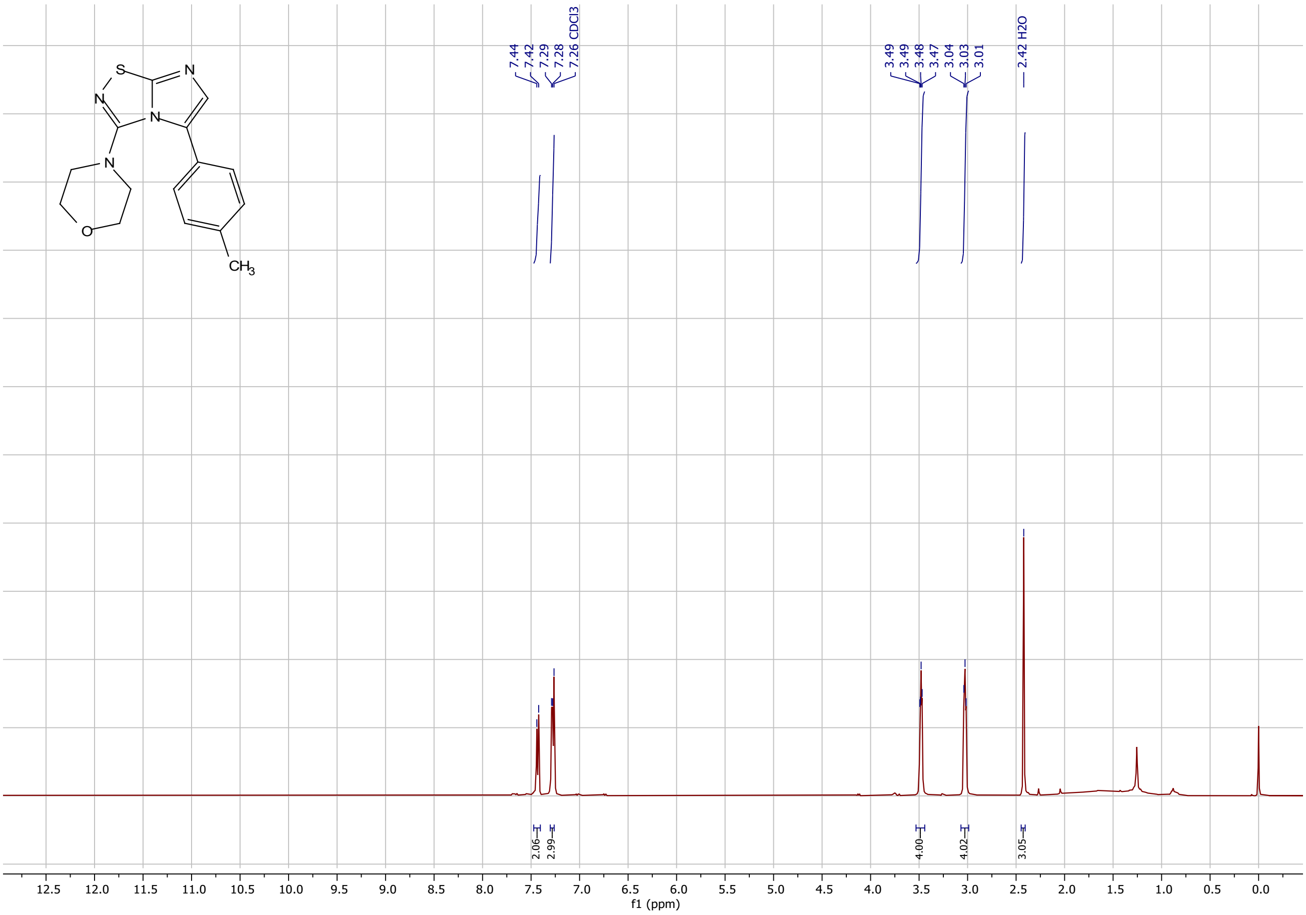

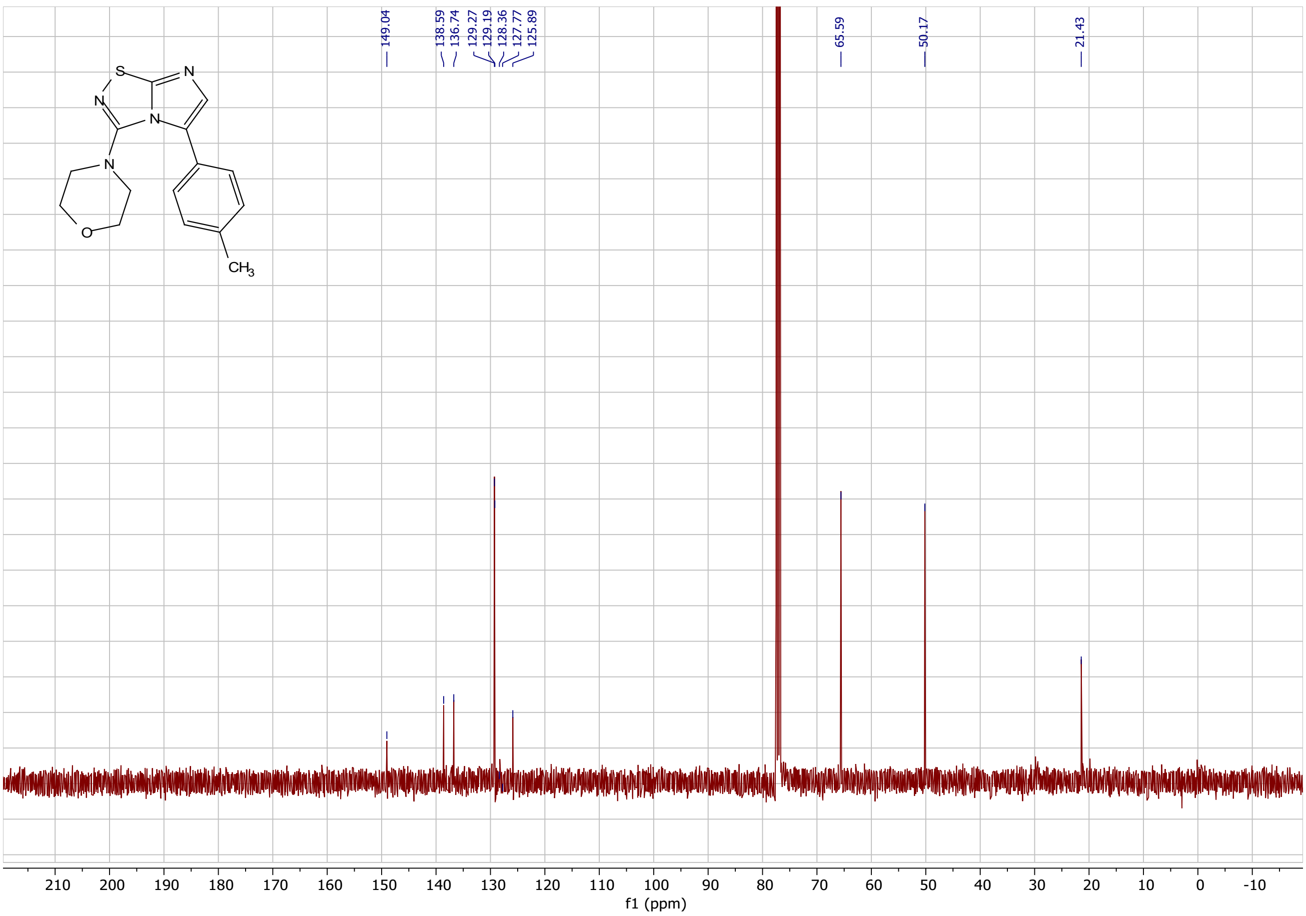

4-(5-phenylimidazo[1,2-*d*][1,2,4]thiadiazol-3-yl)morpholine (29)

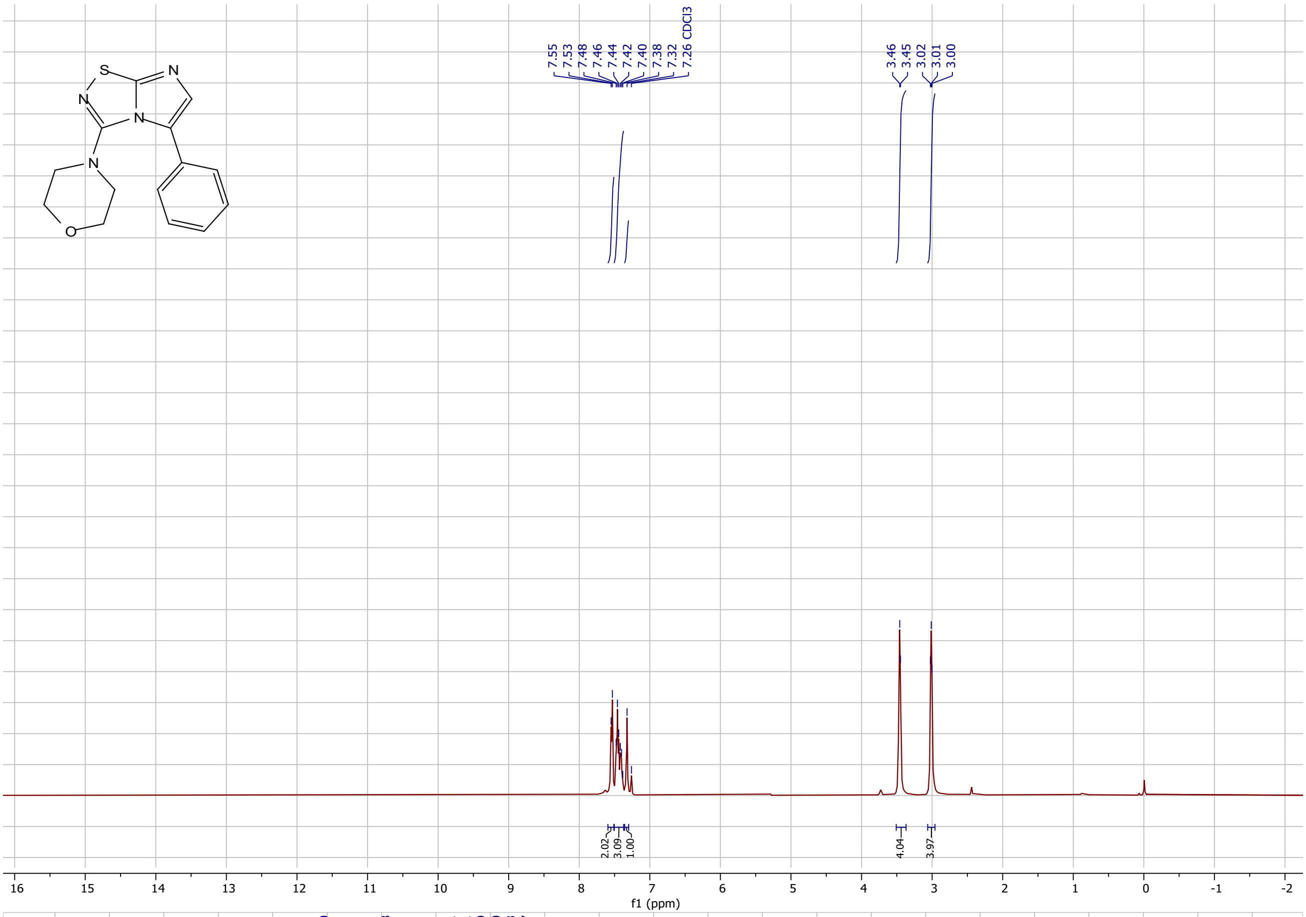

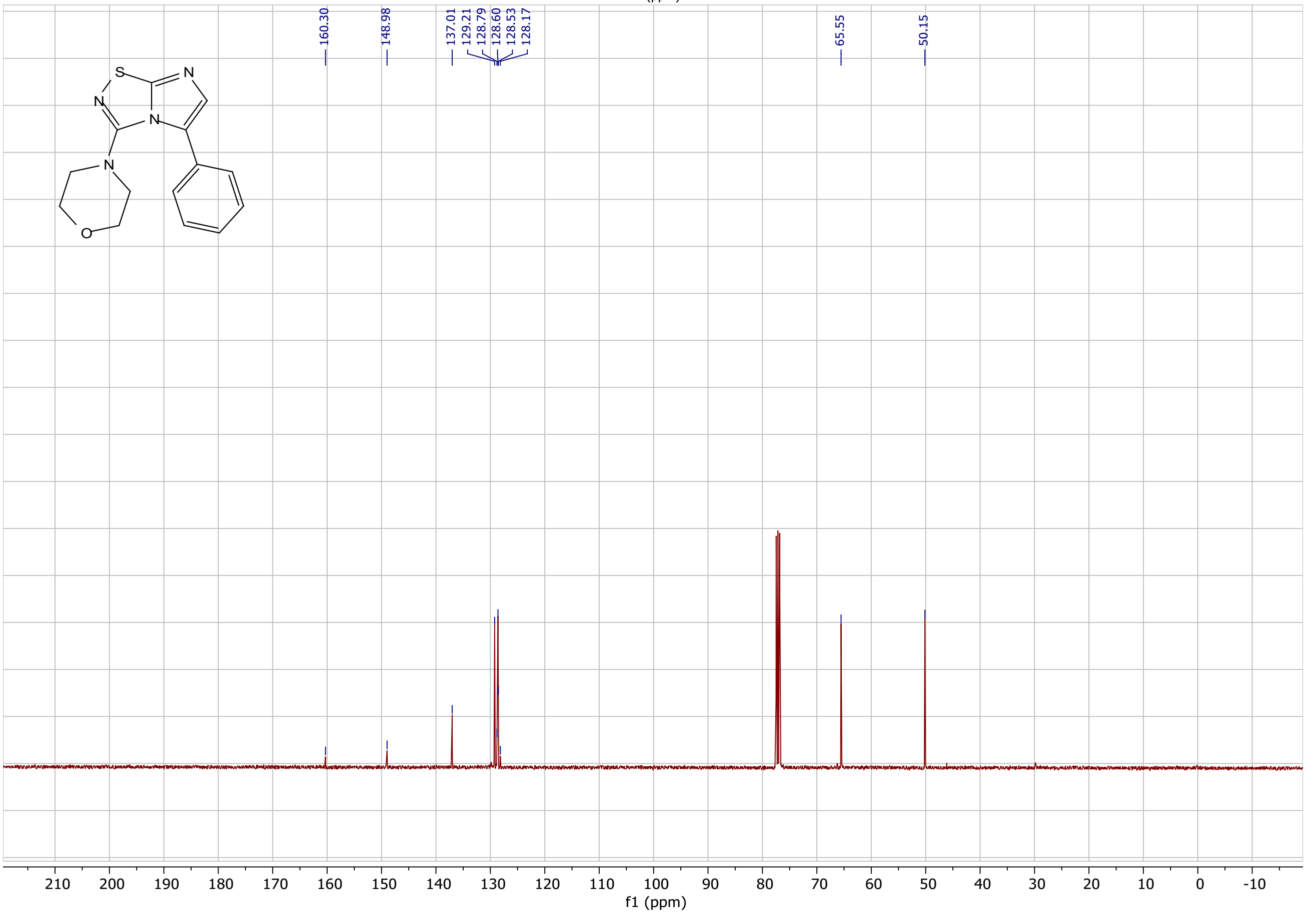

4-(5-(4-methoxyphenyl)imidazo[1,2-*d*][1,2,4]thiadiazol-3-yl)morpholine (30)

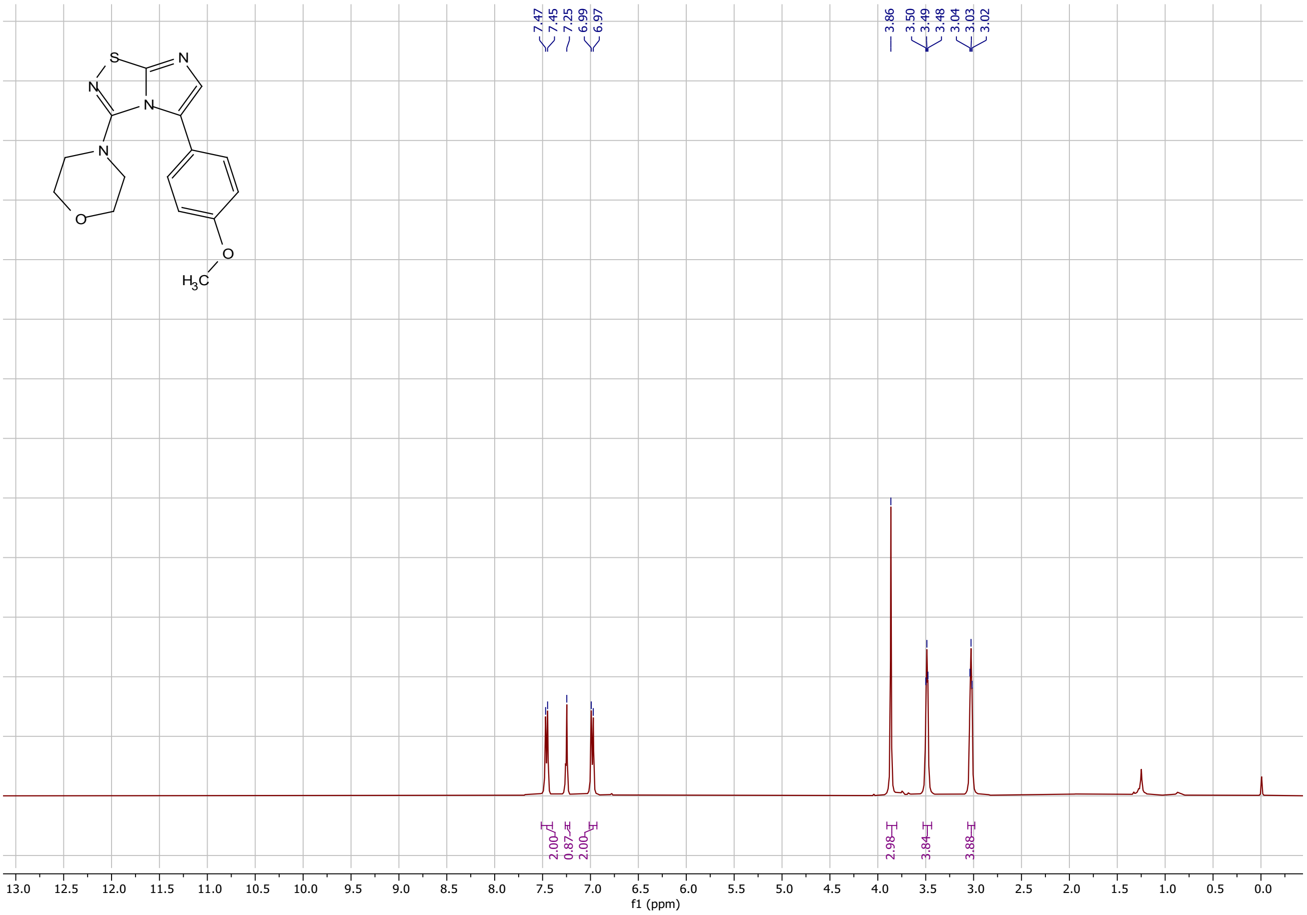
S
N
N
N
N
O
O
H3C
7.47
7.45
7.25
6.99
6.97
3.86
3.50
3.49
3.48
3.04
3.03
3.02
2.00
0.87
2.00
2.98
3.84
3.88
f1 (ppm)

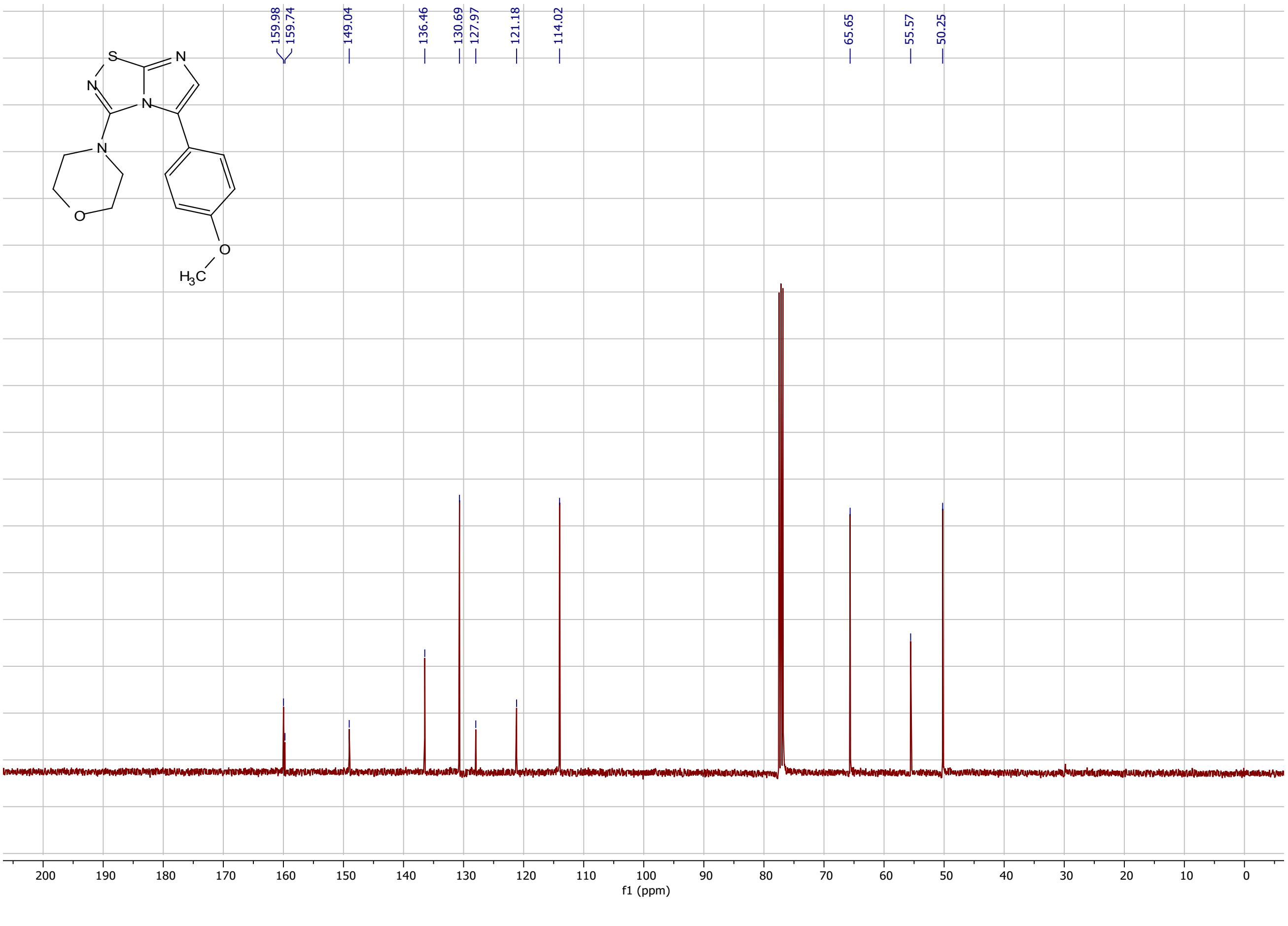
S
N
N
N
N
O
O
H3C
159.98
159.74
149.04
136.46
130.69
127.97
121.18
114.02
65.65
55.57
50.25
f1 (ppm)

4-(5-(3-methoxyphenyl)imidazo[1,2-*d*][1,2,4]thiadiazol-3-yl)morpholine (31)

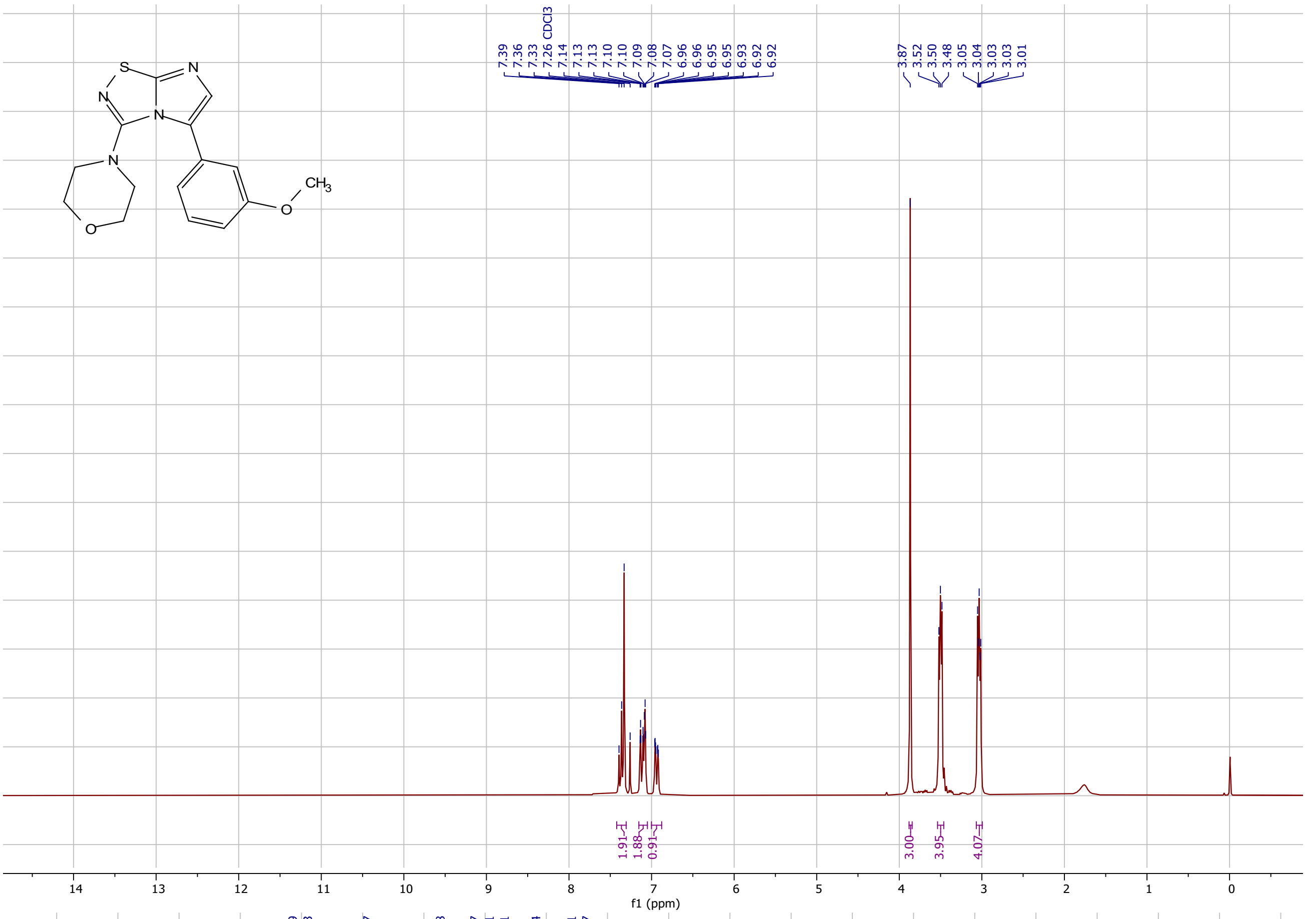

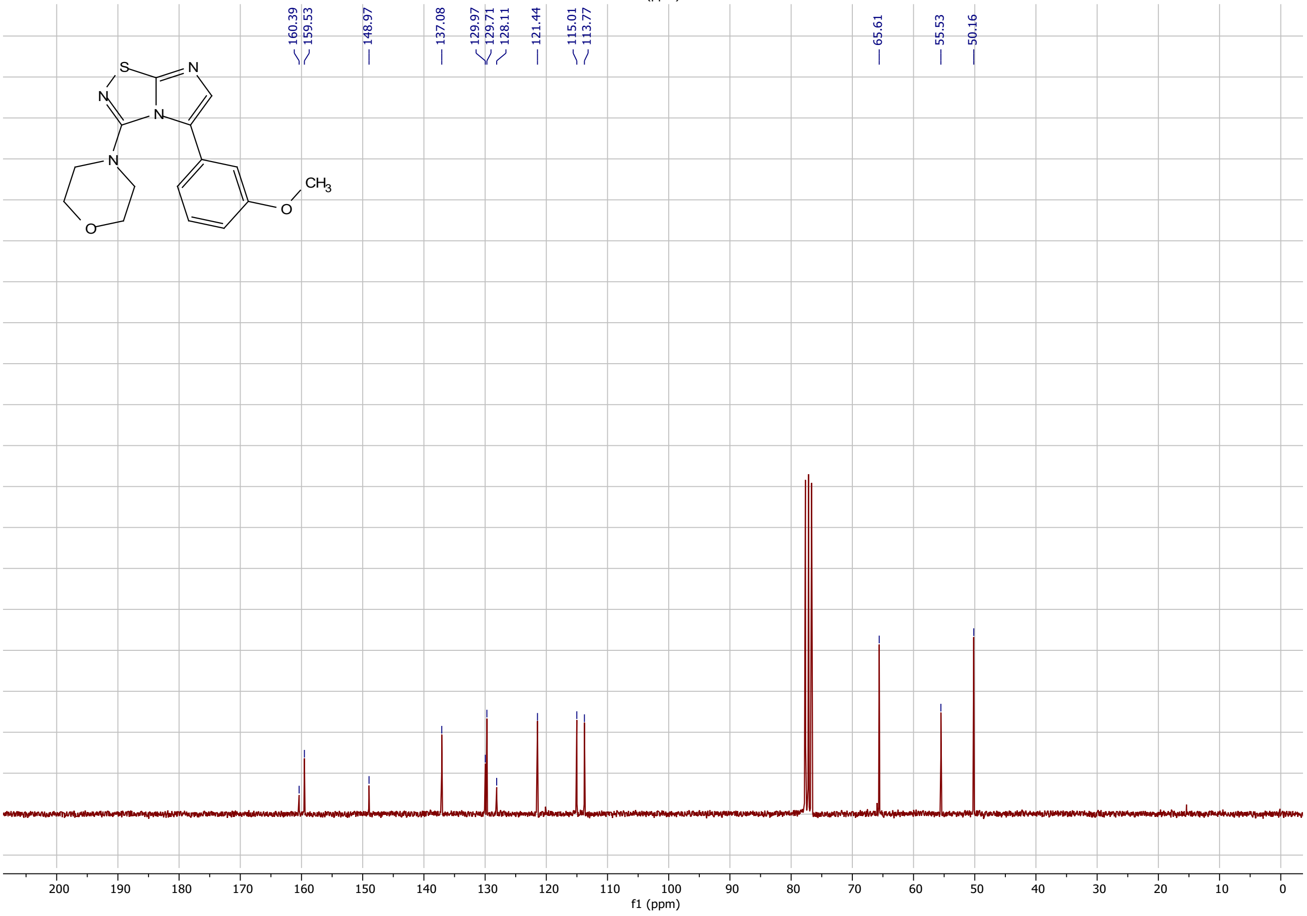

4-(5-(2-methoxyphenyl)imidazo[1,2-*d*][1,2,4]thiadiazol-3-yl)morpholine (32)

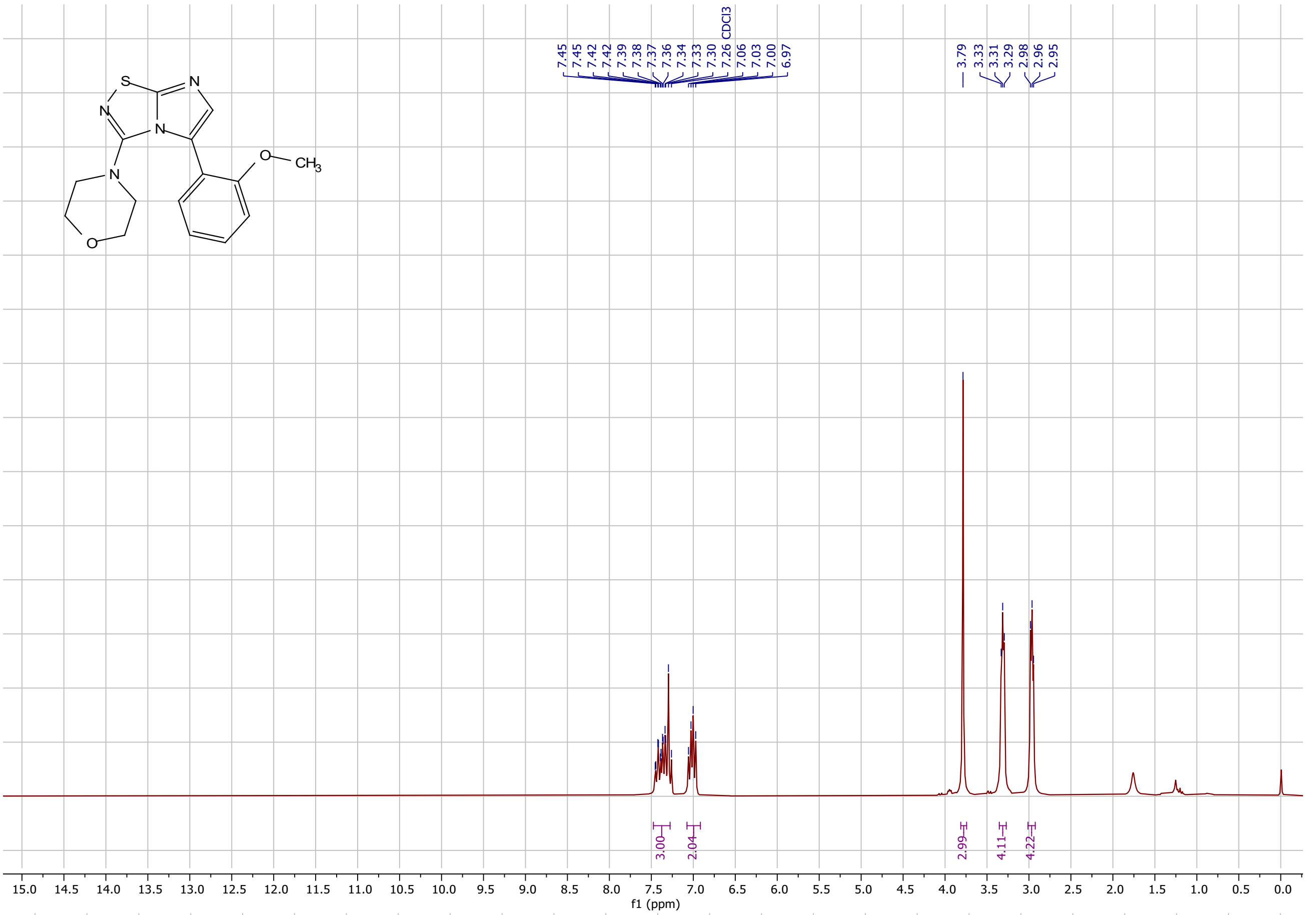

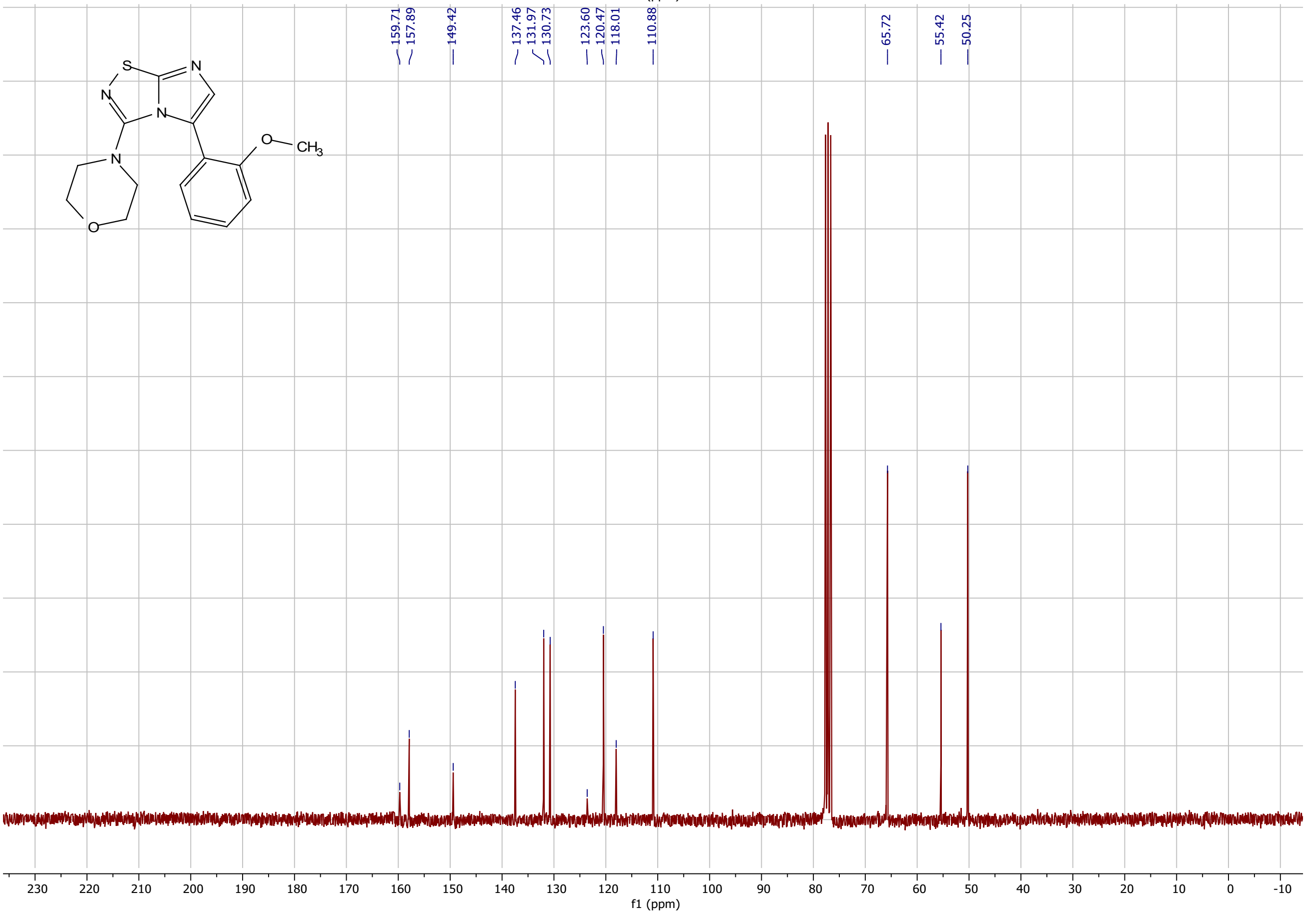

4-(3-morpholinoimidazo[1,2-*d*][1,2,4]thiadiazol-5-yl)phenol (33)

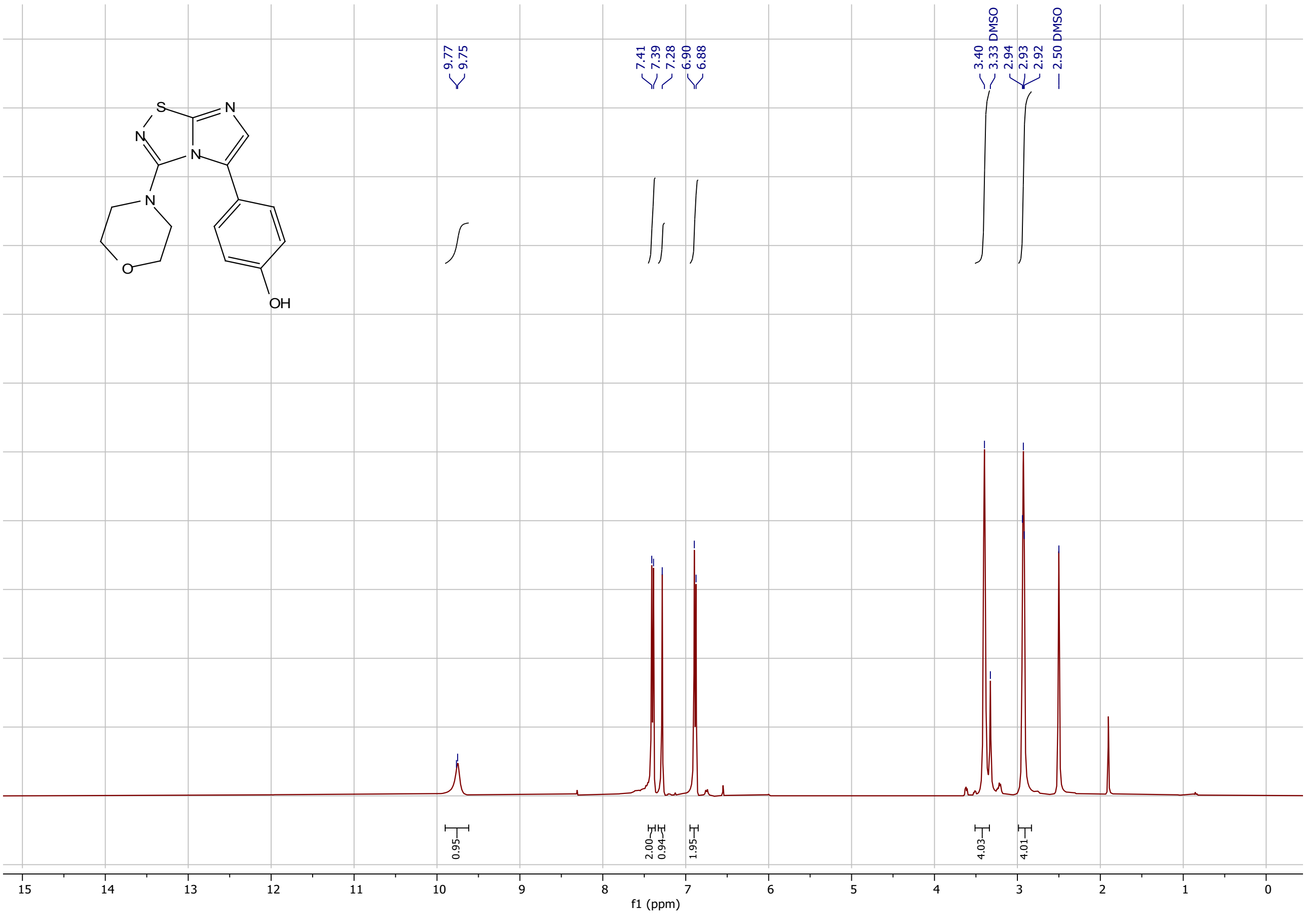

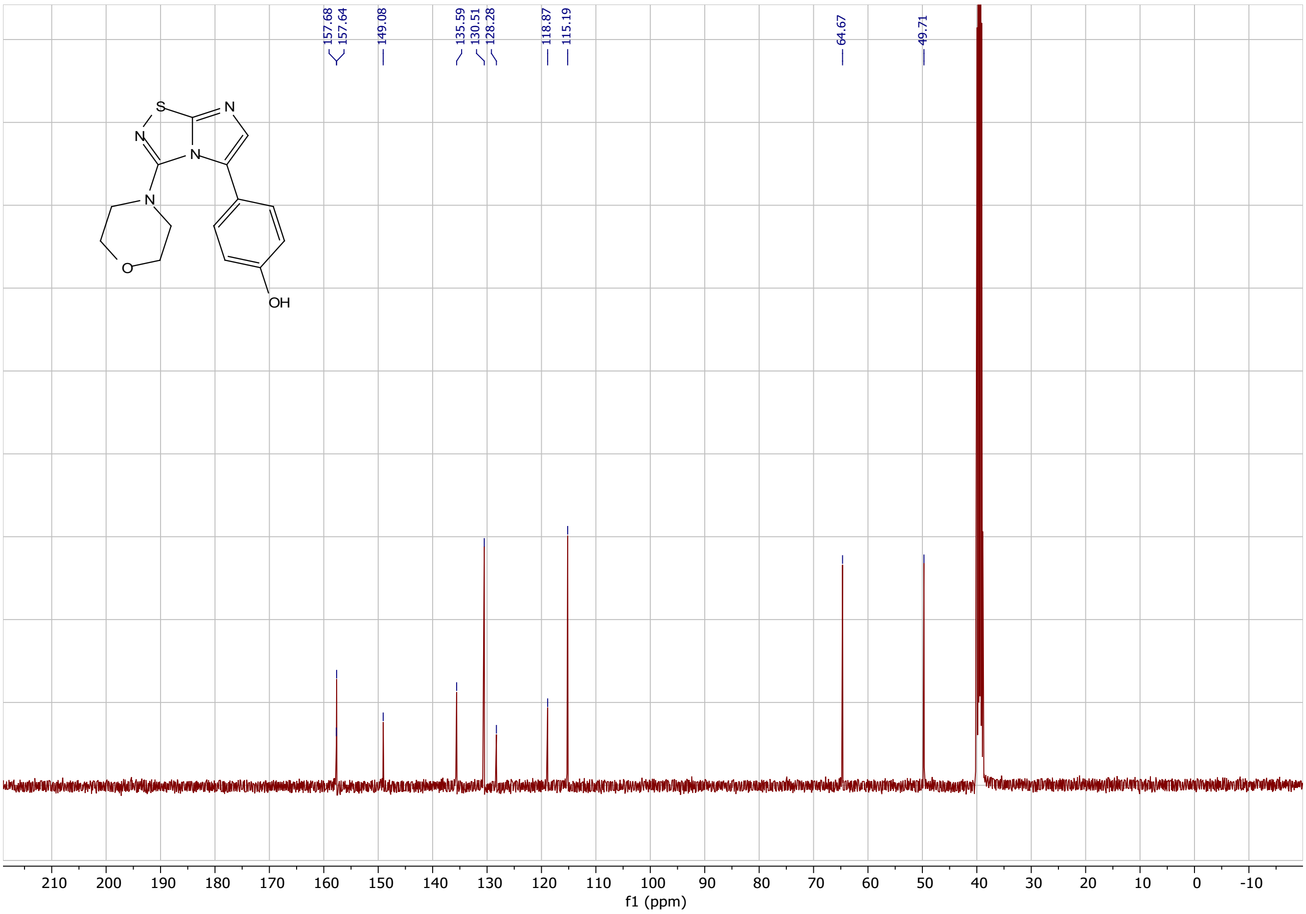

4-(5-(4-nitrophenyl)imidazo[1,2-*d*][1,2,4]thiadiazol-3-yl)morpholine (34)

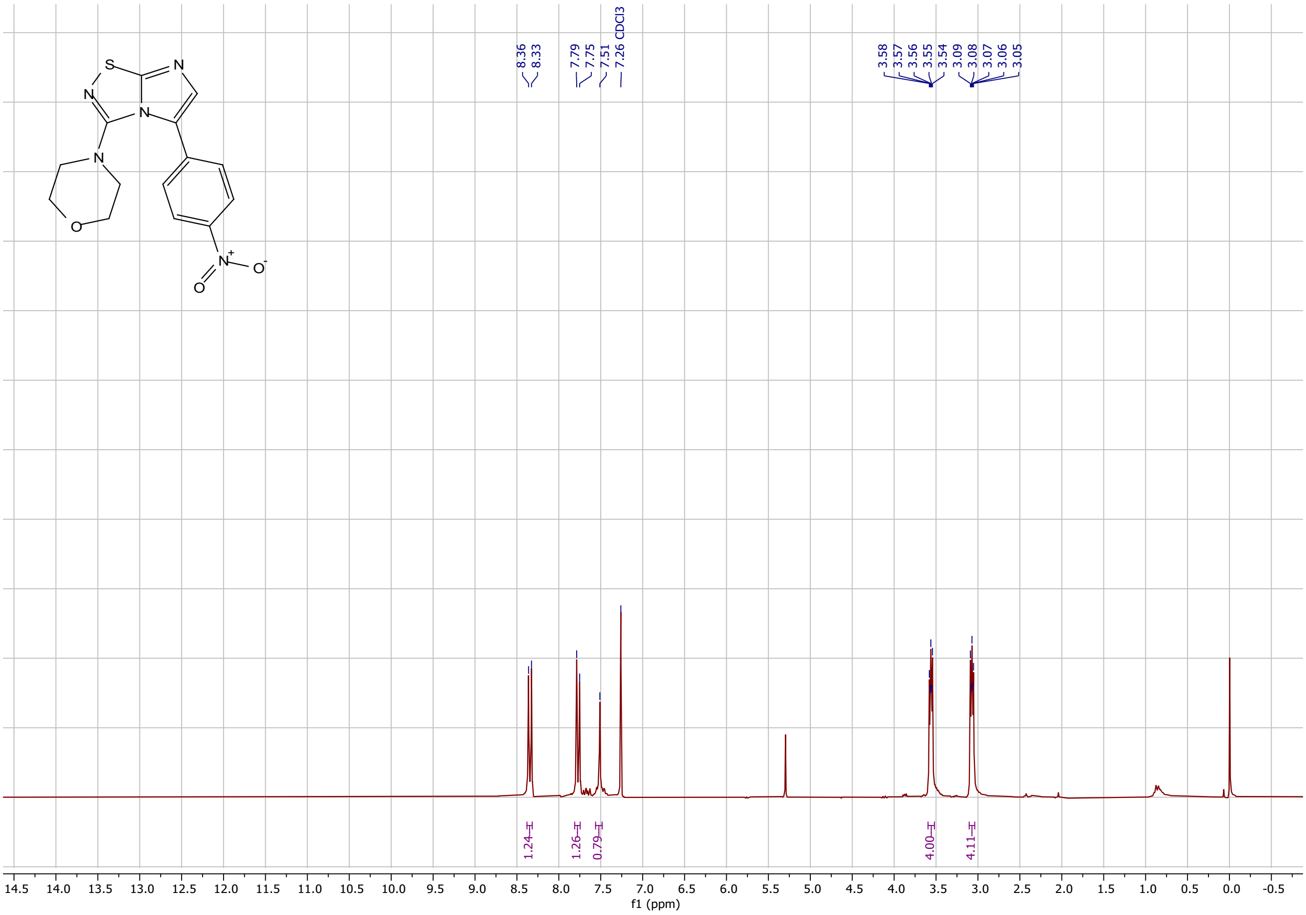
8.36
8.33
7.79
7.75
7.51
7.26 CDCl3
3.58
3.57
3.56
3.55
3.54
3.09
3.08
3.07
3.06
3.05
1.24
1.26
0.79
4.00
4.11
f1 (ppm)

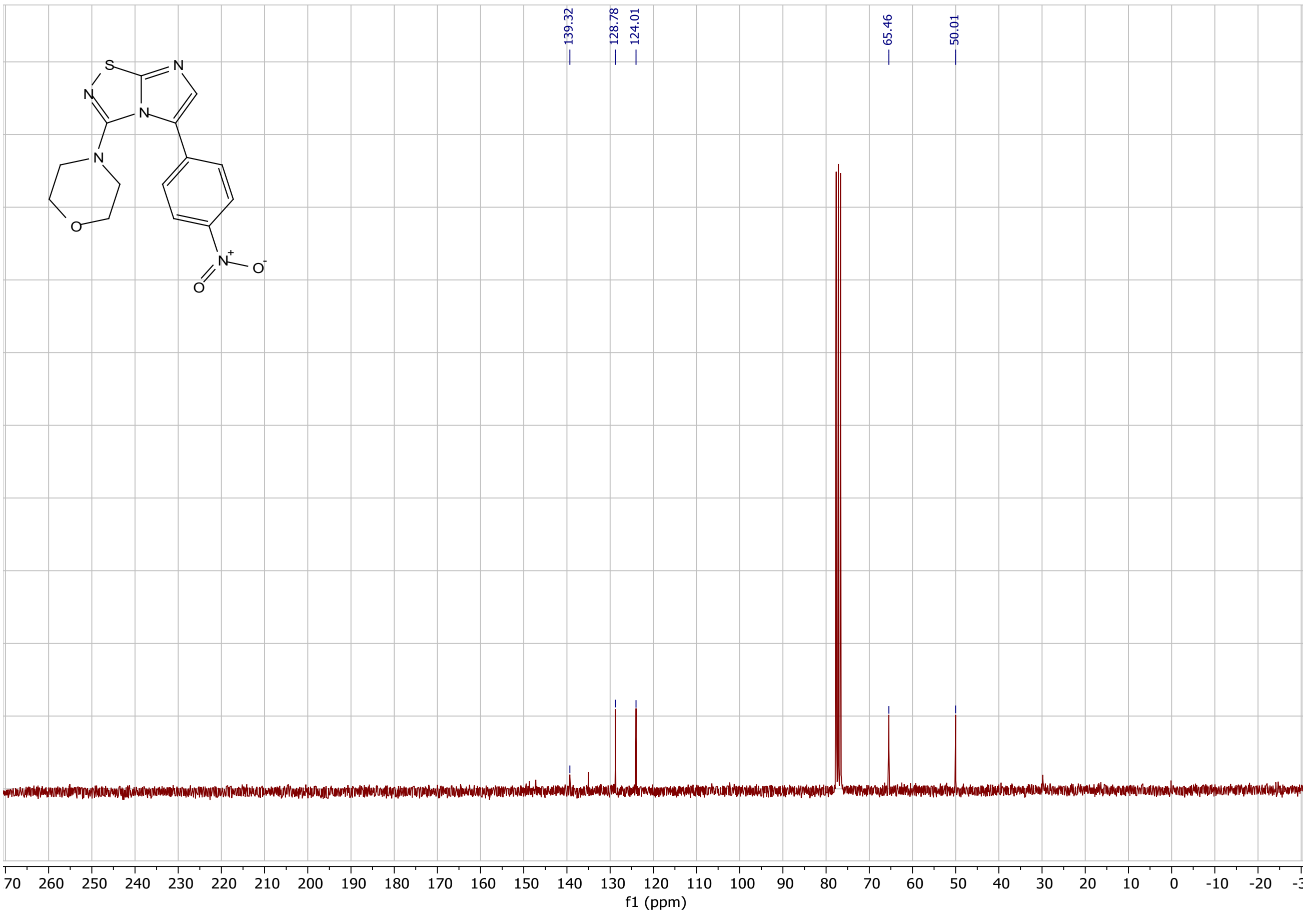
139.32
128.78
124.01
65.46
50.01
f1 (ppm)

4-(5-(4-fluorophenyl)imidazo[1,2-*d*][1,2,4]thiadiazol-3-yl)morpholine (35)

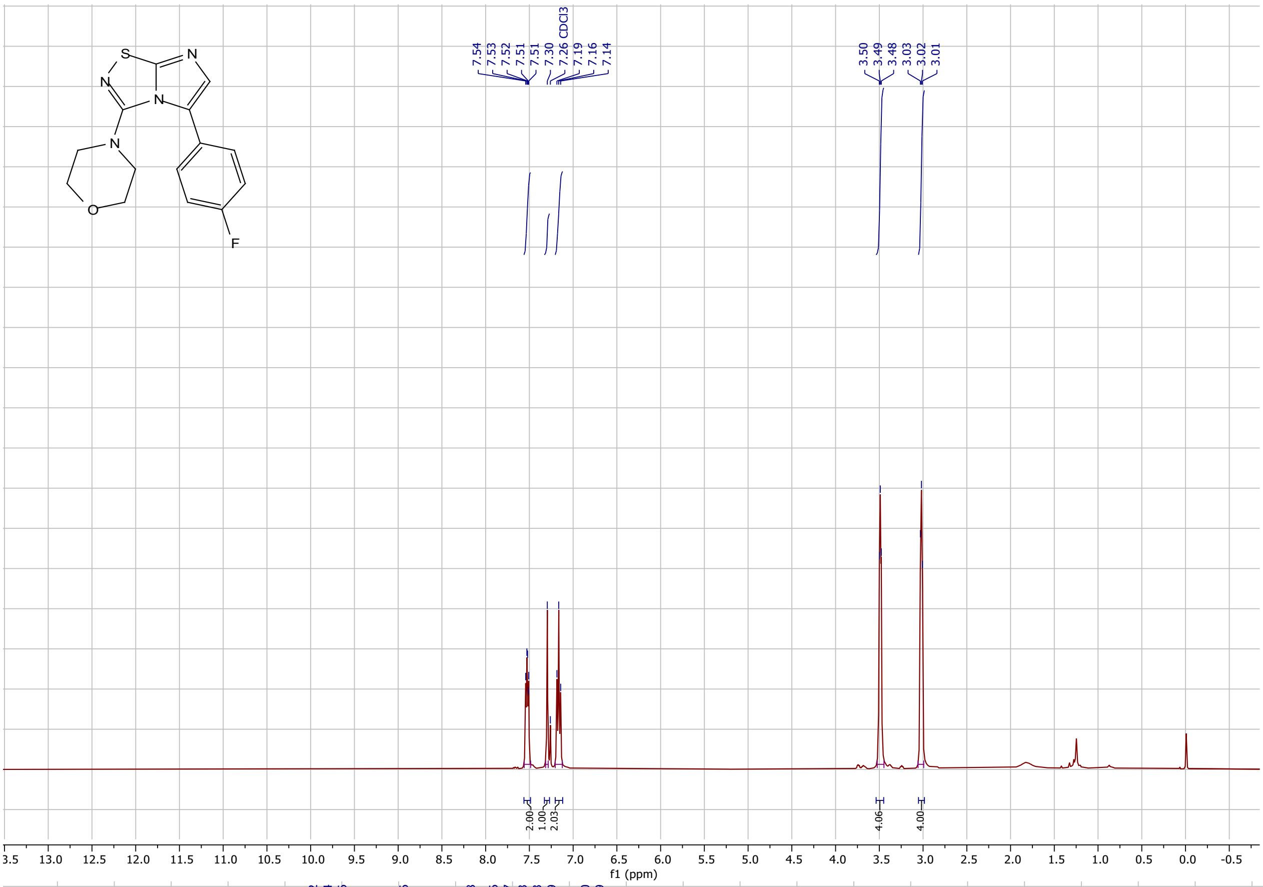

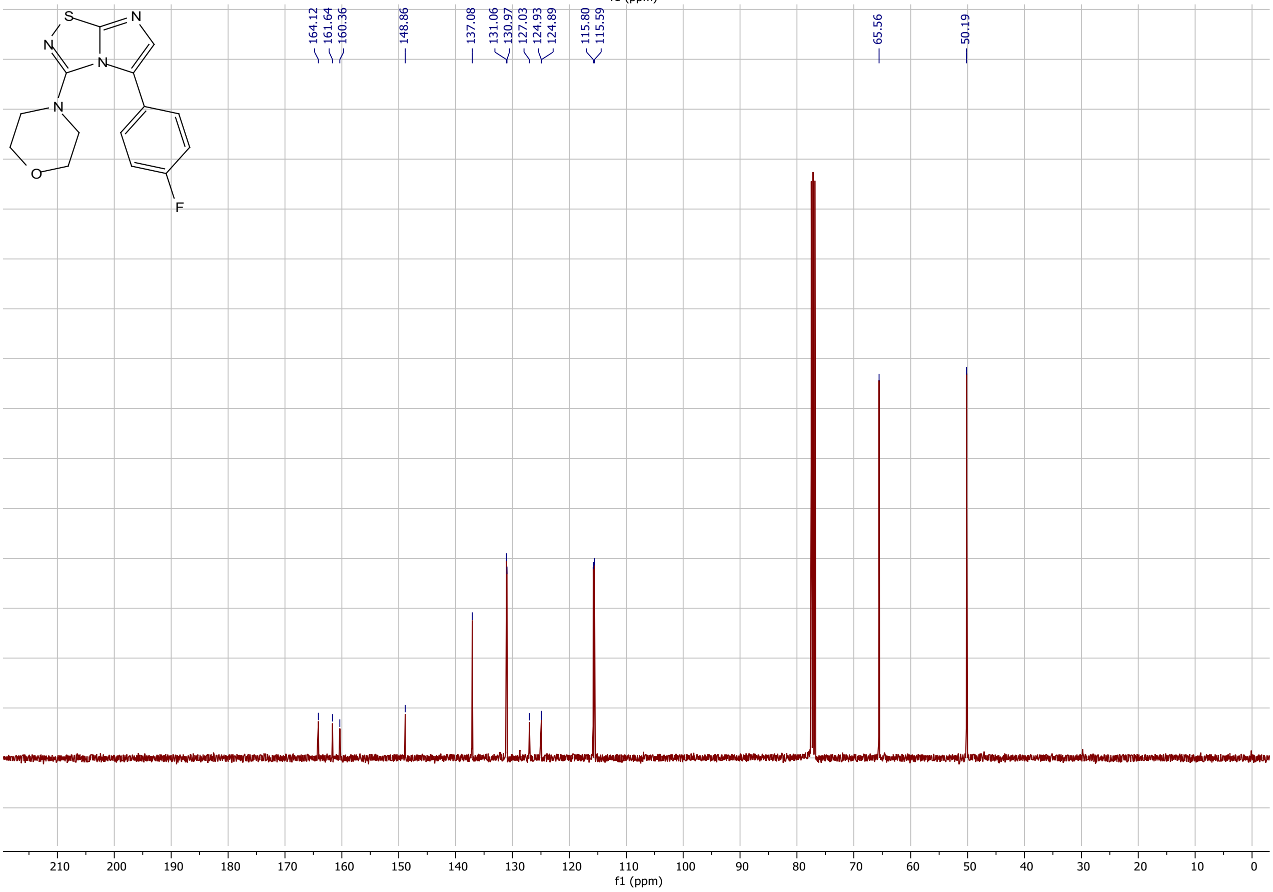

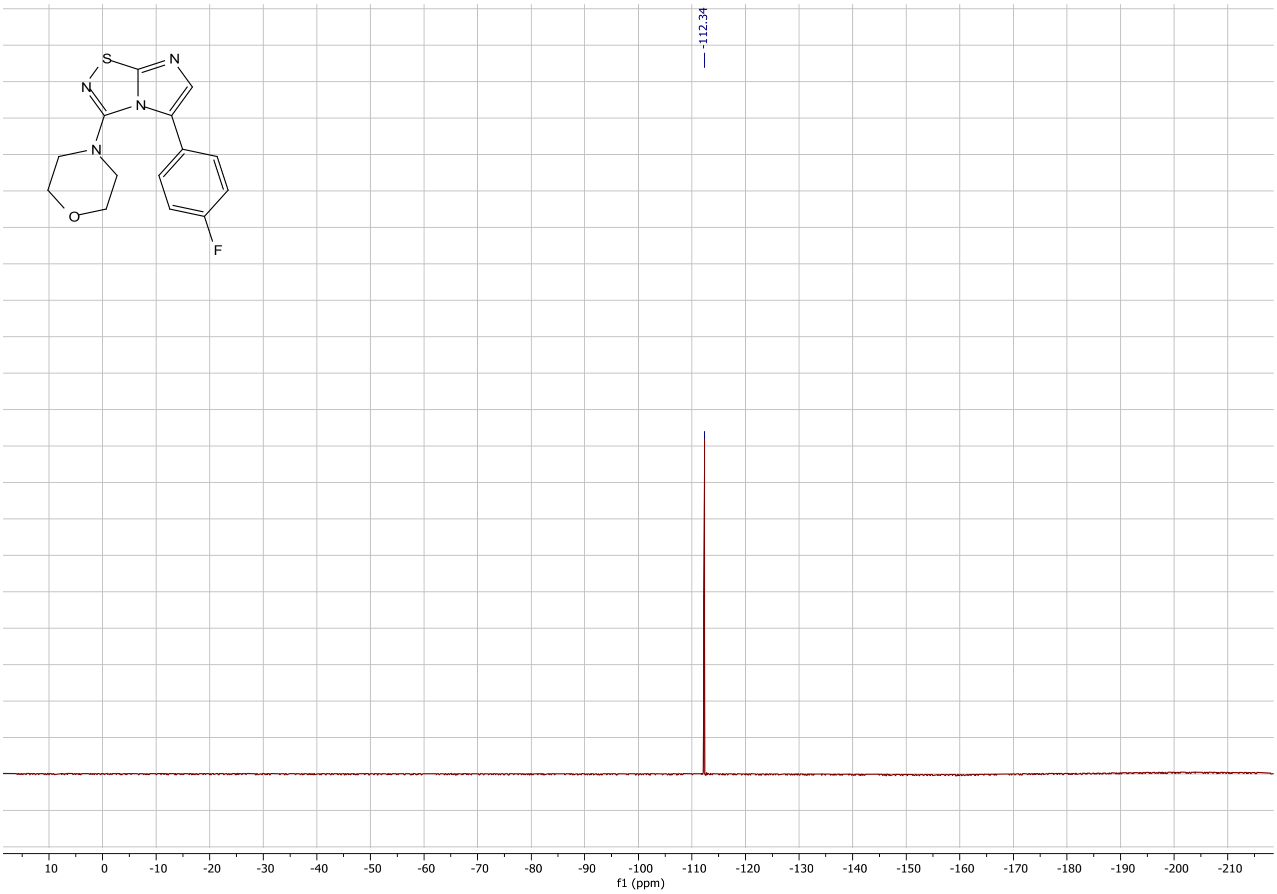

-112.34
S
N
N
N
N
O
F
10
0
-10
-20
-30
-40
-50
-60
-70
-80
-90
-100
-110
-120
-130
-140
-150
-160
-170
-180
-190
-200
-210
f1 (ppm)

4-[5-(3-thienyl)imidazo[1,2-*d*][1,2,4]thiadiazol-3-yl]morpholine (36)

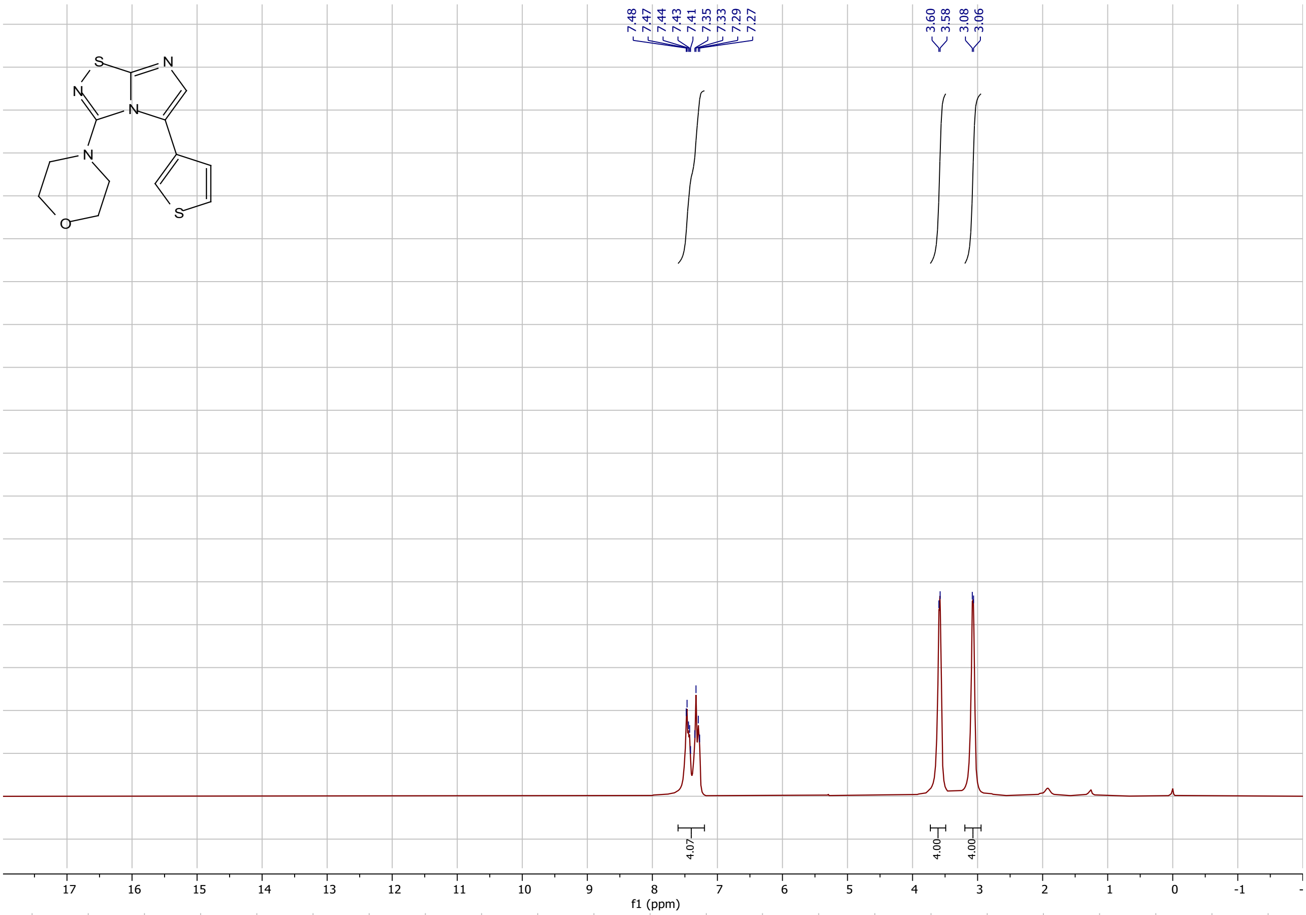

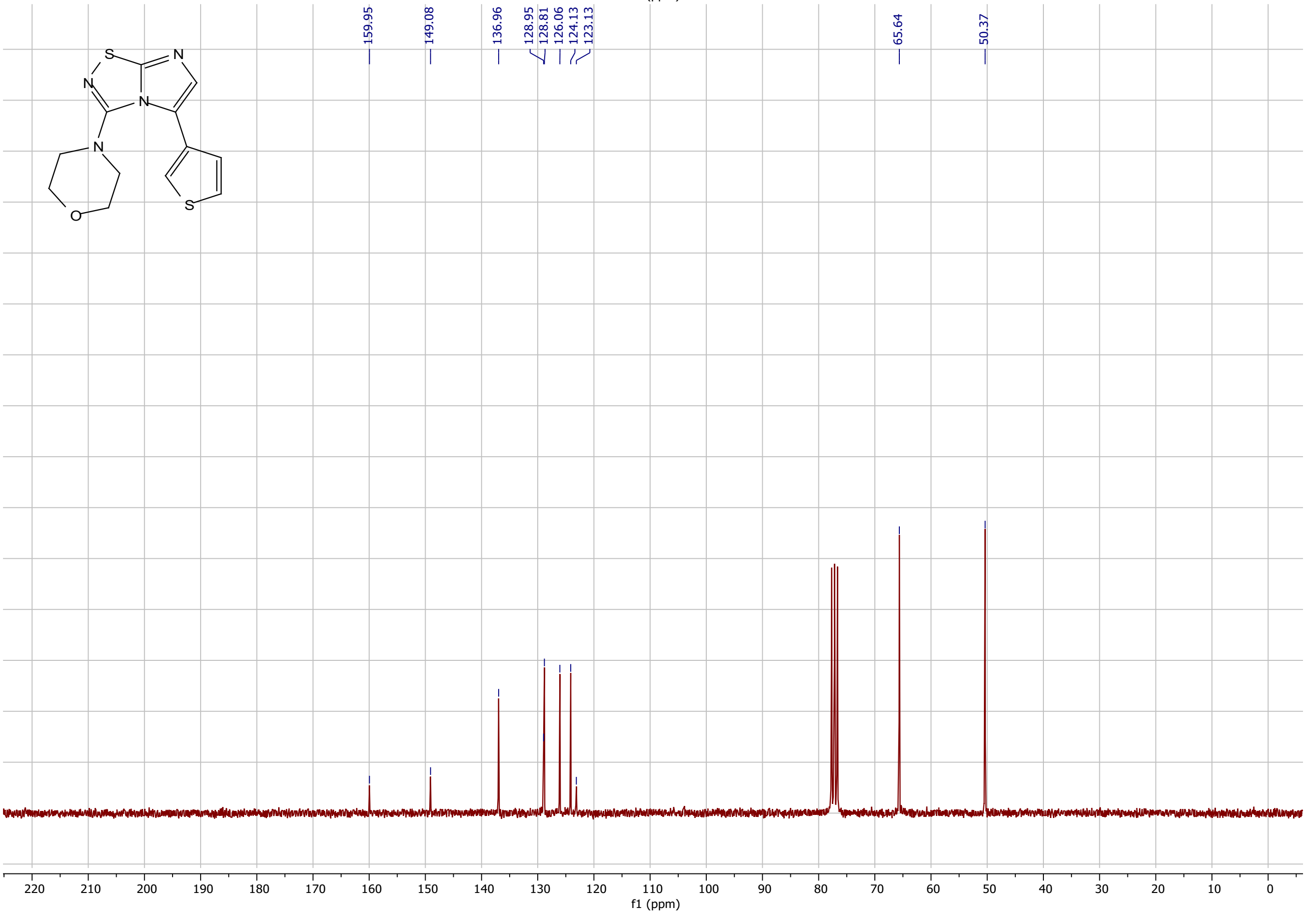

N-methyl-N-propyl-5-(p-tolyl)imidazo[1,2-d][1,2,4]thiadiazol-3-amine (40)

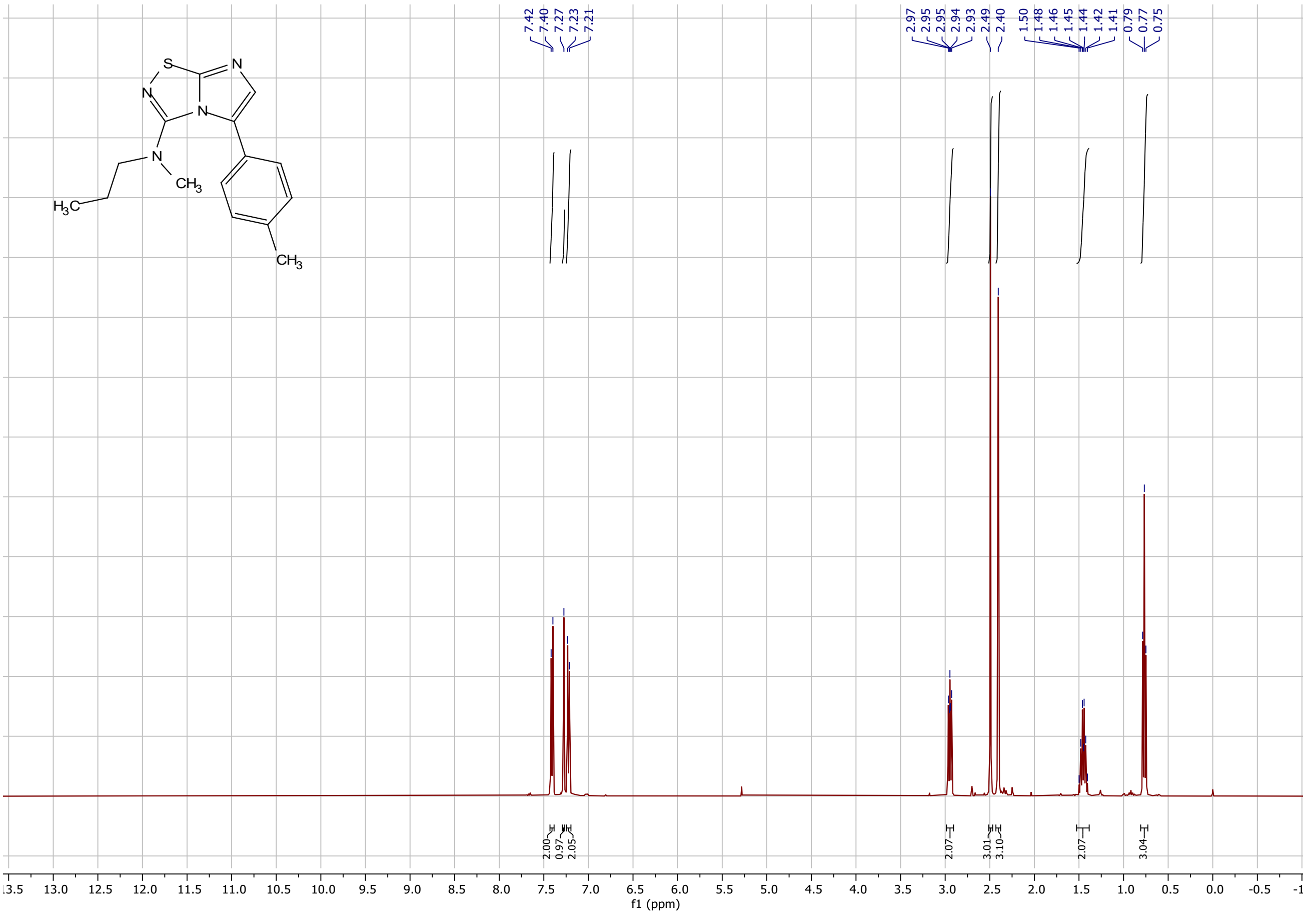

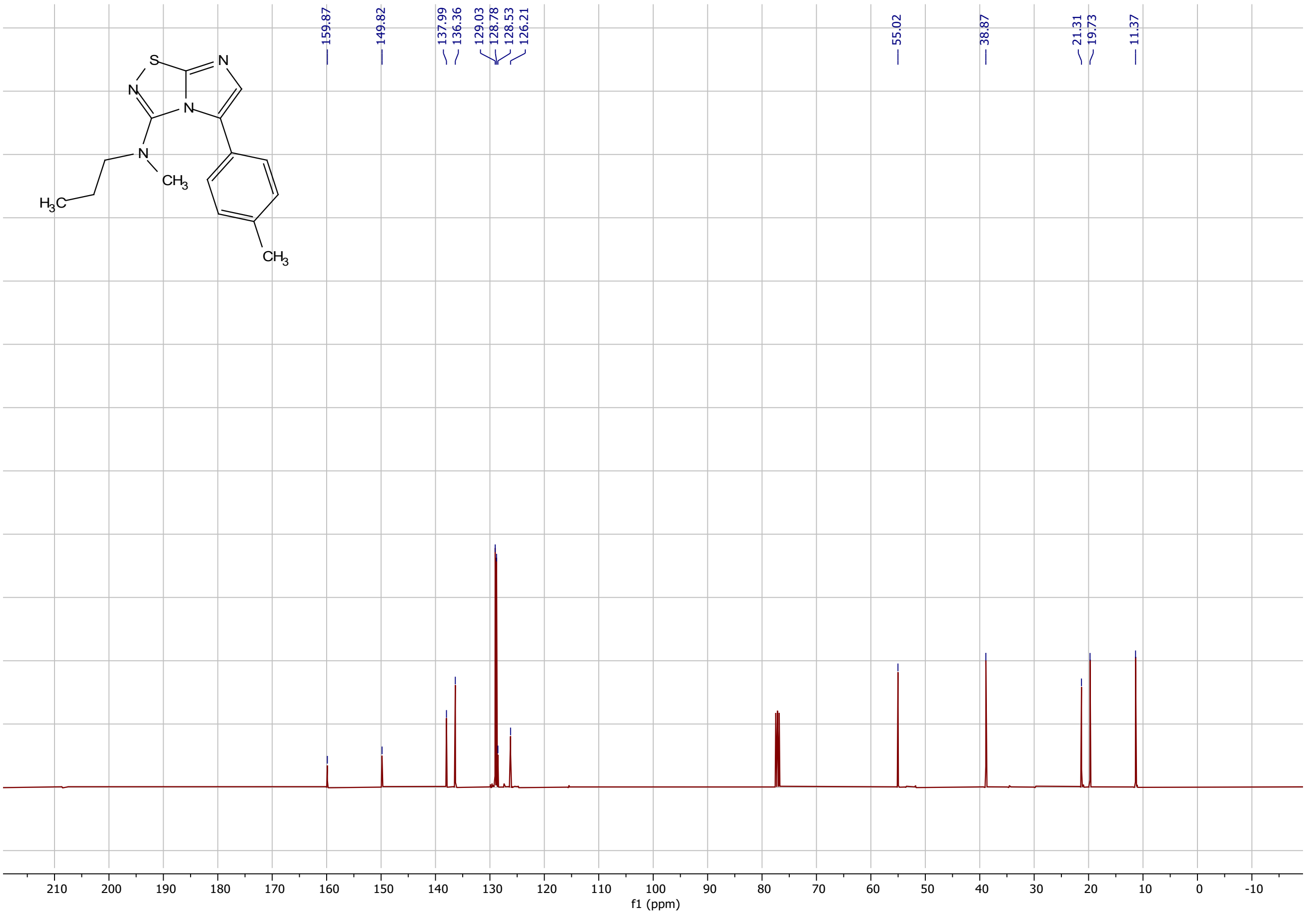
159.87
149.82
137.99
136.36
129.03
128.78
128.53
126.21
55.02
38.87
21.31
19.73
11.37
S
N
N
N
N
CH3
H3C
CH3
210 200 190 180 170 160 150 140 130 120 110 100 90 80 70 60 50 40 30 20 10 0 -10
f1 (ppm)

3-(piperidin-1-yl)-5-(p-tolyl)imidazo[1,2-*d*][1,2,4]thiadiazole (43)

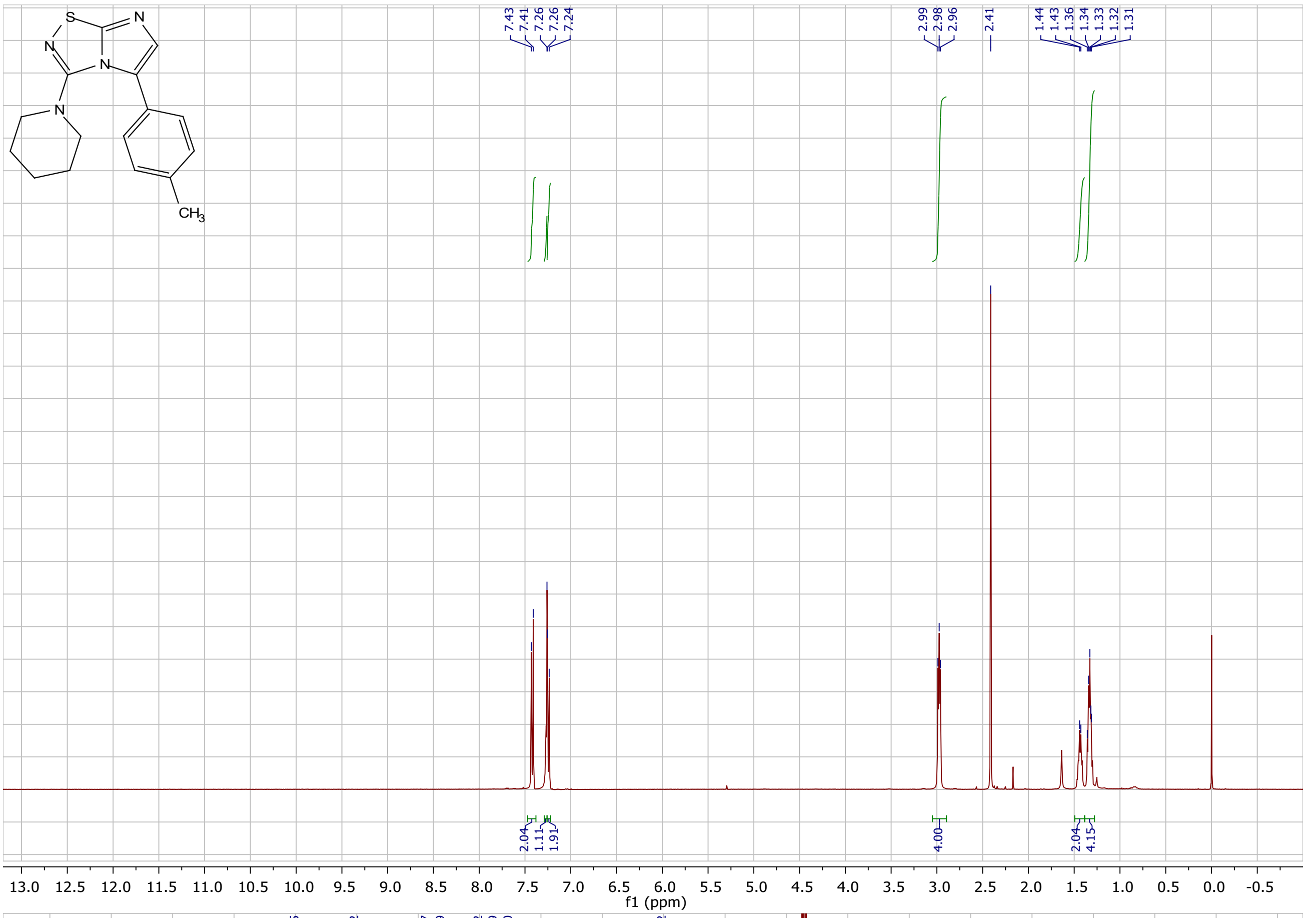

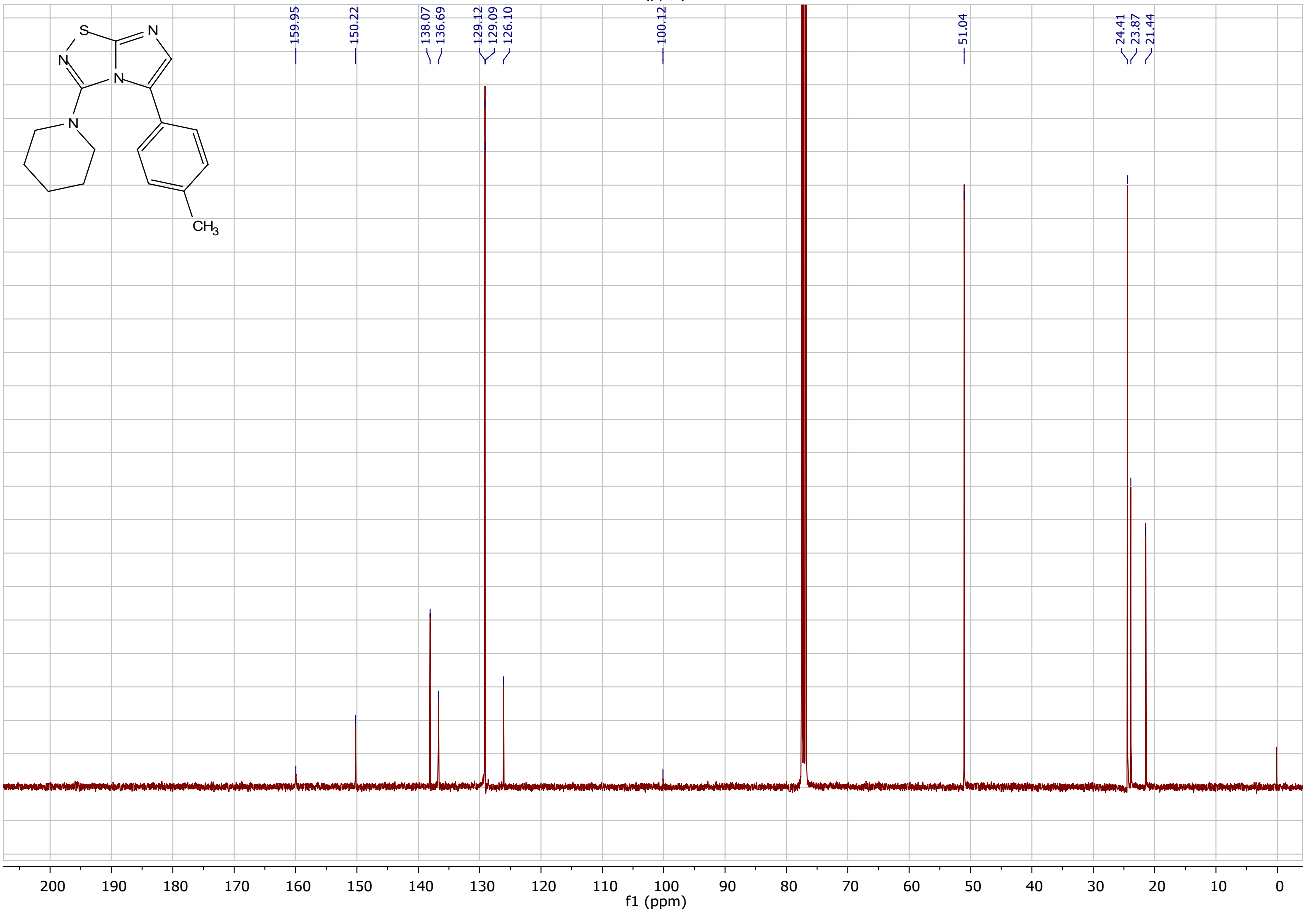

3-(4-methylpiperazin-1-yl)-5-(p-tolyl)imidazo[1,2-*d*][1,2,4]thiadiazole (44)

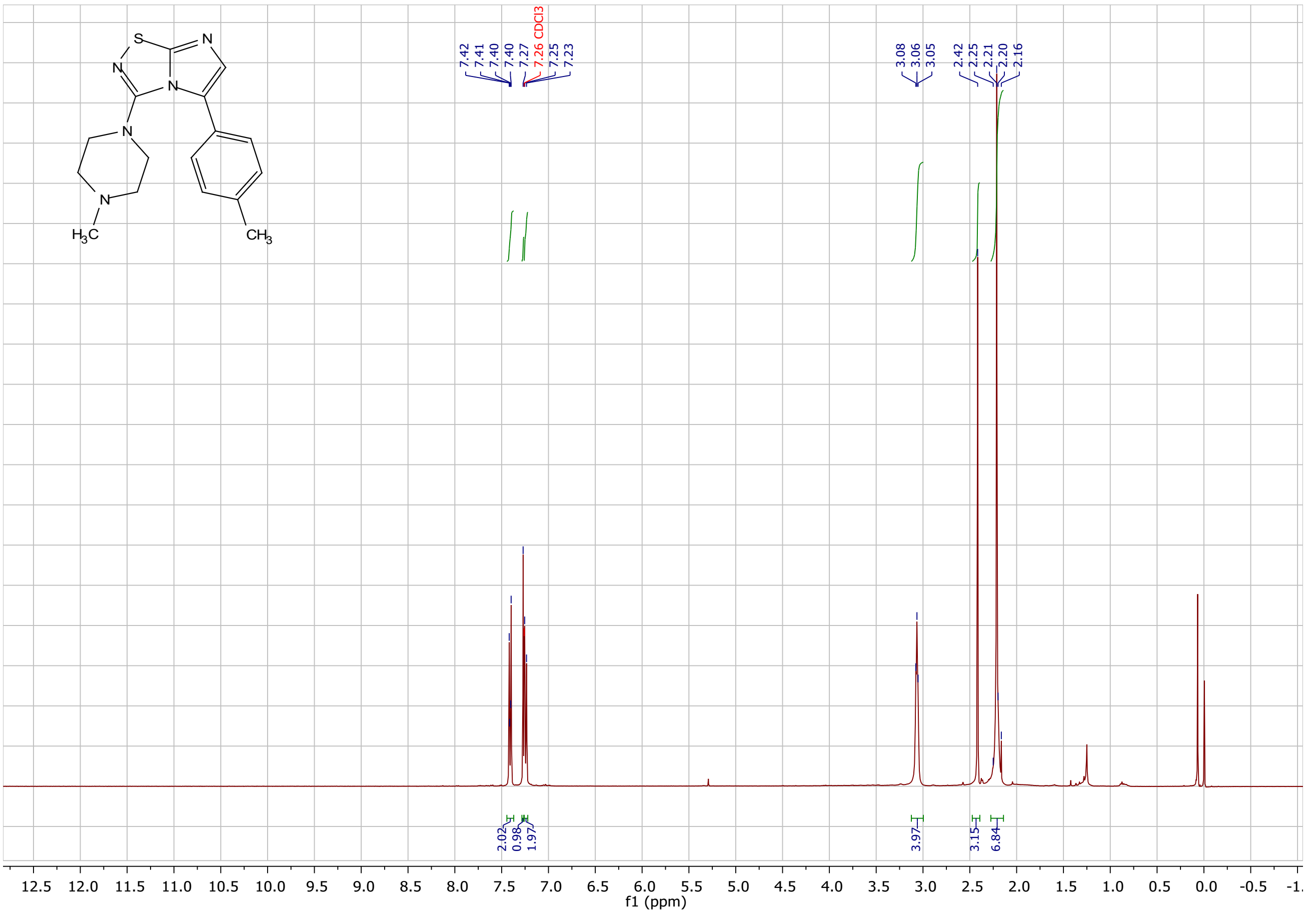

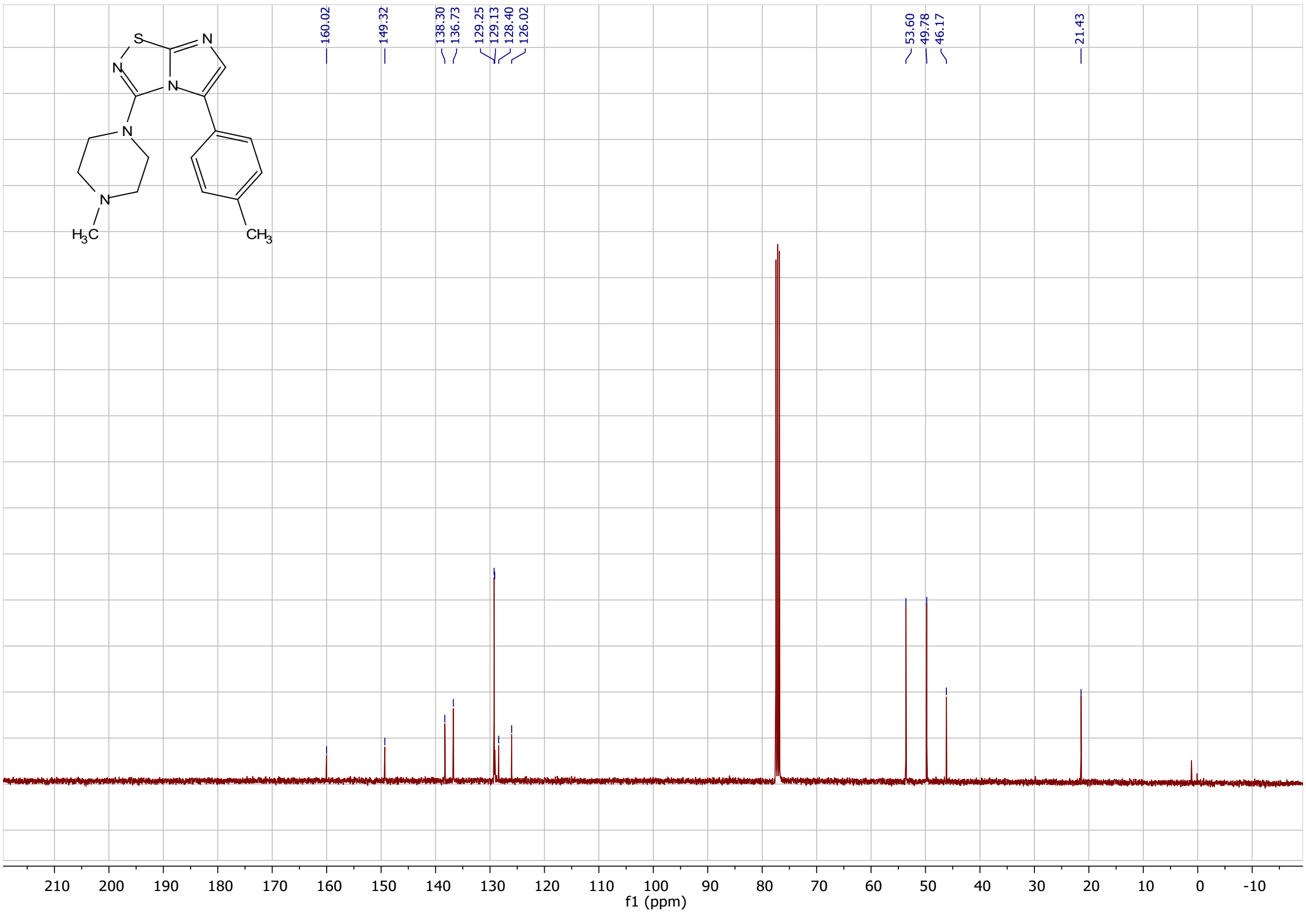

3-Methoxy-5-(*p*-tolyl)imidazo[1,2-*d*][1,2,4]thiadiazole (46)

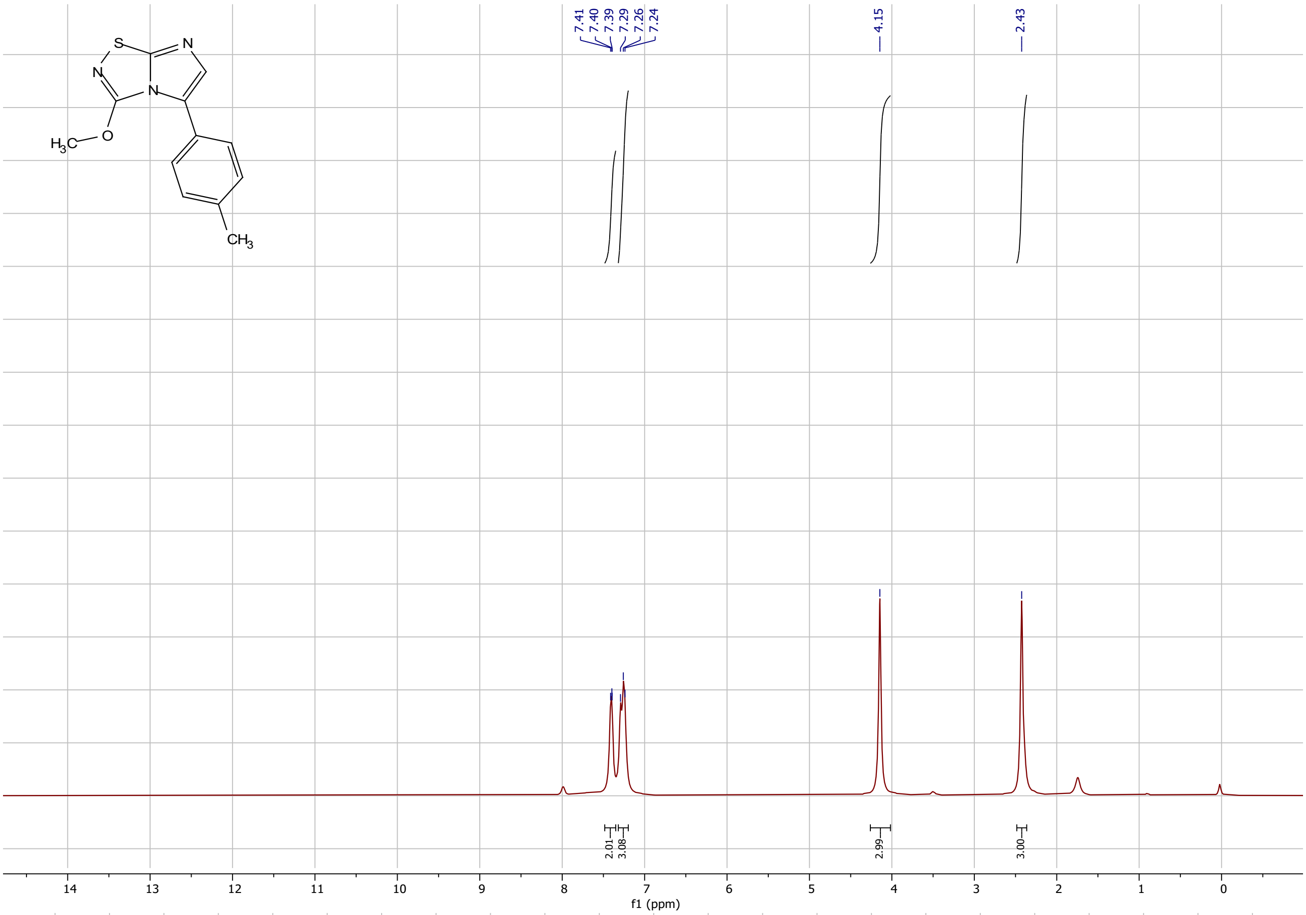
7.41
7.40
7.39
7.29
7.26
7.24
4.15
2.43
2.01
3.08
2.99
3.00
f1 (ppm)

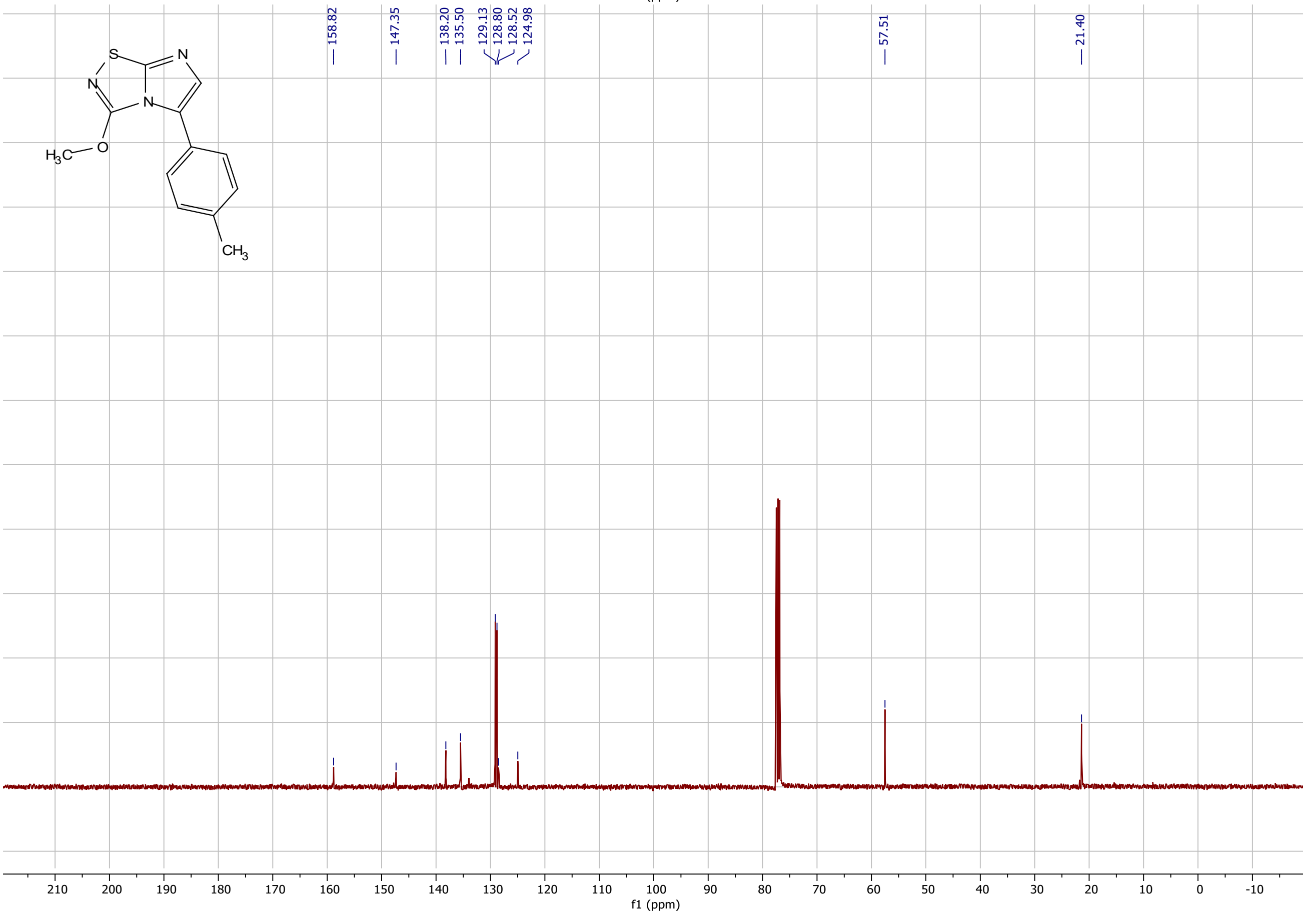
158.82
147.35
138.20
135.50
129.13
128.80
128.52
124.98
57.51
21.40
f1 (ppm)

3-Ethoxy-5-(*p*-tolyl)imidazo[1,2-*d*][1,2,4]thiadiazole (47)

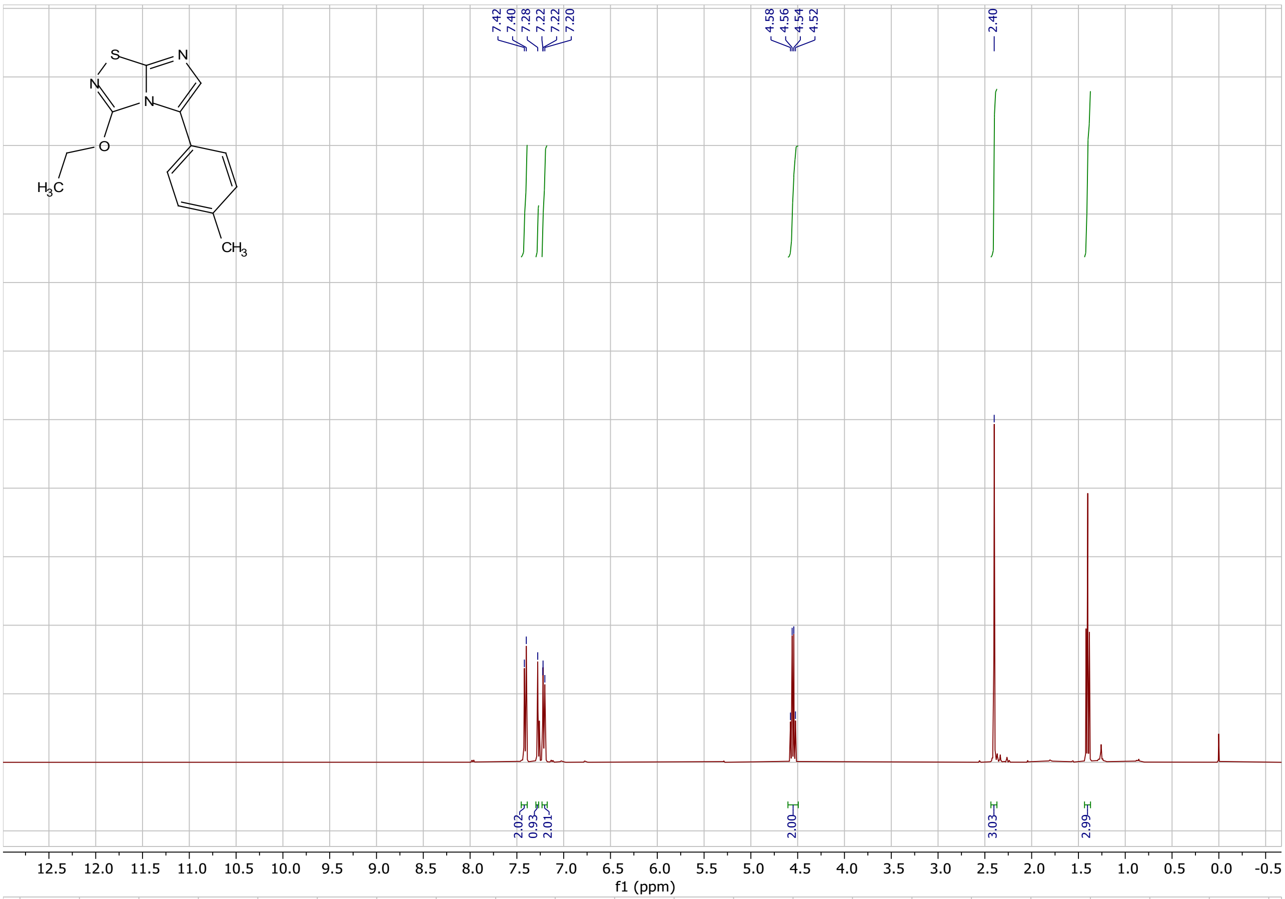

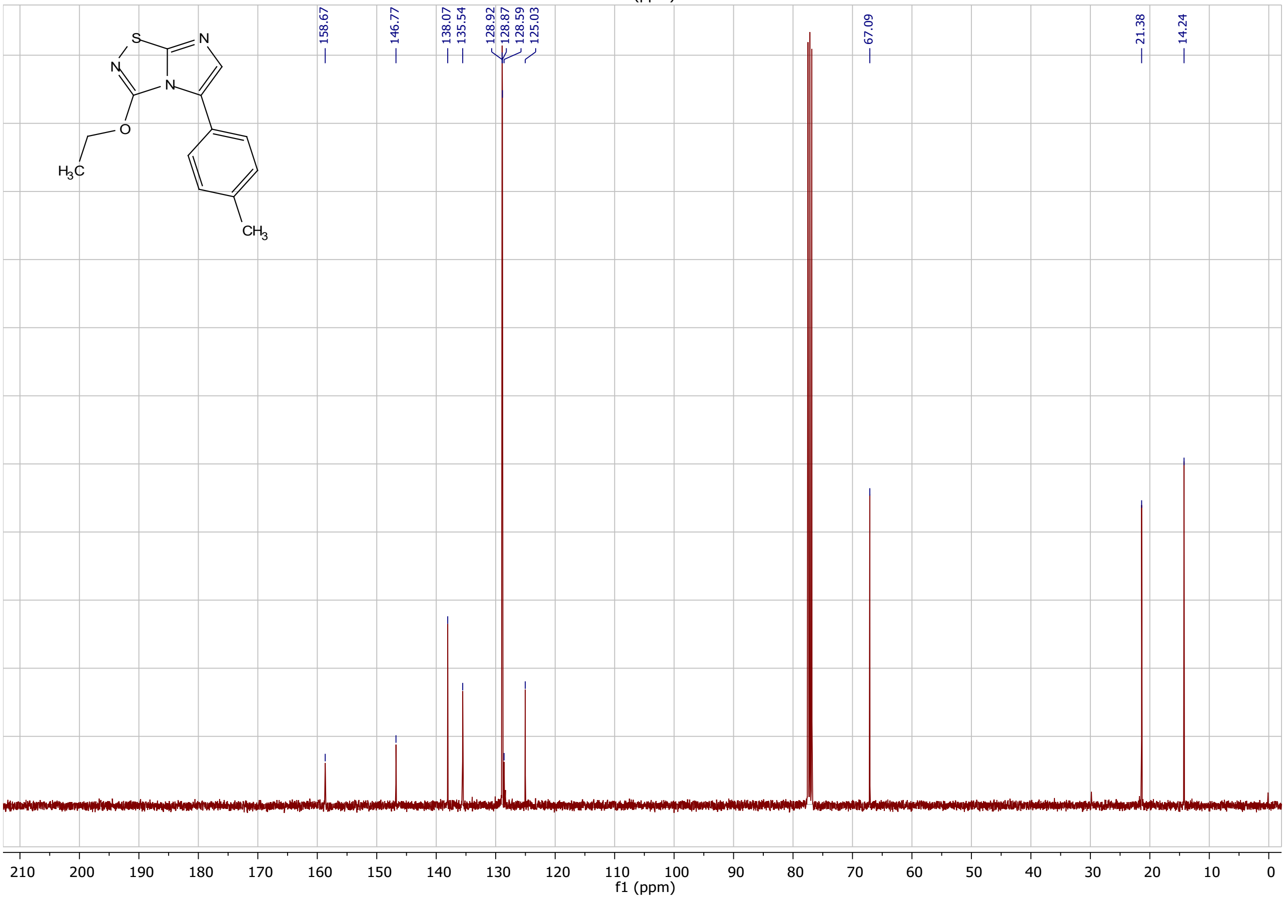

Supplement: RA-012-D1RA07208K-s001 [file RA-012-D1RA07208K-s001.pdf]
